# Draft genome of the honey bee ectoparasitic mite, *Tropilaelaps mercedesae,* is shaped by the parasitic life history

**DOI:** 10.1093/gigascience/gix008

**Published:** 2017-02-22

**Authors:** Xiaofeng Dong, Stuart D. Armstrong, Dong Xia, Benjamin L. Makepeace, Alistair C. Darby, Tatsuhiko Kadowaki

**Affiliations:** 1 Department of Biological Sciences, Xi’an Jiaotong-Liverpool University, 111 Ren’ai Road, Suzhou Dushu Lake Higher Education Town, Jiangsu Province 215123, China; 2 Institute of Infection & Global Health, University of Liverpool, Liverpool L3 5RF, United Kingdom; 3 Institute of Integrative Biology, University of Liverpool, Liverpool L69 7ZB, United Kingdom

**Keywords:** Honey bee decline, Honey bee ectoparasitic mite, Genome, Transcriptome, Proteome, Comparative genomics, Host-Parasite interaction

## Abstract

The number of managed honey bee colonies has considerably decreased in many developed countries in recent years and ectoparasitic mites are considered as major threats to honey bee colonies and health. However, their general biology remains poorly understood. We sequenced the genome of *Tropilaelaps mercedesae*, the prevalent ectoparasitic mite infesting honey bees in Asia, and predicted 15 190 protein-coding genes that were well supported by the mite transcriptomes and proteomic data. Although amino acid substitutions have been accelerated within the conserved core genes of two mites, *T. mercedesae* and *Metaseiulus occidentalis, T. mercedesae* has undergone the least gene family expansion and contraction between the seven arthropods we tested. The number of sensory system genes has been dramatically reduced, but *T. mercedesae* contains all gene sets required to detoxify xenobiotics. *T. mercedesae* is closely associated with a symbiotic bacterium (*Rickettsiella grylli*-like) and Deformed Wing Virus, the most prevalent honey bee virus. *T. mercedesae* has a very specialized life history and habitat as the ectoparasitic mite strictly depends on the honey bee inside a stable colony. Thus, comparison of the genome and transcriptome sequences with those of a tick and free-living mites has revealed the specific features of the genome shaped by interaction with the honey bee and colony environment. Genome and transcriptome sequences of *T. mercedesae*, as well as *Varroa destructor* (another globally prevalent ectoparasitic mite of honey bee), not only provide insights into the mite biology, but may also help to develop measures to control the most serious pests of the honey bee.

## Introduction

**Figure 1:**
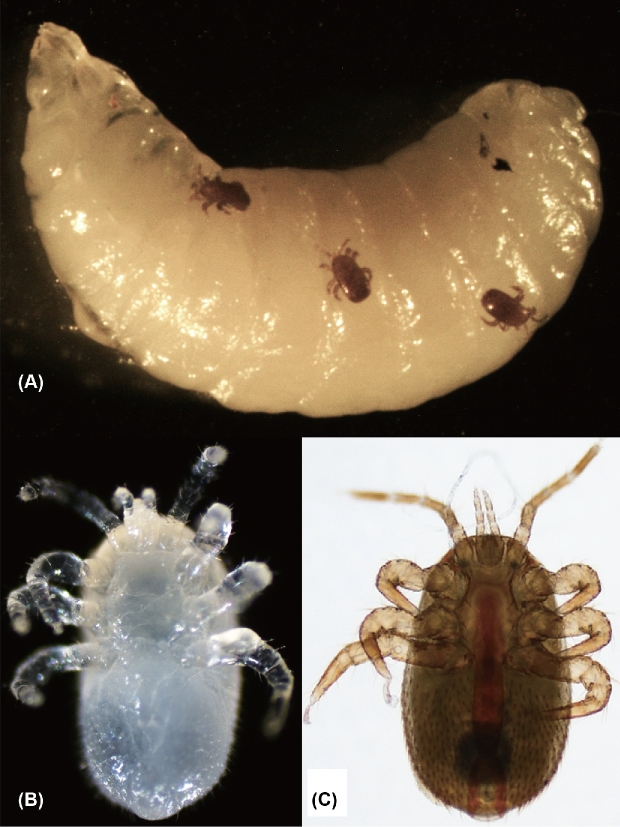
Images of Tropilaelaps mercedesae. (A) Three adult females of *T. mercedesae* infesting the 5th instar honey bee larva. (B) Ventral view of the nymph (immature female). (C) Ventral view of the adult female.

The number of managed honey bee (*Apis mellifera*) colonies has considerably decreased in many developed countries in recent years [1]. Although there are many potential causes for the decline, pathogens and parasites of the honey bee, particularly ectoparasitic mites, are considered major threats to honey bee colonies and health [2]. *Varroa destructor* is present globally and causes abnormal brood development and brood death in honey bees, and is also responsible for the spread of honey bee pathogens and parasites [3]. *Tropilaelaps mercedesae* (small honey bee mite, Fig. 1) is another honey bee ectoparasitic mite that is prevalent in most Asian countries [4]. Thus, these two mite species usually coexist in a honey bee colony in Asia. Compared to *V. destructor*, *T. mercedesae* produces a higher number of offspring and has almost no phoretic period on adult honey bees, and thus builds up relatively higher population levels within colonies [4, 5]. Similar to *V. destructor*, *T. mercedesae* can vector Deformed Wing Virus (DWV) [6, 7] and influence host immune responses [8]. Furthermore, it has been recently shown that *T. mercedesae* infestation reduces the longevity and emergence weight of honey bees, and enhances the DWV levels and associated symptoms [9]. The original host of *T. mercedesae* is the giant Asian honey bee, *Apis dorsata*, and like *V. destructor*, it shifted hosts to infest *A. mellifera* when these colonies were brought into Asia [4]. Although *T. mercedesae* is currently restricted to Asia, it has the potential to spread and establish all over the world due to the global trade of honey bees. This is exactly what happened with *V. destructor* [10].

*T. mercedesae* and *V. destructor* are major threats to the current apiculture industry; however, we still do not completely understand their sensory system, development, sex determination/differentiation, reproduction, and the capability to acquire miticide (for example, tau-fluvalinate and flumethrin) resistance. Genomic features of *V. destructor* were briefly reported before and the associated bacteria and viruses were identified [11]. In this study, we sequenced the genome and transcriptomes of *T. mercedesae*, supplemented by proteomic data, to provide insights into the above aspects and understand how the mite has evolved under a very specialized environment, inside the honey bee colony by depending on the honey bee as the sole host. We will discuss how *T. mercedesae* may have adapted to its host and environment by shaping its genome.

## Results and discussion

### Genome assembly, repeated sequences, and gene annotation

Dual indexed paired-end DNA libraries were prepared from a single adult male and female *T. mercedesae* for whole-genome sequencing using the Illumina shotgun platform (Supplementary Table S1). The “cleaned” reads from the male mite were then reassembled into 34 155 scaffolds with an N50 of 28 807 bp representing ∼353 Mb of genomic sequence, from which we predicted 15 190 protein-coding genes (Table 1; Supplementary Table S2). We found that 95.33% of the “cleaned reads” could be mapped back to this assembly and 244 (98.4%) of the 248 Conserved Eukaryotic Genes [12] as well as 83% of 2675 arthropod BUSCOs [13] were annotated from the assembled genome (Supplementary Table S3). These are comparable to those reported for nine other arachnids (Table 1; Supplementary Table S3). Proteomic characterization of the adult males and females yielded 124 798 mass spectra in total and 60 463 were assigned to the peptides of annotated proteins above (Supplementary file 1). With k-mer statistics [14], the size of the *T. mercedesae* genome was estimated to be 660 Mb with a peak k-mer depth of ∼60×, and thus approximately 50% of the genome DNA was inferred to comprise repetitive sequences (Supplementary Fig. S1). Repetitive sequences such as DNA transposons, retrotransposons including Long Interspersed Nuclear Element, Short Interspersed Nuclear Element, and Long Terminal Repeat as well as satellite DNA represent only 7% of the assembly. But they occupied 48.57% of total clean reads (Supplementary Table S4) and the majority of them were found in the high-coverage regions of the genome (Supplementary Table S5), suggesting that repetitive sequences have been collapsed in the genome assembly. We thus concluded that the qualities of draft genome sequence and protein-coding gene set were sufficiently robust for further characterization of *T. mercedesae* genome and transcriptome.

**Table 1:** Genome statistics of *T. mercedesae* and other arachnid species.

	Acari: Parasitiformes				Acari: Acariformes			Araneae		Scorpiones
	*Tropilaelaps*	*Metaseiulus*	*Varroa*	*Ixodes*	*Dermatophagoides*	*Sarcoptes*	*Tetranychus*	*Stegodyphus*	*Acanthoscurria*	*Mesobuthus*
Species	*mercedesae*	*occidentalis*	*destructor*	*scapularis*	*farinae*	*scabiei*	*urticae*	*mimosarum*	*geniculata*	*martensii*
Estimated genome size (Mb)	660	88-90	565	2100	–	98	90	2550	6500	1323
Assembled genome Size (Mb)	353	152	294	1765	54	56	91	2739	7178	926 1129
GC content (%)	44	52	41	45	30	38	32	34	39	30
Total scaffold number	34 155	2 211	na	369 492	515	18 860	640	68 653	4 986 575	na
Largest scaffold (kb)	327 111	2 438 724	na	3 698 136	771 048	287 415	6 836 010	2 994 948	819 799	340 307
N50 size (bp)	28 807	896 831	na	76 228	186 342	Na	2 993 488	480 636	47 837	223 560
Complete CEGs (%)	92	98	68	80	98	98	98	62	33	57
Partial CEGs (%)	98	97	32	42	96	94	95	24	15	24
Number of protein-coding genes	15 190	18 338 11 430	11 432	20 486	16 376	10 473 10 644	18 414 18 224	27 135	27 235	73 821
Average exon length (bp)	363	262	na	187	na	347	334	174	Na	Na
Average intron length (bp)	820	647	na	2653	na	147	477	4269	Na	Na

Data refer to this study and [11, 15, 16, 19, 81, 101].

Flow cytometric measurement of *T. mercedesae* nuclear DNA content together with the k-mer statistics demonstrated that the male mite, which was assumed to be haploid, had a genome size of ∼660 Mb (1C) DNA. The female mite was twice that size and assumed to be diploid at 1287 Mb (2C) DNA (Supplementary Fig. S2). Thus, *T. mercedesae* may use haplodiploidy for sex determination, and the genome size of *T. mercedesae* is the largest among those of mites whose genomes have been sequenced (*V. destructor*, *Metaseiulus occidentalis*, *Tetranychus urticae*, *Sarcoptes scabiei*, and *Dermatophagoides farinae*) [15–17, 11, 18] but smaller than those of ticks (e.g., *Ixodes scapularis* [19]). As expected from the largest genome size among the sequenced mites, gene density is low in the *T. mercedesae* genome (with larger intergenic regions), reminiscent of the large velvet spider (*Stegodyphus mimosarum)* and the black-legged tick (*I. scapularis)* genomes (Supplementary Fig. S3). Although the exon size range was comparable in all tested genomes (small honey bee mite, predatory mite, black-legged tick, velvet spider, spider mite, fruit fly, and honey bee) (Supplementary Fig. S4A), the average size of introns in *T. mercedesae* is larger than that in two other mites and insects that were analyzed (Supplementary Fig. S4B). We also successfully annotated genes encoding rRNA, tRNA, snRNA, and miRNA in the *T. mercedesae* genome (Supplementary Table S6), obtained RNA-seq data from *T. mercedesae* adult males and females as well as nymphs, and assembled the reads to aid protein-coding gene annotation and to compare their gene expression profiles.

### Comparative genomics

**Figure 2:**
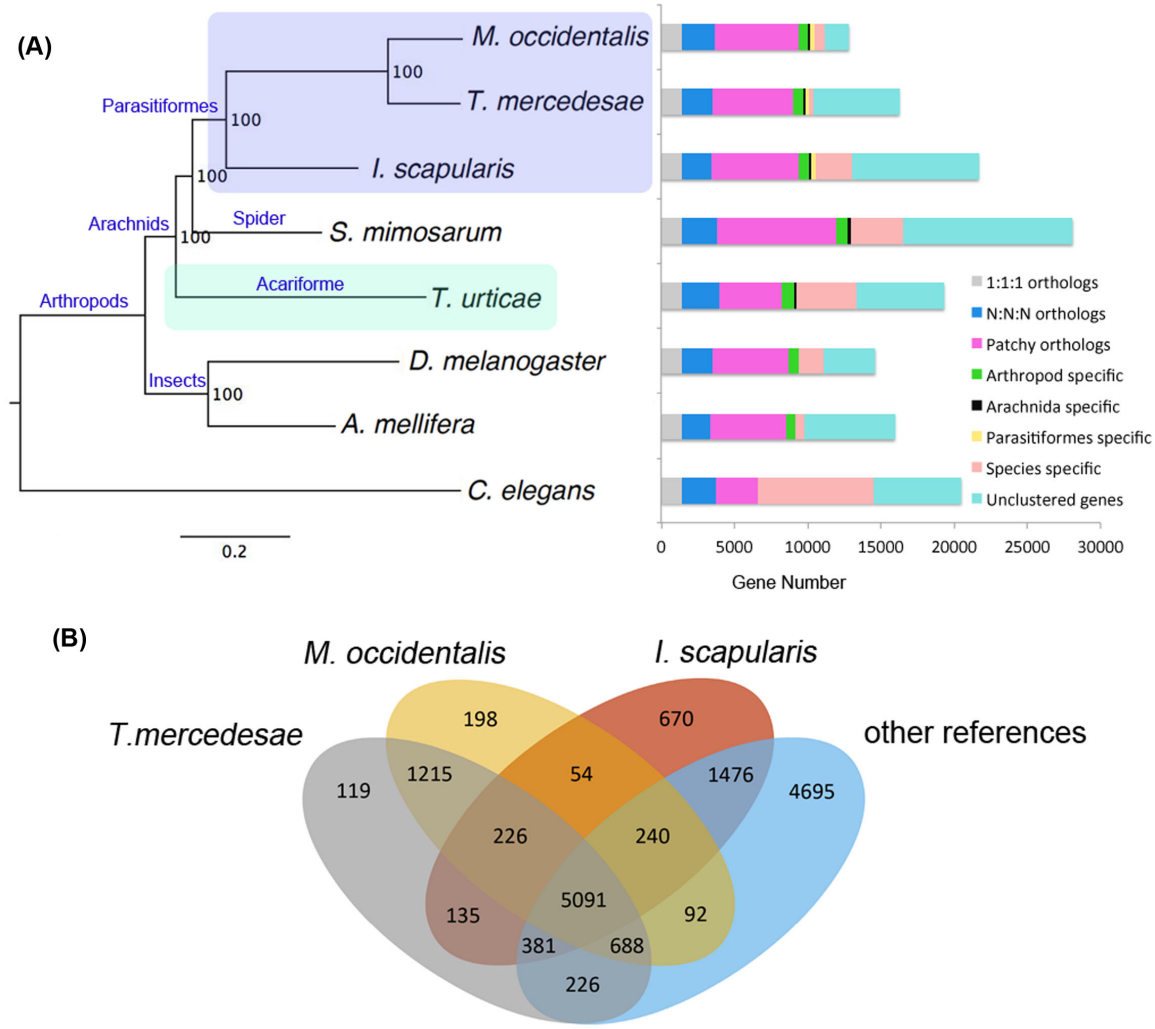
Comparative genomics. (A) The species phylogeny was built from aligned protein sequences of 926 one-to-one orthologs in *Metaseiulus occidentalis*, *Tropilaelaps mercedesae*, *Ixodes scapularis*, *Stegodyphus mimosarum*, *Tetranychus urticae*, *Drosophila melanogaster*, *Apis mellifera*, and *Caenorhabditis elegans* using a maximum likelihood method. The tree was rooted with *C. elegans*. All nodes showed 100% bootstrap support. Protein-coding genes were classified into the different categories. 1:1:1 orthologs and N:N:N orthologs represent the common orthologs with the same copy numbers and different copy numbers, respectively. Patchy orthologs are shared between more than one but not all species (excluding those in the previous categories). Unclustered genes represent genes which were not classified into orthology cluster. Other categories include arthropod-, Arachnida-, Parasitiformes-, and species-specific genes. *C. elegans* was used as the outgroup for classification of the protein-coding genes. (B) The number of gene families shared between *T. mercedesae*, *M. occidentalis*, *I. scapularis*, and other reference species (*S. mimosarum*, *T. urticae*, *D. melanogaster*, *A. mellifera*, and *C. elegans*) by orthoMCL classification algorithm.

The protein-coding genes of *T. mercedesae* were compared with those of six other arthropods (mentioned above) and a nematode. Phylogenetic trees constructed using 926 highly conserved 1:1 orthologs implementing both maximum likelihood and Bayesian methods demonstrated that the *Tropilaelaps* mite and the predatory mite cluster together; however, the spider mite forms an outgroup to two other mites, the black-legged tick, and the velvet spider (Fig. 2A). This is consistent with previous reports that the subclass Acari is diphyletic, with the superorders Acariformes (spider mite) and Parasitiformes (*Tropilaelaps* mite and predatory mite) being distantly related [20, 21]. Since the above three mite species have similar body structure and morphology, this could be an example of convergent evolution [22]. The molecular species phylogenetic tree also indicates the variable evolutionary rates in gene sequence, with the branch of *T. mercedesae* and *M. occidentalis* exhibiting the fastest rate among arthropods we tested (Fig. 2A).

OrthoMCL classified the predicted proteins of *T. mercedesae* together with proteins from six other arthropods and outgroup into a total of 15 506 orthology clusters. As expected from the phylogenetic tree, the *Tropilaelaps* mite shares the most orthology clusters (1215) with the predatory mite (Fig. 2B). Among these orthology clusters, GO terms related with ‘Structural constituent of cuticle,’ ‘Regulation of DNA methylation,’ and ‘Xenobiotic metabolic process’ are enriched (Supplementary Table S7). We found 6178 genes are only present in *T. mercedesae* but not in the other reference genomes analyzed (Fig. 2A and B). These *T. mercedesae* specific genes may include both *T. mercedesae*-unique genes and paralogs that have extensively diverged from their orthologs such that their sequence similarity was not detected by orthoMCL. We found that 1981 *T. mercedesae* specific genes could be assigned with at least one GO term and among these lineage-specific genes, three GO terms, ‘Structural constituent of cuticle,’ ‘Nucleosome,’ and ‘DNA bending complex’ are highly enriched (FDR < 1.50 E^−04^) (Supplementary Table S8). *T. mercedesae* contains 117 members of the cuticle protein family [23], in which 53 are novel among the 7 arthropods analyzed, suggesting that the mite's exoskeleton has rapidly evolved. Two other enriched GO terms could be involved in the epigenetic control of gene expression. Among 226 orthology clusters that are shared between *T. mercedesae*, *M. occidentalis*, and *I. scapularis*, GO terms related with ‘Transporter activity’ are highly enriched. We found that 135 orthology clusters specifically shared between *T. mercedesae* and *I. scapularis* were enriched with GO terms related to ‘Renal tubule development,’ perhaps to maintain a constant water level following the intake of a large volume of hemolymph or blood, respectively [24, 25] (Supplementary Table S9).

We used CAFE to infer gene family expansion and contraction in *T. mercedesae* together with six other arthropod species. We found that *T. mercedesae* has undergone the fewest gene family expansion/contraction events since divergence from the common ancestor of arthropods (Supplementary Fig. S5). This feature may fit to the specific life history of a mite parasitizing only the honey bee and living inside a colony with an enclosed, stable environment. However, there are some significantly expanded gene families (*P* value < 0.001) associated with zinc ion binding and peptide cross-linking. Meanwhile, one of the HSP70 gene families (Heat shock 70 kDa protein cognate 4) has significantly contracted in *T. mercedesae* (Supplementary Table S10), perhaps because the mite spends most of its time in the honey bee brood cell where the temperature is constantly around 35°C [26]. We analyzed 91 genes with d*_N_*/d*_S_* > 1.0 in *T. mercedesae* using the one ratio model (null model) to test the significance, and found that four genes have evolved rapidly either due to relaxation or positive selection (Supplementary Table S11). Among them, Tm_07523 encodes an endo-β-N-acetylglucosaminidase-like protein, a chitinase, which could be involved in processing chitin specifically present in *T. mercedesae*.

### Sensory systems

*T. mercedesae* has a very specific life history and habitat as a honey bee ectoparasitic mite. The mite depends only on the honey bee as the host and spends most of its life in the capped brood cell. Thus, they are likely to depend on the chemosensory rather than the visual system to seek out the fifth instar honey bee larva and the mating pair. Therefore, we annotated and analyzed genes associated with phototransduction and chemosensory systems in *T. mercedesae.*

We found that the homologs of *D. melanogaster* opsins, arrestin, TRPL, and INAD are absent in *T. mercedesae* (Supplementary Fig. S6). Since they are the major components for fruit fly photoreception, *T. mercedesae* appears to be blind, and this is consistent with the lack of eye structures. Nevertheless, the adult females immediately move out from a brood cell when the cap is removed and exposed to light, suggesting that they may be able to respond to light. *T. mercedesae* has two *peropsin* genes, as do predatory mites [21] (Supplementary Fig. S7). Peropsin is a retinal photoisomerase that converts all-*trans-*retinal to 11-*cis*-retinal and may couple with a G-protein through the conserved 'NPXXY’ motif at the seventh transmembrane domain [27]. The existence of this gene in the jumping spider, black-legged tick, and humans suggests that peropsin may have been lost specifically in insects. However, its function in vision or other pathways remains to be determined. Only one of two *peropsin* genes (Tm_08036) appears to be expressed in the *T. mercedesae* transcriptome, and it was highly expressed in the female compared to the male (Supplementary Fig. S8). Female may use this peropsin to move out from the brood cell for reproduction. The other components in phototransduction are present in *T. mercedesae*, suggesting that they could be involved in other signaling pathways. In contrast to *T. mercedesae*, *M. occidentalis* was reported to contain more molecular components for light perception such as arrestins and INAD and exhibit genuine light-induced behaviors in the absence of eyes [21]. Meanwhile, *I. scapularis* contains seven opsins, including orthologs of the insect long-wavelength sensitive visual opsins [19], demonstrating that the tick uses more visual cues for location of mates, hosts, and oviposition sites than the mites above.

**Figure 3:**
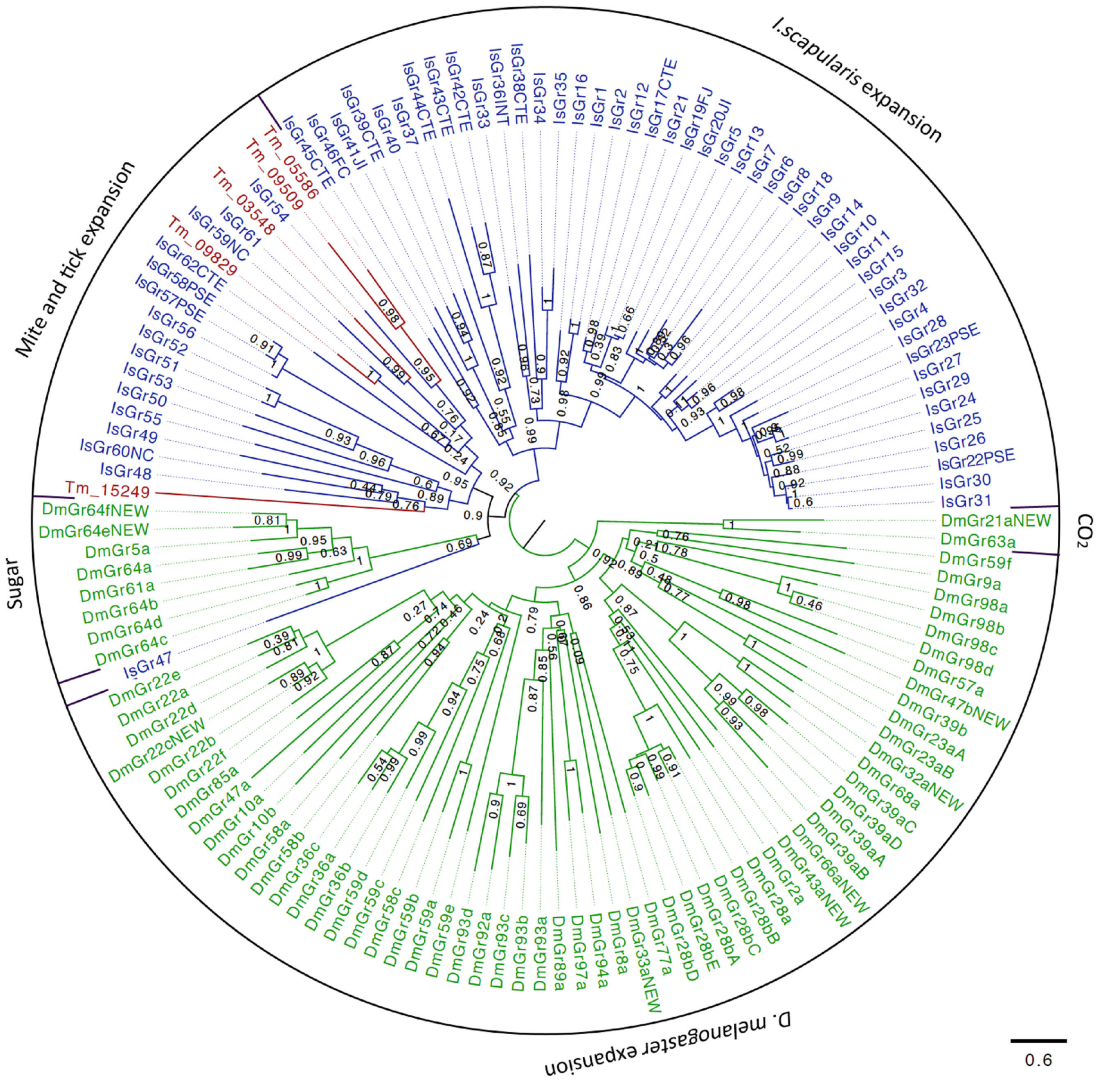
Phylogenetic tree of *T. mercedesae*, *I. scapularis*, and *D. melanogaster* gustatory receptors. Phylogenetic tree of *T. mercedesae* (red), *I. scapularis* (blue), and *D. melanogaster* (green) GRs was constructed by a maximum likelihood method. Two clusters of fruit fly GRs responding to sugar and CO_2_ are indicated. The tree was rooted at the middle point.

Insect gustatory receptors (GRs) are multifunctional proteins for the perception of taste, airborne molecules, and heat [28]; however, their functions in other arthropods have not been addressed. We found only five GRs in *T. mercedesae* (TmGRs) and their orthologs are absent in *D. melanogaster* (Fig. 3). *I. scapularis* has expanded the specific group of GRs [19], and five TmGRs cluster with the tick's GRs, suggesting that these are expansions specific to Acari. Because they share a common ancestor with the *D. melanogaster* sugar receptor, they could be involved in taste perception (Fig. 3). Among the five TmGRs, one gene (Tm_15249) is likely to be a pseudogene due to internal stop codons in the open reading frame. Expression of only two TmGR genes (Tm_03548 and Tm_09509) was supported by RNA-seq data. Tm_09509 mRNA is highly expressed in adult females and Tm_03548 mRNA is only detected in males at low levels (Supplementary Fig. S9), suggesting that they may respond to different ligands.

Ionotropic receptors (IRs) belong to a large family of ligand-gated ion channels, which also include ionotropic glutamate receptors (iGluRs) with the major roles in synaptic transmission. IRs appear to represent protostome-specific ancient olfactory and gustatory receptors [29]. We annotated eight IR and 34 iGluR genes in the *T. mercedesae* genome. In the eight annotated *T. mercedesae* IR (TmIR) genes, Tm_15231 and Tm_15229 are orthologs of DmIR25a and DmIR93a, respectively (Supplementary Fig. S10), which are expressed in the olfactory sensory neurons of *D. melanogaster* antennae [30]. Furthermore, DmIR25a has been recently shown to be involved in fruit fly temperature sensation [31, 32]. The results of qRT-PCR revealed that these two genes are highly expressed in the first legs of *T. mercedesae* (Supplementary Fig. S11), which function as the major sensory organs similar to insect antennae [33]. Thus, these two TmIRs may represent the ancient receptors present in the common ancestor of arthropods. It appears that six other TmIRs have arisen specifically in a mite lineage (Supplementary Fig. S12).

Interestingly, there are no OR (olfactory receptor), OBP (odorant binding protein), and CSP (chemosensory protein) genes in the *T. mercedesae* genome (Table 2). Since OR and OBP genes are also absent in *M. occidentalis*, the black-legged tick, the centipede (*Strigamia maritima*), and the water flea (*Daphnia pulex*), these appear to have evolved specifically in insect genomes as previously suggested [34]. Nevertheless, CSP genes must be ancient and may have been specifically lost in the two mite species. Despite the potential importance of chemical communication for the life cycle [4], *T. mercedesae* has only four functional GRs and eight IRs, but no OR, OBP, or CSP genes. The presence of few orthologs between *T. mercedesae* and *D. melanogaster* suggests that the last common ancestor of arthropods had very few GRs and IRs. These chemoreceptors appear to have expanded in arthropod species in a lineage-specific manner [35]. In fact, Parasitiformes exposed to more variable environments, *i.e.*, *M. occidentalis* and *I. scapularis*, have more GR and IR genes than the more strictly host-dependent *T. mercedesae* (Table 2). Simplified behavioral patterns under a dark and stable environment inside a honey bee colony and capped brood cell may have reduced the number of tools in the sensory system in *T. mercedesae*.

**Table 2:** The number of genes associated with chemosensory system in *T. mercedesae* and other arthropods.

Species	GR	OR	IR	OBP	CSP
*T. mercedesae*	5	0	8	0	0
*M. occidentalis*	64	0	65	0	0
*I. scapularis*	60	0	22	0	1
*S. maritima*	77	0	60	0	2
*D. pulex*	53	0	85	0	3
*D. melanogaster*	73	62	66	51	4
*A. mellifera*	10	163	10	21	6
*B. mori*	56	48	18	44	18
*A. pisum*	53	48	11	15	13
*P. humanus*	8	10	12	5	7

The numbers of GR (gustatory receptor), OR (olfactory receptor), IR (ionotropic receptor), OBP (olfactory binding protein), and CSP (chemosensory protein) genes in *T. mercedesae* and nine arthropod species including *Bombyx mori* and *Acyrthosiphon pisum* are shown. Data refer to references [35, 102, 21] and this study.

### Detoxification system

Three major groups of enzymes have important roles for metabolizing toxic xenobiotics in insects and the acquisition of insecticide resistance; cytochrome P450s (P450s), glutathione-S-transferases (GSTs), and carboxylesterases (CCEs) [36]. P450s and CCEs are also involved in the synthesis and degradation of ecdysteroids, juvenile hormones, pheromones, and neurotransmitters [37, 38]. After the actions of P450s and CCEs followed by GSTs, the xenobiotics-derived polar compounds or conjugates can be transported out of the cell by ATP-binding cassette transporters (ABC transporters) [39]. In some cases, ABC transporters and others directly and efficiently transport xenobiotics out of the cell without enzymatic modifications to prevent the exertion of toxicity [39]. Since various natural and synthetic chemical compounds have been used to control honey bee mites, it is of considerable interest to understand how *T. mercedesae* may detoxify such miticides and develop resistance.

**Figure 4:**
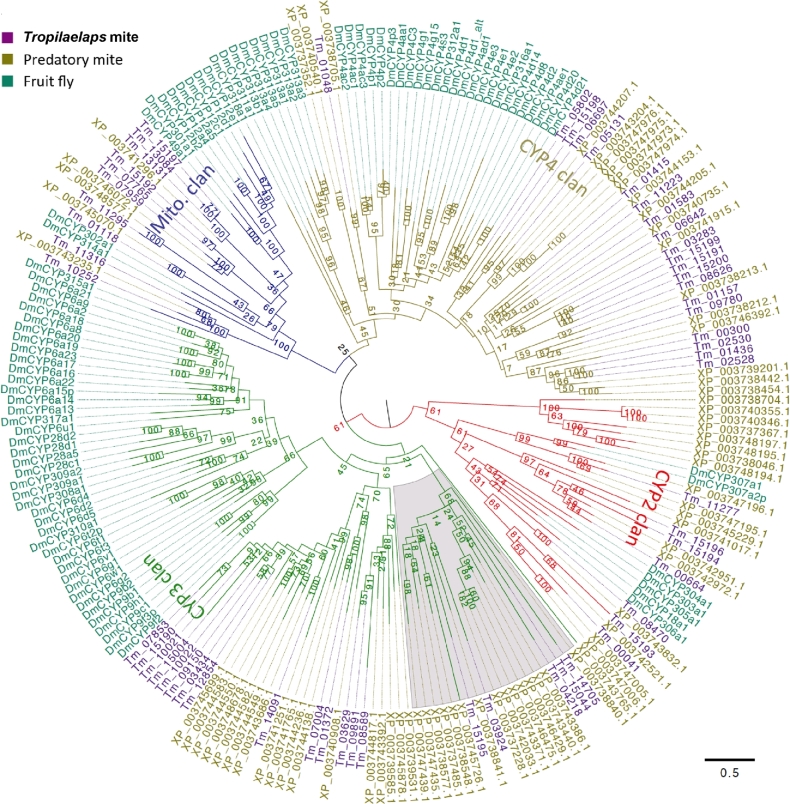
Phylogeny of *T. mercedesae*, *M. occidentalis*, and *D. melanogaster* cytochorme P450. The phylogenetic tree was constructed by maximum likelihood method and rooted at the middle point. P450s are clustered to CYP2, CYP3, CYP4, and mitochondrial clans are shown by red, green, blue, and dark yellow branches, respectively. *D. melanogaster* (DmCYP), *T. mercedesae*, and *M. occidentalis* P450s are indicated by dark green, purple, and dark yellow, respectively. *T. mercedesae* and *M. occidentalis* P450s are designated by protein IDs.

We manually annotated 56 *T. mercedesae* P450 (TmP450) genes in which 18 appeared to be pseudogenes. In fact, the expression of none of these genes was supported by RNA-seq data. Thus, *T. mercedesae* has only 38 apparently functional P450 genes similar to the human louse, *Pediculus humanus* [40], and the expression of 36 genes was confirmed by RNA-seq data (Supplementary Table S12). Similar to insect P450s, they are phylogenetically clustered into CYP2, CYP3, CYP4, and mitochondrial clans (Fig. 4). The classification was based on *D. melanogaster* P450s, but only three TmP450 genes (Tm11277, Tm11316, and Tm10252) have *D. melanogaster* P450 (DmP450) orthologs classified as CYP2 and mitochondrial clans (Fig. 4; Table 3). Thus, only a few P450 genes were present in the last common ancestor of arthropods and might be associated with the synthesis and degradation of hormones. In the two large CYP3 and CYP4 clans, DmP450s and the mite P450s are phylogenetically separated, suggesting that they have independently expanded after the split of the ancestors of mites and insects (Fig. 4). All of the TmP450 genes have orthologs in the *M. occidentalis* genome as recently reported [41], but *M. occidentalis* has 12 and 13 more genes than *T. mercedesae* in the CYP2 and CYP3 clans, respectively, by our analysis (Table 3)*. T. mercedesae* appears to have lost the CYP3 clan members from the common ancestor of the Parasitiformes (Fig. 4) as suggested by CAFE analysis (Supplementary Table S13). Some of the TmP450 genes are differentially expressed between nymph, adult male, and adult female (Supplementary Fig. S12; Supplementary Table S14), suggesting that they would be involved in the synthesis and degradation of hormones to control molting and sex-specific specific phenotypes of *T. mercedesae.*

**Table 3:** Comparison of the number of CYP2, 3, 4, and mitochondrial clan members in Insecta, Crustacea, and Acari.

	Total	CYP2	CYP3	CYP4	Mitochondria
**Insecta**					
*D. melanogaster*	88	7	11	32	36
*A. gambiae*	105	10	9	46	40
*A. aegypti*	160	12	9	57	82
*B. mori*	85	7	12	36	30
*A. mellifera*	46	8	6	4	28
*N. vitripennis*	92	7	7	30	48
*T. castaneum*	134	8	9	45	72
*A. pisum*	64	10	8	23	23
*P. humanus*	36	8	8	9	11
**Crustacea**					
*D. pulex*	75	20	6	37	12
**Acari**					
*T. mercedesae*	56	7	19	20	10
*M. occidentalis*	75 (63)	19 (16)	32 (23)	19	5
*T. urticae*	86	48	5	23	10

The data of four insects, *Anopheles gambiae*, *Aedes aegypti*, *Nasonia vitripennis*, and *Tribolium castaneum* are also included. Data refer to references [103] and this study. The numbers in parentheses are derived from previous report [41].

*T. mercedesae* has 15 GST genes (TmGST) in which 8 appear to be pseudogenes without evidence of the mRNA expression in the transcriptomes. This leads to only seven functional TmGST genes with mRNA expression confirmed by RNA-seq data (Supplementary Table S15). According to the reference data sets (*D. melanogaster* and *T. urticae* GSTs), the phylogenetic analysis of TmGSTs revealed the presence of four subfamilies (delta, mu, omega, and kappa) and an unclassified TmGST gene (Supplementary Fig. S13). Members in the mu, delta, epsilon, omega, theta, and zeta GST subclasses have been reported to function in a wide range of detoxification [42]. Epsilon, sigma, theta, and zeta subfamilies are absent in both *T. mercedesae* and *M. occidentalis* by our analysis in contrast to the recent report [41]; however, *I. scapularis* contains epsilon and zeta subfamilies and *T. urticae* has the theta subfamily (Supplementary Table S16). This suggests that these three subfamilies have been lost from the *T. mercedesae* and *M. occidentalis* genomes. The full-length orthologs of the five TmGST pseudogenes (Tm_05455, Tm_09167, Tm_15202, Tm_15203, and Tm_15206) are present in *M. occidentalis* (Supplementary Fig. S13), suggesting that the delta and mu GST subfamilies have undergone constriction in *T. mercedesae*.

Insect CCEs can be divided into 14 subfamilies (A to N) with three major groups based on the functions of dietary detoxification (A–C), hormone and pheromone degradation (D–H), and neurotransmitter degradation (I–N) [43]. We manually annotated 50 *T. mercedesae* CCE genes, in which 8 appeared to be pseudogenes without mRNA expression (Supplementary Table S17). The number of functional CCE genes in *T. mercedesae* is thus comparable to that in *M. occidentalis* [41] (Supplementary Table S18). Intriguingly, there are no mite CCEs in the subfamilies AF, H, I, K, and N; however, a massive mite-specific expansion is found in the subfamilies J and M by our analysis (Supplementary Fig. S14; Supplementary Table S18). Only three TmCCE genes (Tm_00126, Tm_05721, and Tm_08305) have *D. melanogaster* orthologs, suggesting that CCE genes have independently duplicated in insects and mites. The expression of some TmCCE genes is biased between the nymph, adult female, and adult male (Supplementary Table S19). Above results demonstrate that *T. mercedesae* contains P450s, GSTs, and CCEs, although the number and composition of subfamilies are different from those of other arthropods. Some of these enzymes may engage in detoxifying miticides and other xenobiotics in *T. mercedesae.*

We annotated 54 ABC transporter genes in the *T. mercedesae* genome, and the expression of 47 genes was confirmed by RNA-seq data (Supplementary Table S20). Similarly, *M. occidentalis* contains 57 ABC transporters that are comparable to those present in *D. melanogaster* (56 genes) (Supplementary Table S20). However, mite-specific expansion is found in the ABCC subfamily, and instead fruit fly-specific expansion is observed in the ABCG subfamily (Supplementary Fig. S15). The ABCC subfamily includes many vertebrate multidrug-resistance associated proteins that extrude drugs with broad specificity [39]; thus, the expanded ABCC subfamily members in *T. mercedesae* could be involved in conferring resistance against various miticides. In the fruit fly, expansion has been observed of the ABCG subfamily, which contains the transporters for the uptake of pigment precursors into the cells of the Malpighian tubules and developing compound eyes (Supplementary Fig. S15). Because these mites do not have eyes, fewer numbers of the ABCG transporters would be sufficient. The mites and fruit fly appear to have independently expanded ABCA subfamily members (Supplementary Fig. S15). These results suggest that most of the ABCA and ABCC transporters may carry out different functions in mites and fruit flies. Interestingly, two transporters, Tm_07059 and Tm_14842, form an independent clade separated from eight previously known ABC transporter subfamilies. In cases where the mite ABC transporter genes show biased expression between female, male, and nymph, most of them are highly expressed in either male or nymph compared to female (Supplementary Table S21).

### Sex determination genes in *T. mercedesae*

Arthropods are known to use various strategies for sex determination [44]. In contrast to *T. mercedesae*, which is likely to use haplodiploidy, *M. occidentalis* employs parahaploidy, in which the functional elimination of paternal chromosomes occurs during early embryogenesis resulting in male development [45, 21]. To gain insight into the mechanism of sex determination of *T. mercedesae*, we manually annotated the candidate genes for sex determination in the *T. mercedesae* genome. Similarly to *M. occidentalis* [21], *T. mercedesae* does not contain upstream sex determination genes (*Sex-lethal* and *transformer*) but has the homologs of downstream sex determination genes, *transformer-2*, *dmrt* (doublesex and mab3 related transcription factor), and *intersex. T. mercedesae* has the most *dmrt* genes of the arthropods that we tested (Supplementary Table S22) and has two extra *dsx* genes compared to *M. occidentalis* (Supplementary Fig. S16). The Dmrt93B ortholog is present in *T. mercedesae* (Tm_07872) but not in *M. occidentalis* (Supplementary Fig. S16), and all of the *dmrt* genes are highly expressed in the male (Supplementary Fig. S17). These results suggest that *T. mercedesae* and *M. occidentalis* may use a different set of genes for sex determination.

### Comparison of gene expression profiles between nymphs and adult males and females

**Figure 5:**
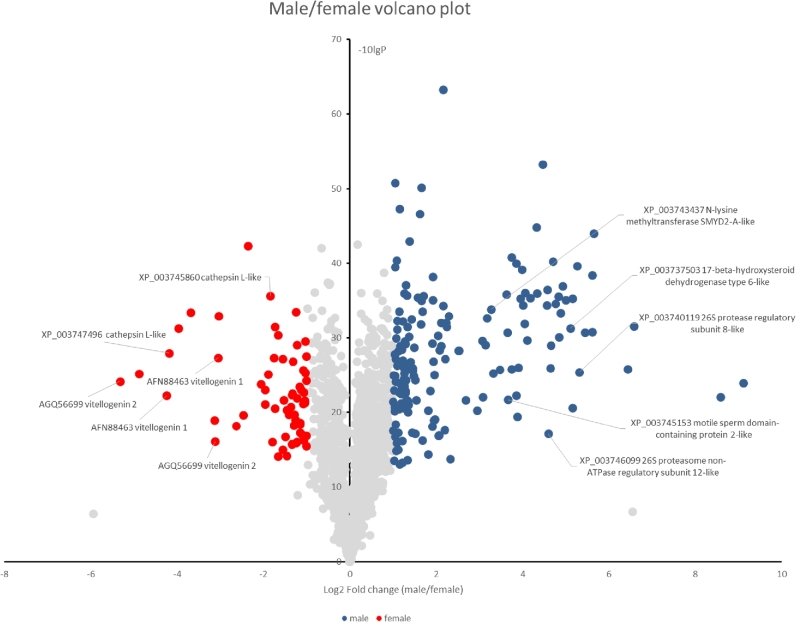
Volcano plot of proteins in the male and female mites. Proteins identified in the male and female mites by proteomic analysis are plotted according to the ratios of amounts present in male to female. Proteins abundant in the male and female are indicated by blue and red circles, respectively. Some of the representative proteins are indicated with the names and accession numbers of the best Blast hits.

Comparison between adult male and female transcriptomes and proteomes revealed that histone-lysine-*N*-methyltransferase gene family and N-acetyltransferase gcn5 gene family were highly expressed in the male compared to the female (Fig. 5; Supplementary File 1; and Supplementary Table S23), suggesting that the male mite may mostly depend on histone modifications for the epigenetic control of gene expression. This could be due to the ploidy compensation between males with haploid genomes and females with diploid genomes. At the protein level, males displayed overrepresentation of 26S proteasome subunits and a 17-beta-hydroxysteroid dehydrogenase (Fig. 5), which accords with the importance of the ubiquitin-proteasome system in sperm maturation [46] and a potential role for ecdysteriods in sexual maturation of *T. mercedesae* [47]. The female mite highly expresses the vitellogenin gene family and cathepsin L-like proteases (Fig. 5; Supplementary Table S23). This is consistent with active oogenesis in female mites, since both vitellogenin protein and Nanos mRNA would be deposited in the oocyte, while cathepsin L proteases may have a critical role in yolk processing as in *C. elegans* [48]. The results of the above transcriptome and proteome analyses are not identical, but a concordant set of 74 and 13 genes are up-regulated in the male and females, respectively. Comparison between adult female and nymph transcriptomes demonstrated that 46 of the 125 cuticle protein gene families, 13 of 24 chitin binding domain-containing protein gene families, and 9 of 16 chitinase gene families are expressed at a higher level in nymphs than in adult females (Supplementary Table S24), indicating that chitin metabolism as well as exoskeleton formation by molting is stimulated in the nymph. The nymph also highly expresses 18 of 29 protocadherin/fat gene families and 18 of 44 epidermal growth factor-related receptor gene families. These are likely to be involved in cell-cell adhesion and cell proliferation associated with the increase of cell number in nymph. Consistent with above results, GO analysis of genes highly expressed in nymphs compared to the adult females demonstrated that many GO terms related to cuticle formation and appendage morphogenesis are enriched (Supplementary Table S25).

### Symbiotic bacteria and infecting virus

**Figure 6:**
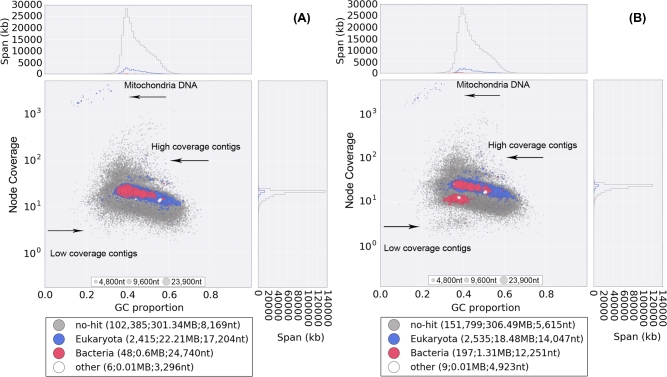
%GC-coverage plots of the preliminary assembled genomes of male (A) and female (B). Individual contigs are plotted based on their GC content (x-axis) and their node coverage (y-axis; logarithmic scale). Contigs are colored according to the taxonomic order of their best Megablast hit to the NCBI nt database (with E-value cut off < 1e−5). Contigs without the annotation are in gray.

Several bacteria have been shown to associate with mites and ticks [17, 49, 50]; however, bacteria associated with honey bee mites have not yet been fully investigated [11]. We thus attempted to identify any bacteria associated with *T. mercedesae* by filtering the bacteria-derived DNA contigs during the mite genome assembly. In the male and female GC%-coverage plots, some contigs were initially annotated as bacterial DNA in the major blue blob, and most of these were identified to contain *Wolbachia* sequences by BLASTN searches (Fig. 6). We confirmed that parts of *Wolbachia* genes are integrated into the mite genome by testing two genomic contigs using PCR with two sets of primers (one primer located in the mite gene, and the other in the *Wolbachia* gene) (Supplementary Fig. S18A and B). This phenomenon of nuclear *Wolbachia* transfers, or *nuwts*, has been observed widely in other arthropods and in nematodes [51], although to the best of our knowledge, this is the first report for a chelicerate. It suggests that *T. mercedesae* or the ancestor had *Wolbachia* as the endosymbiont in the past. Meanwhile, we extracted all reads mapped to the red blob (bacterial origin) in the female plot (Fig. 6) and reassembled them into 96 contigs. We annotated 751 protein-coding genes from the 81 contigs and found that 667 of these show high similarity to those of *Rickettsiella grylli* with an average identity of 79%. The rest of the 84 protein-coding genes showed similarity to 20 other bacteria species, such as *Diplorickettsia massiliensis* and *Legionella longbeachae*. This demonstrates that a close relative of *R. grylli* associates with female but not male *T. mercedesae. Rickettsiella* is an intracellular gamma-proteobacterium associated with a wide range of different arthropods without major pathogenicity to the host [52]. *Wolbachia* endosymbiont in the past may have been replaced by a species related to *R. grylli* in *T. mercedesae*. The potential effects on *T. mercedesae* as well as the potential for transmission to the honey bee remain to be determined. Since we did not find any DNA sequences of actinomycete species in our sequence reads, the two major ectoparasitic mites of honey bee (*V. destructor* and *T. mercedesae*) do not appear to share the same bacteria [11]. Nevertheless, both mites do not contain common arthropod gut bacteria, suggesting that they are not essential for the honey bee mites.

**Figure 7:**
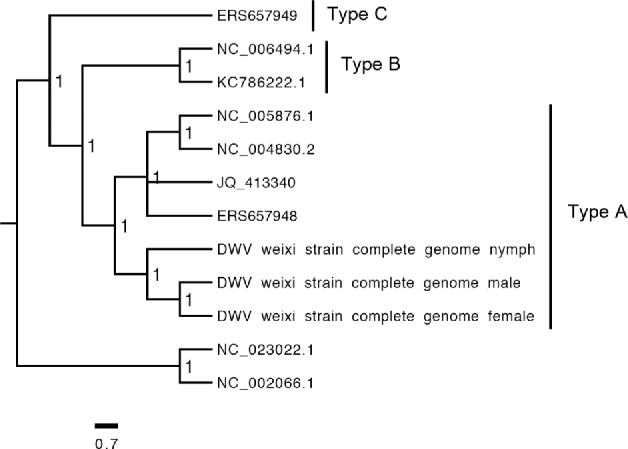
Classification of DWV in the *T. mercedesae* transcriptomes. The Bayesian phylogeny was constructed using Mrbayers based on the amino acid sequences of complete DWV genomes assembled from the adult males, adult females and nymphs transcriptomes (DWV weixi strain complete genome male, DWV weixi strain complete genome female, and DWV weixi strain complete genome nymph) as well as seven other DWV strains (type A variant: NC_005876.1, NC_004830.2, JQ_413340, and ERS657948; type B: KC_786222.1 and NC_006494.1; type C: ERS657949). The tree was rooted with Formica exsecta Virus 1 (NC_023022.1) and Sacbrood Virus (NC_002066.1).

We also assembled DWV RNA in the adult male and female, as well as nymph, transcriptomes (Supplementary Table S26). This is consistent with previous reports [53, 6, 7]; however, our data expand the infected stages to include the adult males and nymphs. DWV sequence reads represented one-third of the whole RNA-seq data, and these very high levels of DWV RNA were further confirmed by qRT-PCR (Supplementary Table S27). The proteomic analysis of females and males recovered many peptides derived from the capsid (structural) proteins, but very few peptides from the nonstructural proteins of DWV, demonstrating that the majority of DWV associated with the mites exists as mature virions (Supplementary Fig. S19). Similar observations were also reported for *V. destructor* [54]. We assembled three full-length DWV RNA genomes and found that they are phylogenetically clustered with type A DWV [55] (Fig. 7). Thus, *T. mercedesae* may spread the specific strain of DWV (type A in this study) to honey bees as suggested for *V. destructor* [56]. Considering that *T. mercedesae* was unlikely to carry DWV when associated with the original host, *A. dorsata*, DWV infection could impose a negative impact on the mite. It will be crucial to understand the nature of interactions between honey bee, mite, and DWV to measure the impact of *T. mercedesae* infestation on honey bee colonies. However, in contrast to *V. destructor*, we did not detect baculoviruses in either the genome or the transcriptome sequences [11].

*T. mercedesae* has a very specialized life history and habitat as an ectoparasitic mite strictly depending on honey bees in a colony with closed and stable environment. Thus, comparison of the genome and transcriptome sequences with those of a free-living mite and a tick has revealed the specific features of the genome shaped by interaction with the honey bee and colony environment. Our key findings are the following;

1. Amino acid substitutions have been accelerated within the conserved core genes of *T. mercedesae* and *M. occidentalis*
2. *T. mercedesae* has undergone the least gene family expansion and contraction between the seven arthropods we tested
3. The numbers of HSP70 family genes and sensory system genes are reduced
4. *T. mercedesae* may have evolved a specialized cuticle and water homeostasis mechanisms, as well as epigenetic control of gene expression for ploidy compensation between male and female
5. *T. mercedesae* contains all gene sets required to detoxify xenobiotics, enabling it to be miticide resistant
6. *T. mercedesae* is closely associated with a symbiotic bacterium (*Rickettsiella grylli*-like) and DWV, the most prevalent honey bee virus.

Manipulation of symbiotic *R. grylli-*like bacteria in the female mites may give the opportunity to control *T. mercedesae* in the future. Our *T. mercedesae* datasets, alongside published *V. destructor* genome and transcriptome sequences, not only provide insights into mite biology, but may also help to develop measures to control the most serious pests of the honey bee.

## Methods

### Mite sample collection

Based on the morphological and ethological characteristics [57], adult males and females as well as nymphs of *T. mercedesae* were identified and collected from a single honey bee colony for the flow cytometric analysis and Illumina sequencing (genome and transcriptome). Meanwhile, the adult females #2 sample (Supplementary Table S1) was collected from a different colony. Both colonies were obtained from a beekeeper in Jiangsu Province, China. The mites collected for genome sequencing and proteomic characterization were stored in acetone at 4°C until use. The mites used for RNA-seq were sorted at −80°C before the transport.

### Genome sequencing

Before DNA extraction, the mite bodies were carefully washed twice with acetone to remove any nontarget organisms that might adhere on the mite surface. Subsequently, a single male and a single female mite were air dried (15 min) and individually triturated in 180 μL of lysozyme buffer (1M Tris-HCl, 0.5M EDTA, 1.2% Triton X-100, and 0.02% lysozyme) with a tissuelyser II (Qiagen, Valencia, USA) using a 3-mm stainless steel bead at 25 000 motions/min for 30 sec. After incubating the samples at 37°C for 30 min, total DNA was extracted from each of the triturated samples with DNeasy Blood and Tissue kit (Qiagen) by following the manufacturer's spin-column protocol for animal tissue. To maximize the yield of DNA extraction, two successive elution steps, each with 50 μl elution buffer, were performed. The DNA concentrations were determined by spectrophotometry, a sensitive and commonly used fluorescent dye assay (Qubit® dsdna BR assay, Life Technologies Europe, Naerum, Denmark) according to the manufacturer's instructions. Two paired-end Illumina DNA libraries were constructed with the male and female total genomic DNA samples (30 ng each) using a Nextera DNA sample preparation kit (Illumina, Great Chesterford, UK). The DNA libraries were then quality controlled and sequenced with Illumina Hiseq 2500 system using two individual lanes in the Centre for Genomic Research at the University of Liverpool. The raw fastq files were trimmed to remove Illumina adapter sequences using Cutadapt (v1.2.1) [58]. The option “−O 3” was set, so the 3' end of any reads which matched the adapter sequence over at least 3 bp was trimmed off. The reads were further trimmed to remove low quality bases, using Sickle (v1.200) [59] with a minimum window quality score of 20. After trimming, reads shorter than 10 bp were removed.

### Transcriptome sequencing

Male, female, and nymph mites were shipped to BGI-Shenzhen with dry ice for total RNA extraction, polyA^+^ RNA enrichment, cDNA library preparation, and Illumina Hiseq 2000/4000 sequencing. Total RNA (Supplementary Table S1) was extracted from a pool of 20∼30 mites using Trizol reagent (Qiagen) and treated with DNase I (Qiagen). Next, polyA^+^RNA was isolated by magnetic beads with oligo (dT) and digested to short fragments by mixing with the fragmentation buffer, and then the cDNA was synthesized. The short DNA fragments were purified and resolved with EB buffer for end reparation and single nucleotide A (adenine) addition followed by ligation with adapters. DNA fragments suitable for sequencing were then selected for the PCR amplification. After QC steps, Illumina Hiseq 2000 system was used to sequence the libraries of adult males #1 (in two lanes), adult females #1 (in two lanes), nymphs #1 (in two lanes), and adult females #2 (in a single lane), whereas adult males #2 and nymphs #2 were sequenced with Illumina Hiseq 4000 system in a single lane. Raw reads were trimmed and filtered by internal tools of BGI-Shenzhen.

### Estimation of genome size and ploidy of *T. mercedesae*

Nuclear DNA contents of *T. mercedesae* males and females were estimated by a method of propidium iodide staining followed by flow cytometry [60]. Nuclei were isolated from ten *T. mercedesae* adult males and females, the heads of 10 *D. melanogaster* females (1C = 175Mb) [61], and the brain of a honey bee worker (1C = 262 Mb) [62] by homogenizing each sample with 1 ml of a cold Galbraith buffer (30 mM sodium citrate, 18 mM MOPS (3-morpholinopropanesulfonic acid), 21 mM MgCl_2_, 0.1% Triton X-100, 1 mg/L RNase A) using a loose pestle. The cellular debris were removed by filtering through 20-μm nylon mesh. Stained nuclei from adult male and female mites were independently analyzed with two reference standards using a BD FACS flow cytometer (BD Biosciences, San Jose, CA). Nuclear genome size was then calculated according to the following formula: Sample nuclear DNA content = (mean peak of sample/mean peak of reference standard) × nuclear DNA content of reference standard. We estimated the genome size by analyzing the frequency of *k*-mers counted by Jellyfish [63] with the following formula [64]: estimated genome size (bp) = total number of *k*-mer/the maximal frequency. The ploidy is the ratio of nuclear DNA content to genome size.

### De novo assembly of genomic DNA

Prior to assembly, we discarded all male and female sequencing reads aligned to honey bee genome sequence by Bowtie 2 (v2.2.1) [65]. The unaligned male and female reads were then extracted by bam2fastq (v1.1.0) and assembled individually by Velvet v1.2.07 [66] into preliminary contigs with their best k-mers and parameters of ‘-min_contig_lgth = 200 and -ins_length 1105 (male)/939 (female).’ DNA sequences derived from nontargets such as bacteria and mitochondria were filtered out based on the preliminary assemblies of male and female genome sequences using a GC-coverage (proportion of GC bases and node coverage) plot-based method by blobtools (v0.9.19) [67] (Fig. 6), resulting in a total of 400 520  654 and 453 725 764 “clean reads” for male and female mite, respectively. The male “clean reads” were reassembled and optimized up to scaffold level using the VelvetOptimiser (v2.2.5) with the velvet parameters set to ‘-min_contig_lgth 200 and -ins_length 1105.’

### Genome annotation

To find, classify, and mask repeated sequences in the assembled male genome, a de novo repeat library was first built using Repeatmodeler (A F.A. Smit and P. Green, unpublished data) with ‘-database’ function followed by Repeatmasker (A.F.A. Smit and P. Green, unpublished data) using default setting for de novo repeated sequences prediction. Then, a homology-based prediction of repeated sequences in the genome was achieved using Repeatmasker with default setting to search against RepBase repeat library issued on January 13, 2014. For noninterspersed repeated sequences, we ran Repeatmasker with the ‘-noint’ option, which is specific for simple repeats, micro satellites, and low-complexity repeats.

RNA-seq reads obtained from all samples were aligned to the masked genomic scaffolds to determine the exon-intron junctions using Tophat (v2.011) with default setting [68]. Cufflinks (v0.8.2) [69] used the spliced alignments with default setting to reconstruct 44 614 transcripts from which 12 298 transcripts with intact coding sequences were selected by a Perl script developed by Liu et al. [70]. Thee ab initio gene prediction programs, including Augustus (v3.0.3) [71], SNAP (v2013-11-29) [72], and Genemarker (v2.3e) [73] were used for de novo gene predictions. Augustus and SNAP were trained based on the selected intact coding sequences with default setting, whereas GeneMark [73] was self-trained with ‘–BP OFF’ option. We ran Augustus, SNAP, and Genemarker with default setting, and predicted 32 561, 67 258, and 79 928 gene models in the masked genomic scaffolds, respectively (Supplementary Table S2).

We also generated an integrated gene set using MAKER v2.31.4 [74] pipeline. The MAKER pipeline runs Augustus, SNAP^,^ and Genemarker to produce de novo gene predictions, and integrates them with the evidence based predictions. They were generated by aligning all Cufflinks assembled transcript sequences and the invertebrate RefSeq protein sequences (downloaded on May 17, 2014 from NCBI) to the masked male mite genome by BLASTN and BLASTX, respectively. The MAKER pipeline was run with ‘-RM_off’ option to turn all repeat masking options off, and all parameters in control files were left with their default settings.

Genes identified by de novo prediction, which did not overlap with any genes in the integrated gene sets, were also added to the final gene set if they showed significant hits (BLASTP E-value **<** 1e**-**5) to SwissProt proteins or could be annotated by Interproscan (v4.8) [75] with InterPro superfamily database (v43.1) using ‘-appl superfamily -nocrc’ options.

### ncRNA annotation

In this analysis, we annotated four types of ncRNA: transfer RNA (tRNA), ribosomal RNA (rRNA), microRNA, and small nuclear RNA (snRNA). Genes encoding tRNA were predicted by trnascan-SE (v1.3.1) [76] with eukaryote parameters, and rRNA genes were identified by aligning the rRNA template sequences from invertebrates (database: SILVA 119) to the *T. mercedesae* genomic DNA using BLASTN with an E-value cutoff of 1e-5. Genes encoding miRNA and snRNA were inferred by the Infernal software (v1.1.1) [77] using release 12 of the Rfam database with ‘–cut_tc’ option.

### Protein functional annotation

We performed the initial and principal domain annotation with the Pfam database (release 27) using the hmmscan in HMMER v3.1b1 with default settings. Additional domains (superfamily, Gene3d, Tigrfams, Smart, Prosite, and Prints domain models) and domain/motif based GO term were assigned using InterProScan search against InterPro database (v43.1) with ‘-cli -nocrc -goterms -iprlookup’ options.

We used Blast2GO pipeline (v2.5) [78] to further annotate proteins by Gene Ontology (GO) terms. In the first step, we searched the nr database with BLASTP using a total of 15,190 protein sequences as queries. The E-value cutoff was set at 1e-6 and the best 20 hits were collected for annotation. Based on the BLAST results, Blast2GO pipeline then predicted the functions of proteins to assign GO terms, and merged the InterProScan deduced domain/motif based GO terms into these BLAST-based annotations.

The metabolic pathway was constructed based on the KAAS (KEGG Automatic Annotation Server) online server [79] using the recommended eukaryote sets, all other available insects, and *I. scapularis*. The pathways in which each gene product might be involved were derived from the best KO hit with BBH (bi-directional best hit) method.

### GO enrichment

We performed the GO enrichment analyses of gene sets with Fisher's exact test embedded in the Blast2GO desktop version (v2.8). If not specifically stated, the *P* values were corrected according to the critical FDR. The enrichments were tested by comparing the GO terms with the pooled set of GO terms of all *T. mercedesae* proteins.

### Protein data sets of reference genomes

Protein data sets of the following arthropod genomes were used as references: *D. melanogaster* (fruit fly; GOS release: 6.03) [80], *A. mellifera* (honey bee; GOS release: 3.2) [62], *T. urticae* (spider mite; GOS release: 20140320) [15], *Stegodyphus mimosarum* (velvet spider; GOS release: 1.0) [81], *I. scapularis* (black-legged tick; GOS release: 1.4; GenBank project accession: ABJB010000000) [19], and *M. occidentalis* (predatory mite; GOS release: 1.0) [21]. *Caenorhabditis elegans* (nematode; GOS release: WS239) [82] was used as the outgroup. Domain, GO, and KEGG annotation of proteins in the reference species (if required) was conducted using the same methods as those used for *T. mercedesae*.

### Gene family phylogenetics

We first aligned orthologous protein sequences with Mafft (v7.012b) [83] or Kalign (v2.0) [84] and then used Gblocks (v0.91b) [85] to automatically eliminate the divergent regions or gaps prior to phylogenetic analysis. However, we manually trimmed the aligned sequences for big gene sets. The best substitution models of amino acid substitution were determined for the alignments by Prottest (v3.4) with parameters set to “-all-matrices, -all-distributions, -AIC” [86]. Then, phylogenetic trees were constructed using maximum likelihood methods (Phyml, v3.1) [87] or Bayesian methods (MrBayes, v3.2.3) [88]. In addition, a neighbor-joining method was also used for building the distance-based trees using MEGA (v6.06) [89].

### Species tree phylogenetics

Since the rapid evolution of acariform mites may challenge phylogenetic analyses due to long-branch attraction [90], we used a very strict E-value (1e-50) when performing a reciprocal BLASTP to gate out the most variant orthologous genes across all genomes tested. The reciprocal BLAST search resulted in identification of a total of 926 highly conserved one-to-one orthologs in all 8 genomes. Each of these orthologous groups was aligned using Mafft in “-auto” option. These alignments were trimmed by Gblocks and concatenated into the unique protein superalignments. ProtTest determined the best-fit substitution model of LG with invariant sites (0.109) and gamma (0.913) distributed rates using parameters as above before conducting the phylogenetic analysis with Phyml.

### Analysis of gene family expansion and positive selection

Orthologous gene families between *T. mercedesae* and six reference arthropods were defined based on OrthoMCL (v1.4) [91] clustering. We used CAFE (v3.1) [92] to infer the gene family expansion and contraction in *T. mercedesae* against all reference arthropods or against Parasitiformes (*I. scapularis* and *M. occidentalis*). The ultrametric species tree used in CAFE analyses was created as described in Gene family phylogenetics section.

We also calculated ω (dN/dS) ratios for 1865 one-to-one orthologs defined by OrthoMCL using codeml in the PAML package with the free-ratio model. Branches with ω > 1 are considered under positive selection. The null model used for branch test was the one-ratio model (nssites = 0; model = 0), where ω was the same for all branches. Kappa and omega values were automatically estimated from the data, where the clock was set to be entirely free to change among branches. *P* value was determined twice using the log-likelihood difference between the two models compared to χ2 distribution with the difference in number of parameters between one-ratio and free-ratio models. To estimate significance with the *P* value, likelihood-ratio test was used to compare lnl values for each model and test if they were significantly different. The differences in log-likelihood values between two models were compared to chi-square distribution with degree of freedom equal to the difference in the number of parameters for two models. Measurement of dS was assessed for substitution saturation, and only dS values < 3.0 were maintained in the analysis for positive selection. Genes with high (ω >10) were also discarded.

### De novo transcriptome assembly and estimation of the transcript abundance

All RNA-seq reads mapped to the honey bee transcripts were filtered out first. Then, all RNA-seq samples in Supplementary Table S1 were individually de novo assembled by Trinity (v20131110) [93] with default setting. We used a RSEM [94] software package to estimate the expression levels (abundance) of de novo assembled transcripts and isoforms with default setting.

### Analysis of RNA-seq data

After further removing the RNA-seq reads corresponding to DWV sequence, we aligned the cleaned reads to the assembled *T. mercedesae* genome using Tophat with default setting. Then, Htseq-count in the Htseq Python package (v0.6.1) [95] was used to obtain raw read counts, with the default union-counting mode and option ‘-a’ to specify the minimum score for the alignment quality. The raw read count for each sample was then subject to further differential expression analysis using the EdgeR (v3.0) Bioconductor package [96]. We excluded mRNAs without at least one count per million in the replicates (low overall sum of counts) from the analyses as previously suggested [97]. We then normalized the library sizes of all samples according to the trimmed mean of M-values method, and dispersion was estimated from the replicates using the quantile-adjusted conditional maximum likelihood method. Pairwise comparisons of differential gene expression between the RNA-seq samples were performed using the function of Exact test. We used the corrected FDR *P*-value < 0.01, and logFC > 1 and logFC < 1 cut-offs for significance.

### qRT-PCR

We carried out qRT-PCR reactions, each in triplicate, using an Applied Biosystems 7500 Fast Real-Time PCR System and 2X KAPA SYBR FAST qPCR Master Mix (KAPA Biosystems Woburn, MA). To perform the absolute quantification of DWV RNA, we first prepared standard curves for DNA corresponding to DWV target RNA. The target DNA was prepared by PCR followed by the gel extraction. The DNA concentration was measured using Nanodrop 2000 spectrophotometer (Thermo Scientific) to calculate the original copy number by a formula, copy number = DNA concentration (ng/μl) × 6.02 × 10^23^ (copies/mol) / length (bp) × 6.6 × 10^11^ (ng/mol), in which 6.6 × 10^11^ ng/mol is the average molecular mass of one base pair, and 6.022 × 10^23^ copies/mol is the Avogadro's number. Linear standard curves were then generated using target DNA of 10^5^–10^9^ copy number per reaction followed by plotting the Ct values against log values of the copy number. After reverse transcription, the copy number of target RNA in a sample was estimated using the standard curve above. To carry out the relative quantification, we compared the relative expression levels of the target mRNA to *Ef-1α* mRNA as the internal reference using the 2^−ΔΔCt^ method. All primers used for qRT-PCR are listed in Supplementary Table S28.

### Proteomic analysis

Pools of male or female mites were lysed by sonication in 0.1% (w/v) Rapigest (Waters MS technologies) in 50 mM ammonium bicarbonate. Samples were heated at 80°C for 10 min, reduced with 3 mM DTT at 60°C for 10 min, cooled, then alkylated with 9 mM iodoacetamide (Sigma) for 30 min (room temperature) protected from light; all steps were performed with intermittent vortex-mixing. Proteomic-grade trypsin (Sigma) was added at a protein:trypsin ratio of 50:1 and incubated at 37°C overnight. Rapigest was removed by adding TFA to a final concentration of 1% (v/v) and incubating at 37°C for 2 hours. Peptide samples were centrifuged at 12,000 × g for 60 min (4°C) to remove precipitated Rapigest. The peptide supernatant was desalted using C_18_ reverse-phase stage tips (Thermo Scientific) according to the manufacturer's instructions. Samples were desalted and reduced to dryness as above and re-suspended in 3% (v/v) acetonitrile, 0.1% (v/v) TFA for analysis by MS.

Peptides were analysed by on-line nanoflow LC using the Ultimate 3000 nano system (Dionex/Thermo Fisher Scientific) coupled with a Q-Exactive mass spectrometer (Thermo Fisher Scientific). Samples were loaded on a Nano-Trap column (Acclaim^®^ PepMap 100, 2 cm × 75 μm , C_18_, 3 μm, 100 Å) then eluted in line with the analytical column (Easy-Spray PepMap^®^ RSLC , 50 cm × 75 μm, packed with 2 μm C_18_, 100 Å particles), fused to a silica nano-electrospray emitter (Dionex). The column was operated at a constant temperature of 35°C. Chromatography was performed with a buffer system consisting of 0.1% formic acid (buffer A) and 80% acetonitrile in 0.1% formic acid (buffer B). The peptides were separated by a linear gradient of 3.8% to 50% buffer B over 90 minutes at a flow rate of 300 nl/min. The Q-Exactive was operated in data-dependent mode with survey scans acquired at a resolution of 70 000. Up to the top 10 most abundant isotope patterns with charge states +2, +3, and/or +4 from the survey scan were selected with an isolation window of 2.0Th and fragmented by higher energy collisional dissociation with normalized collision energies of 30. The maximum ion injection times for the survey scan and the MS/MS scans were 250 and 50 ms, respectively, and the ion target value was set to 1E6 for survey scans and 1E5 for the MS/MS scans. Repetitive sequencing of peptides was minimized through dynamic exclusion of the sequenced peptides for 20s.

Thermo RAW files were imported into Progenesis LC–MS (version 4.1, Nonlinear Dynamics). Runs were time aligned using default settings and using an auto selected run as reference. Peaks were picked by the software using default settings and filtered to include only peaks with a charge state between +2 and +7. Spectral data were converted into .mgf files with Progenesis LC–MS and exported for peptide identification using the Mascot (version 2.3.02, Matrix Science) search engine. Tandem MS data were searched against translated ORFs from *T. mercedesae*, *Apis mellifera* (OGSv3.2) [98], and DWV (Uniprot 08 2016) (total; 30 666 sequences; 12 194 618 residues). The search parameters were as follows: precursor mass tolerance was set to 10 ppm and fragment mass tolerance was set as 0.01Da. Two missed tryptic cleavages were permitted. Carbamidomethylation (cysteine) was set as a fixed modification and oxidation (methionine) set as variable modification. Mascot search results were further validated using the machine learning algorithm Percolator embedded within Mascot. The Mascot decoy database function was utilized and the false discovery rate was < 1%, while individual percolator ion scores >13 indicated identity or extensive homology (*P* < 0.05). Mascot search results were imported into Progenesis LC–MS as XML files. Peptide intensities were normalized against the reference run by Progenesis LC-MS and these intensities are used to highlight relative differences in protein expression between samples.

## Data availability

All sequence data we obtained and analyzed are deposited under the project accession number PRJNA343868 in NCBI. Proteomics data have been deposited to the ProteomeXchange Consortium via the PRIDE [99] partner repository with the dataset identifier PXD004997. Additional supporting data is also available via the *GigaScience* GigaDB repository [100].

### Abbreviations

ABC: transporter ATP-binding cassette transporter; CCE: carboxylesterase; CSP: chemosensory protein; CYP: cytochrome P450; DWV: Deformed Wing Virus; GO: Gene Ontology; GR: gustatory receptor; GST: glutathione-S-transferase; IR: ionotropic receptor; MS: mass spectrometry; OBP: odorant binding protein; OR: olfactory receptor; P450: cytochrome P450

## Supplementary data

Supplementary data are available at *GIGSCI* online.

### Competing interests

We declare no competing interests.

### Author contributions

XD conducted all experiments except the proteomic analyses, which were carried out by SDA and DX. TK, ACD, and BLM planned and supervised the research. XD and TK wrote the manuscript, which was revised by ACD and BLM.

## Supplementary Material

Supplemental material Supplementary data are available at *GIGSCI* online.

GIGA-D-16-00107_Original_Submission.pdf

GIGA-D-16-00107_Revision_1.pdf

GIGA-D-16-00107_Revision_2.pdf

Response_to_reviewer_comments_Original_Submission.pdf

Response_to_Reviewer_Comments_Revision_1.pdf

Reviewer_1_Report_(Original_Submission).pdf

Reviewer_1_Report_(Revision_1).pdf

Reviewer_2_Report_(Original_Submission).pdf

Reviewer_2_Report_(Revision_1).pdf

Reviewer_3_Report_(Original_Submission).pdf

Reviewer_3_Report_(Revision_1).pdf
