# Supplementary material for: Draft genome of the honey bee ectoparasitic mite, Tropilaelaps mercedesae, is shaped by the parasitic life history
Source: Gigascience. 2017 Feb 22;6(3):1–17. doi: 10.1093/gigascience/gix008 (PMC5467014; doi:10.1093/gigascience/gix008)
Supplement: Response_to_reviewer_comments_Original_Submission.pdf [file gix008_Response_to_reviewer_comments_Original_Submission.pdf]

Reviewer #1: The authors describe the draft genome of the honey bee mite Tropilaelaps mercedesae. The sequencing statistics are acceptable. They also performed proteomics and RNA-seq to validate transcripts, used flow cytometry to estimate genome size and performed comparative analysis with other arachnid genomes. This study is worthwhile and contribute significantly to existing mite genomics. I therefore recommend acceptance once some issues have been addressed.

1. Page 3, line 88, 92: Will it be possible to include the statistics of other arachnid genomes in Table 1?

Authors: We have included the statistics data of nine other arachnid genomes in Table 1 of the revised manuscript as suggested.

2. Page 4, line 100: Can the coverage range or percentage of reads that constitute repetitive elements be indicated in the text?

Authors: The putative repetitive sequences represent 48.57% of total clean reads. This has been mentioned in the revised manuscript (Lines 104-107).

3. Page 4, line 131: The study by Hoy et al. (2016) indicated two different divergence dates depending on different hypotheses as proposed by Jeyaprakash and Hoy (2009). The first assumes that the Acari are monophyletic and for this a date of 336+-26 MYA was proposed for the Parasitiformes and 395+-24 for their divergence from the Acariformes. The second assumes that the Acari are not monophyletic and that the Parasitiformes then diverged from other arachnids (spiders) 459+-18 MYA. The current study states that parasitiformes diverged from other Arachnida 302 MYA, which is not similar to Hoy et al. (2016). The current study is based on a fossil calibration of 311-501 based on the oldest spider fossil. The node is younger than this estimate which indicates an underestimation in the molecular clock analysis. This date would also only hold if spiders and parasitiformes are truly sister genera. However, this relationship is not robust, since spiders and acariformes are the only other arachnids included in the tree. If for example, scorpions are closer to ticks than spiders, the minimum divergence date would have been ~428 MYA. Because no other arachnids were included, this dating is biased towards a younger divergence date. The analysis has to be expanded to other arachnids or this part of the study needs to be discarded. The most that can be said with some confidence at this point, is that the data support other studies that indicated the paraphyly of the the Acari.

Authors: We agree with the point raised above. Since we do not have enough data to support the divergence time, we have deleted the sentence "Based on this phylogenetic topology, we estimated that parasitiform mites and ticks separated from other arachnids approximately 302 Mya as recently reported [16] (Supplementary Fig. 5)." and Supplementary Figure 5 in the revised manuscript.

4. Page 4, line 136: Are these 15506 unique gene families or orthologous groups? Note that different orthologs can have related paralogs in the same genome that have the same protein folds and descend from common ancestors, and therefore belong to the same gene/protein family.

Authors: These are 15,506 orthology clusters defined by orthoMCL, and thus each cluster may contain both orthologs and paralogs (Lines 139-140 of the revised manuscript).

5. Page 4, line 140: Are these unclustered genes unique (distinct protein families/singletons), or are they paralogs of orthologs and therefore recent gene duplicates/lineage specific expansions? Their GO annotation suggest that these are recent duplicates. Are there any unique/singleton genes present that cannot be classified?

Authors: These unclustered genes which were not classified to any orthology cluster by orthoMCL must contain both T. mercedesae-unique genes and paralogs which are sufficiently divergent from their orthologs so that their sequence similarity was not recognized by orthoMCL. We found that 1,981 out of 5,846 unclustered genes are assigned with at least one GO term, suggesting that some of them are likely to be

such T. mercedesae paralogs. By using orthoMCL, it is difficult to distinguish between T. mercedesae-unique genes and the divergent paralogs. These have been described at Lines 144-150 in the revised manuscript.

6. Although lengthy, I enjoyed the comparative analysis part of the study and which address good issues in mite biology that will be useful to all researchers in this field.

7. Figure 1: Arthropod specific…., Species specific…

Authors: We have corrected the spelling in Figure 1 of the revised manuscript.

Reviewer #2: Dr. Kadowaki et al.,

The work described in your manuscript comprises an interesting system investigated using appropriate methods. The conclusions are appropriate, if not conservative. I found the manuscript to be well-written, but a few issues need to be addressed.

In general, the methods section requires far more detail. The subsections appear to have been written by several people and differ greatly in the amount of detail given. For example, specifics concerning the genome/transcriptome sequencing are lacking (how many samples were sequenced; how many lanes; how was the RNA extracted and prepped?), VELVET parameters for genome assembly, and MAKER parameters are all missing. Additionally, in some subsections, software versions are not indicated.

Authors: We have described the methods in more detail in the revised manuscript as suggested. We have added information on the number of samples sequenced, the samples and methods for RNA extraction, and the methods for genome/transcriptome sequencing. The detailed parameters for running VELVET and MARKER as well as the versions of software used for analyses have also been described in the Methods section (Lines 420-611).

Some subsections should be combined or rearranged. "Construction of phylogenetic trees" and "evolutionary analyses" have a lot of overlap and are somewhat confusing as currently written. I would recommend substituting/reorganizing them with/as "Species tree phylogenetics and divergence time estimations", "Gene family expansion and positive selection tests", and "Gene family phylogenetics".

Authors: We have added subsections "Species tree phylogenetics", "Gene family phylogenetics", and "Analysis of gene family expansion and positive selection" in the revised manuscript as suggested (Lines 548-556, 565-573, and 574-593). Furthermore, new subsections "Transcriptome sequencing", "Protein data sets of reference genomes", and "De novo transcriptome assembly and estimation of the transcript abundance" have also been added in the revised manuscript (Lines 450-462, 557-564, and 594-598).

The RNA-seq methods lack details concerning basic experimental design, replication, and quality control.

Authors: We have explained the details of RNA-seq methods in the subsection "Transcriptome sequencing" of the revised manuscript (Lines 450-462). We sequenced RNA isolated from a pool of adult males, adult females, or nymphs (immature females) which can be clearly distinguished by their morphology. Each pool contained 20-30 mites and the sequencing was carried out with two biological replicates for each sample as shown in Supplementary Table 1.

The proteomics subsection contains a more appropriate amount information than the rest of the methods, which are variable themselves.

Lastly, the reference format needs to be standardized.

Authors: The references have been formatted by BMC journal style in the revised manuscript (Lines 702-969).

Again, this work is well done and important. However, one of the tenants of Gigascience is reproducibility, so this current draft needs a more attention paid to methods in almost all regards.

Authors: Please refer to our comments above.

Reviewer #3: Dear Authors

Congratulations on your research article on the draft genome of a parasite of honey bees. Your study lays out the importance of understanding this organism, both from the specific point of view of helping honey bees, but also the general field of parasite genomics. Overall, this is a high quality genome paper with best practice methods for contamination checks, assembly, annotation, functional, and comparative genomics. Although the paper is well written, I feel it could do with some very light editing in a few sections that I have tried to highlight below.

Some specific comments (I am using the original line numbers which go from 1-941, rather than the 1-65 line numbers on each page that seem to have been added by the submission software). They are quite minor and should be easy to address before this manuscript is accepted and published.

51: Varroa destructor seems to be a non sequitur here when it hasn't been mentioned in the title or up to this point. I would suggest introducing it with a phrase.

Authors: We have added "another globally prevalent ectoparasitic mite of honey bee" following Varroa destructor with parenthesis in the revised manuscript (Lines 51-52).

57: Background - needs light editing. The emphasis seems to now be on V destructor rather than T mercedesae (which is in the title).

Authors: This is because V. destructor has been studied more extensively than T. mercedesae. Nevertheless, we have added more description on T. mercedesae in the revised manuscript (Lines 66-72).

75: change 'was' to 'were'

84: Suggest changing "Each of dual indexed paired-end DNA library was" to "Dual indexed paired-end DNA libraries were"

95: Suggest changing sequencing depth to k-mer depth.

Authors: We have corrected all of above points in the revised manuscript (Lines 80, 89, and 100).

96: Just a comment - I would emphasise that because 94% of the sequencing reads map back to the 353 Mb asssembly, you have greater evidence that the 'collapsing' of the assembly was because of repetitive sequences. I am a bit surprised however at this discrepancy in kmer based sizing and genome assembly sizing. Could it be that you used very different k-mers in the genome size estimation and in the final assembly? Alternatively, the assembler may have had aggressive settings for bubble popping. I tried to look for the velvet settings used in the Methods section but couldn't find them - apologies if I missed them elsewhere. Please include these.

Authors: As described in subsection "De novo assembly of genomic DNA" in the Methods section of the revised manuscript, we set the parameter of Velvet to assemble the genome as '-min_contig_lgth=200', and thus the contigs < 200 bp were filtered out from the assembly. As a result, we found that 95.33% of the reads actually used for the genome assembly by Velvet could be mapped back to 353 Mb assembled mite genome. Meanwhile, we also found that repetitive sequences represented 48.57% of total clean reads (Supplementary Table 4) and the majority of them were found in the high-coverage regions of the genome

(Supplementary Table 5). These have been described at Lines 93-94 and 104-107 in the revised manuscript. We also note that the assembled genome sequence of Ixodes scapularis represents only 57% of the total genome size due to a large number of repetitive DNA sequences.

121: Comment and disclaimer: I don't know much about arthropod phylogenetics, but the methods/conclusions described here seem valid.

135: Comment: OrthoMCL seems to be the most commonly used software for this type of analysis, but I hope more studies will use OrthoFinder in the future as it is more sensitive and specific in my (limited) experience.

Authors: Although we do not have experiences using OrthoFinder, this would be a good substitute for OrthoMCL as suggested above.

121-165: Comment: A really well described comparative genomics section.

166-228: Comment and disclaimer: I don't know much about sensory systems but as a non-expert I was able to follow the methods and results, and I agree with the results, tables, figures and conclusions.

194: Suggest rephrasing : "Without orthology" seems odd when the figure shows Dm and Tm proteins in the same tree.

Authors: As shown in Fig. 3, none of five T. mercedesae GRs forms a clade with D. melanogaster GRs, demonstrating that there are no orthologous GRs between T. mercedesae and D. melanogaster. We have described this at Lines 201-203 in the revised manuscript.

229-344: Same comment and disclaimer as above.

345: Glad to see TAGC plots used here (I think all genome sequencing projects should do them, or something like them, eg CONCOCT, Anvi'o by default). However, if it is not too much trouble (as you probably have the coverage and seq similarity hit files already), could I request you to use the updated blobtools suite at https://github.com/DRL/blobtools ? The plots are easier to interpret and provide much more information on the span/number of contigs in each blob. It will also help visualise the high repeat content. It is quite quick to run diamond blastx against uniref90 to colour the blobs better so that fewer contigs are left unannotated.

Authors: As suggested, we ran the updated blobtools and have replaced previous Figure 5 with Figure 6 containing the new results in the revised manuscript. We agree we can interpret the data more easily and thank the reviewer to suggest us this updated version.

345-369: Can you summarise the findings here better? I think what this section is saying is that there is possibly a cobiont/symbiont/endosymbiont (more simiar to Rickettsiella grylli rather than Wolbachia) - how do you know it is not a contaminant? A blobtools plot with identification at the level of clade would be really helpful in resolving this. In addition, there are Nuwts that are integrated into the genomic DNA so this mite may have had a Wolbachia endosymbiont in the past. Is that the conclusion?

Authors: We found both male and female mite genomes contain the remnants of Wolbachia genes which were confirmed by genomic PCR as shown in Supplementary Fig. 18 of the revised manuscript. We thus hypothesized that Wolbachia once associated with T. mercedesae or the ancestor, and the parts of Wolbachia genes were integrated into the mite genome by nuclear Wolbachia transfers (nuwts) during evolution. We would like to propose that Wolbachia has been replaced with Rickettsiella grylli-like bacterium as the symbiont. Since we extensively washed the mites for genome sequencing with acetone prior to genomic DNA extraction (Lines 430-431 in the Methods section of the revised manuscript) and R. grylli-like bacterial DNA was only found in the female but not male mite genome, it is unlikely that the bacterium was

a contaminant. We have explained these in the revised manuscript (Lines 357-374).

370-385: This is well described and fascinating.

387-408: The conclusions are excellently described and well supported.

410-564: Methods: Excellently described. This section will be very useful to anyone wanting to perform similar analyses on their own species of interest. Thank you for providing this level of detail. I don't know anything about proteomics, so I don't feel qualified to comment on 565-608, but it looks sound.

Thanks also for making the files available at ftp://climb.genomics.cn/ . Although I did not have a chance to dig around in there in detail, I did notice that ftp://climb.genomics.cn/De_novo_transcriptome_assemblies/ has very different assembly sizes for Adult_females_#1 (54 Mb) vs Adult_females_#2 (93 Mb). Similarly Adult_males_#1 Adult_males_#2 are 45 Mb and 279 Mb respectively, and Nymphs_#1 and Nymphs_#2 are 57 Mb and 238 Mb - I might be missing something obvious (that these are from different assemblers or diff body parts? But I didn't see that described in the manuscript)

Authors: We used the same method for de novo assembly of all RNA-seq reads with Trinity. The assembly sizes of adult male #2 and nymph #2 samples are quite larger than those of other samples because they were sequenced by Illumina Hiseq 4000 (Lines 458-461 in the revised manuscript), and thus have the read length of 150 bp compared to 90 bp of other samples (Supplementary Table 1). This apparently resulted in increasing the assembly sizes. We assume the female #2 sample has larger assembly size than the female #1 sample because its sequence reads contain less DWV-derived sequences than the female #1 sample.

Finally as the Editor suggested, we have added the images of T. mercedesae as a new Figure 1 in the revised manuscript.
