# Supplementary material for: Draft genome of the honey bee ectoparasitic mite, Tropilaelaps mercedesae, is shaped by the parasitic life history
Source: Gigascience. 2017 Feb 22;6(3):1–17. doi: 10.1093/gigascience/gix008 (PMC5467014; doi:10.1093/gigascience/gix008)
Supplement: Response_to_Reviewer_Comments_Revision_1.pdf [file gix008_Response_to_Reviewer_Comments_Revision_1.pdf]

The Ixodes scapularis genome paper published in Nature Communications has been cited at lines 116, 198, 203, 552, and 976 in the second revised manuscript. In addition, the GenBank project accession number has been added at line 552.

The accession number for our PRIDE dataset remains the same (PXD004997) without the DOI.It has been described in the Methods (line 671) and Data availability (line 676) sections.

DOI information for our dataset in GigaDB has been added as reference 100.
