# Supplementary material for: Draft genome of the honey bee ectoparasitic mite, Tropilaelaps mercedesae, is shaped by the parasitic life history
Source: Gigascience. 2017 Feb 22;6(3):1–17. doi: 10.1093/gigascience/gix008 (PMC5467014; doi:10.1093/gigascience/gix008)
Supplement: Reviewer_1_Report_(Original_Submission).pdf [file gix008_Reviewer_1_Report_(Original_Submission).pdf]

Reviewer Report

Title: "Draft genome of the honey bee ectoparasitic mite, Tropilaelaps mercedesae, is shaped by the parasitic life history"

Version: Original Submission **Date:** 10/18/2016

Reviewer name: Ben Mans

Reviewer Comments to Author:

The authors describe the draft genome of the honey bee mite Tropilaelaps mercedesae. The sequencing statistics are acceptable. They also performed proteomics and RNA-seq to validate transcripts, used flow cytometry to estimate genome size and performed comparative analysis with other arachnid genomes. This study is worthwhile and contribute significantly to existing mite genomics. I therefore recommend acceptance once some issues have been addressed.

1. Page 3, line 88, 92: Will it be possible to include the statistics of other arachnid genomes in Table 1?

2. Page 4, line 100: Can the coverage range or percentage of reads that constitute repetitive elements be indicated in the text?

3. Page 4, line 131: The study by Hoy et al. (2016) indicated two different divergence dates depending on different hypotheses as proposed by Jeyaprakash and Hoy (2009). The first assumes that the Acari are monophyletic and for this a date of 336+-26 MYA was proposed for the Parasitiformes and 395+-24 for their divergence from the Acariformes. The second assumes that the Acari are not monophyletic and that the Parasitiformes then diverged from other arachnids (spiders) 459+-18 MYA. The current study states that parasitiformes diverged from other Arachnida 302 MYA, which is not similar to Hoy et al. (2016). The current study is based on a fossil calibration of 311-501 based on the oldest spider fossil. The node is younger than this estimate which indicates an underestimation in the molecular clock analysis. This date would also only hold if spiders and parasitiformes are truly sister genera. However, this relationship is not robust, since spiders and acariformes are the only other arachnids included in the tree. If for example, scorpions are closer to ticks than spiders, the minimum divergence date would have been ~428 MYA. Because no other arachnids were included, this dating is biased towards a younger divergence date. The analysis has to be expanded to other arachnids or this part of the study needs to be discarded. The most that can be said with some confidence at this point, is that the data support other studies that indicated the paraphyly of the the Acari.

4. Page 4, line 136: Are these 15506 unique gene families or orthologous groups? Note that different orthologs can have related paralogs in the same genome that have the same protein folds and descend from common ancestors, and therefore belong to the same gene/protein family.

5. Page 4, line 140: Are these unclustered genes unique (distinct protein families/singletons), or are they paralogs of orthologs and therefore recent gene duplicates/lineage specific expansions? Their GO annotation suggest that these are recent duplicates. Are there any unique/singleton genes present that cannot be classified?

6. Although lengthy, I enjoyed the comparative analysis part of the study and which address good issues in mite biology that will be useful to all researchers in this field.

7. Figure 1: Arthropod specific…., Species specific…

Methods

Are the methods appropriate to the aims of the study, are they well described, and are necessary controls included? Yes

Conclusions

Are the conclusions adequately supported by the data shown? Yes

Reporting Standards

Does the manuscript adhere to the journal's guidelines on minimum standards of reporting? Yes

Statistics

Are you able to assess all statistics in the manuscript, including the appropriateness of statistical tests used? Yes, and I have assessed the statistics in my report.

Quality of Written English

Please indicate the quality of language in the manuscript: Acceptable

Declaration of Competing Interests

Please complete a declaration of competing interests, considering the following questions:

- Have you in the past five years received reimbursements, fees, funding, or salary from an organisation that may in any way gain or lose financially from the publication of this manuscript, either now or in the future?
- Do you hold any stocks or shares in an organisation that may in any way gain or lose financially from the publication of this manuscript, either now or in the future?
- Do you hold or are you currently applying for any patents relating to the content of the manuscript?
- Have you received reimbursements, fees, funding, or salary from an organization that holds or has applied for patents relating to the content of the manuscript?

- Do you have any other financial competing interests?
- Do you have any non-financial competing interests in relation to this paper?

If you can answer no to all of the above, write 'I declare that I have no competing interests' below. If your reply is yes to any, please give details below.

I declare that I have no competing interests

I agree to the open peer review policy of the journal. I understand that my name will be included on my report to the authors and, if the manuscript is accepted for publication, my named report including any attachments I upload will be posted on the website along with the authors' responses. I agree for my report to be made available under an Open Access Creative Commons CC-BY license (http://creativecommons.org/licenses/by/4.0/). I understand that any comments which I do not wish to be included in my named report can be included as confidential comments to the editors, which will not be published.

I agree to the open peer review policy of the journal

To further support our reviewers, we have joined with Publons, where you can gain additional credit to further highlight your hard work (see: https://publons.com/journal/530/gigascience). On publication of this paper, your review will be automatically added to Publons, you can then choose whether or not to claim your Publons credit. I understand this statement.

Yes
