# Supplementary material for: Draft genome of the honey bee ectoparasitic mite, Tropilaelaps mercedesae, is shaped by the parasitic life history
Source: Gigascience. 2017 Feb 22;6(3):1–17. doi: 10.1093/gigascience/gix008 (PMC5467014; doi:10.1093/gigascience/gix008)
Supplement: Reviewer_2_Report_(Original_Submission).pdf [file gix008_Reviewer_2_Report_(Original_Submission).pdf]

Reviewer Report

Title: "Draft genome of the honey bee ectoparasitic mite, Tropilaelaps mercedesae, is shaped by the parasitic life history"

Version: Original Submission **Date:** 10/20/2016

Reviewer name: Michael Brewer

Reviewer Comments to Author:

Dr. Kadowaki et al.,

The work described in your manuscript comprises an interesting system investigated using appropriate methods. The conclusions are appropriate, if not conservative. I found the manuscript to be well-written, but a few issues need to be addressed.

In general, the methods section requires far more detail. The subsections appear to have been written by several people and differ greatly in the amount of detail given. For example, specifics concerning the genome/transcriptome sequencing are lacking (how many samples were sequenced; how many lanes; how was the RNA extracted and prepped?), VELVET parameters for genome assembly, and MAKER parameters are all missing. Additionally, in some subsections, software versions are not indicated.

Some subsections should be combined or rearranged. "Construction of phylogenetic trees" and "evolutionary analyses" have a lot of overlap and are somewhat confusing as currently written. I would recommend substituting/reorganizing them with/as "Species tree phylogenetics and divergence time estimations", "Gene family expansion and positive selection tests", and "Gene family phylogenetics".

The RNA-seq methods lack details concerning basic experimental design, replication, and quality control.

The proteomics subsection contains a more appropriate amount information than the rest of the methods, which are variable themselves.

Lastly, the reference format needs to be standardized.

Again, this work is well done and important. However, one of the tenants of Gigascience is reproducibility, so this current draft needs a more attention paid to methods in almost all regards.

Methods

Are the methods appropriate to the aims of the study, are they well described, and are necessary controls included? No

Conclusions

Are the conclusions adequately supported by the data shown? Yes

Reporting Standards

Does the manuscript adhere to the journal's guidelines on minimum standards of reporting? No

Statistics

Are you able to assess all statistics in the manuscript, including the appropriateness of statistical tests used? Yes, and I have assessed the statistics in my report.

Quality of Written English

Please indicate the quality of language in the manuscript: Acceptable

Declaration of Competing Interests

Please complete a declaration of competing interests, considering the following questions:

- Have you in the past five years received reimbursements, fees, funding, or salary from an organisation that may in any way gain or lose financially from the publication of this manuscript, either now or in the future?
- Do you hold any stocks or shares in an organisation that may in any way gain or lose financially from the publication of this manuscript, either now or in the future?
- Do you hold or are you currently applying for any patents relating to the content of the manuscript?
- Have you received reimbursements, fees, funding, or salary from an organization that holds or has applied for patents relating to the content of the manuscript?
- Do you have any other financial competing interests?
- Do you have any non-financial competing interests in relation to this paper?

If you can answer no to all of the above, write 'I declare that I have no competing interests' below. If your reply is yes to any, please give details below.

I declare that I have no competing interests

I agree to the open peer review policy of the journal. I understand that my name will be included on my report to the authors and, if the manuscript is accepted for publication, my named report including any attachments I upload will be posted on the website along with the authors' responses. I agree for my report to be made available under an Open Access Creative Commons CC-BY license (http://creativecommons.org/licenses/by/4.0/). I understand that any comments which I do not wish to be included in my named report can be included as confidential comments to the editors, which will not be published.

I agree to the open peer review policy of the journal

To further support our reviewers, we have joined with Publons, where you can gain additional credit to further highlight your hard work (see: https://publons.com/journal/530/gigascience). On publication of this paper, your review will be automatically added to Publons, you can then choose whether or not to claim your Publons credit. I understand this statement.

Yes
