# Supplementary material for: Draft genome of the honey bee ectoparasitic mite, Tropilaelaps mercedesae, is shaped by the parasitic life history
Source: Gigascience. 2017 Feb 22;6(3):1–17. doi: 10.1093/gigascience/gix008 (PMC5467014; doi:10.1093/gigascience/gix008)
Supplement: Reviewer_3_Report_(Original_Submission).pdf [file gix008_Reviewer_3_Report_(Original_Submission).pdf]

Reviewer Report

Title: "Draft genome of the honey bee ectoparasitic mite, Tropilaelaps mercedesae, is shaped by the parasitic life history"

Version: Original Submission **Date:** 10/26/2016

Reviewer name: Sujai Kumar, Ph.D.

Reviewer Comments to Author:

Dear Authors

Congratulations on your research article on the draft genome of a parasite of honey bees. Your study lays out the importance of understanding this organism, both from the specific point of view of helping honey bees, but also the general field of parasite genomics. Overall, this is a high quality genome paper with best practice methods for contamination checks, assembly, annotation, functional, and comparative genomics. Although the paper is well written, I feel it could do with some very light editing in a few sections that I have tried to highlight below.

Some specific comments (I am using the original line numbers which go from 1-941, rather than the 1-65 line numbers on each page that seem to have been added by the submission software). They are quite minor and should be easy to address before this manuscript is accepted and published.

51: Varroa destructor seems to be a non sequitur here when it hasn't been mentioned in the title or up to this point. I would suggest introducing it with a phrase.

57: Background - needs light editing. The emphasis seems to now be on V destructor rather than T mercedesae (which is in the title).

75: change 'was' to 'were'

84: Suggest changing "Each of dual indexed paired-end DNA library was" to "Dual indexed paired-end DNA libraries were"

95: Suggest changing sequencing depth to k-mer depth.

96: Just a comment - I would emphasise that because 94% of the sequencing reads map back to the 353 Mb asssembly, you have greater evidence that the 'collapsing' of the assembly was because of repetitive sequences. I am a bit surprised however at this discrepancy in kmer based sizing and genome assembly sizing. Could it be that you used very different k-mers in the genome size estimation and in the final assembly? Alternatively, the assembler may have had aggressive settings for bubble popping. I tried to look for the velvet settings used in the Methods section but couldn't find them - apologies if I missed them elsewhere. Please include these.

121: Comment and disclaimer: I don't know much about arthropod phylogenetics, but the methods/conclusions described here seem valid.

135: Comment: OrthoMCL seems to be the most commonly used software for this type of analysis, but I hope more studies will use OrthoFinder in the future as it is more sensitive and specific in my (limited) experience.

121-165: Comment: A really well described comparative genomics section.

166-228: Comment and disclaimer: I don't know much about sensory systems but as a non-expert I was able to follow the methods and results, and I agree with the results, tables, figures and conclusions.

194: Suggest rephrasing : "Without orthology" seems odd when the figure shows Dm and Tm proteins in the same tree.

229-344: Same comment and disclaimer as above.

345: Glad to see TAGC plots used here (I think all genome sequencing projects should do them, or something like them, eg CONCOCT, Anvi'o by default). However, if it is not too much trouble (as you probably have the coverage and seq similarity hit files already), could I request you to use the updated blobtools suite at https://github.com/DRL/blobtools ? The plots are easier to interpret and provide

much more information on the span/number of contigs in each blob. It will also help visualise the high repeat content. It is quite quick to run diamond blastx against uniref90 to colour the blobs better so that fewer contigs are left unannotated.

345-369: Can you summarise the findings here better? I think what this section is saying is that there is possibly a cobiont/symbiont/endosymbiont (more simiar to Rickettsiella grylli rather than Wolbachia) - how do you know it is not a contaminant? A blobtools plot with identification at the level of clade would be really helpful in resolving this. In addition, there are Nuwts that are integrated into the genomic DNA so this mite may have had a Wolbachia endosymbiont in the past. Is that the conclusion?

370-385: This is well described and fascinating.

387-408: The conclusions are excellently described and well supported.

410-564: Methods: Excellently described. This section will be very useful to anyone wanting to perform similar analyses on their own species of interest. Thank you for providing this level of detail. I don't know anything about proteomics, so I don't feel qualified to comment on 565-608, but it looks sound.

Thanks also for making the files available at ftp://climb.genomics.cn/ . Although I did not have a chance to dig around in there in detail, I did notice that ftp://climb.genomics.cn/De_novo_transcriptome_assemblies/ has very different assembly sizes for Adult_females_#1 (54 Mb) vs Adult_females_#2 (93 Mb). Similarly Adult_males_#1 Adult_males_#2 are 45 Mb and 279 Mb respectively, and Nymphs_#1 and Nymphs_#2 are 57 Mb and 238 Mb - I might be missing something obvious (that these are from different assemblers or diff body parts? But I didn't see that described in the manuscript)

Best wishes,

Sujai Kumar

University of Edinburgh

Methods

Are the methods appropriate to the aims of the study, are they well described, and are necessary controls included? Yes

Conclusions

Are the conclusions adequately supported by the data shown? Yes

Reporting Standards

Does the manuscript adhere to the journal's guidelines on minimum standards of reporting? Yes

Statistics

Are you able to assess all statistics in the manuscript, including the appropriateness of statistical tests used? There are no statistics in the manuscript.

Quality of Written English

Please indicate the quality of language in the manuscript: Acceptable

Declaration of Competing Interests

Please complete a declaration of competing interests, considering the following questions:

- Have you in the past five years received reimbursements, fees, funding, or salary from an organisation that may in any way gain or lose financially from the publication of this manuscript, either now or in the future?
- Do you hold any stocks or shares in an organisation that may in any way gain or lose financially from the publication of this manuscript, either now or in the future?
- Do you hold or are you currently applying for any patents relating to the content of the manuscript?
- Have you received reimbursements, fees, funding, or salary from an organization that holds or has applied for patents relating to the content of the manuscript?
- Do you have any other financial competing interests?
- Do you have any non-financial competing interests in relation to this paper?

If you can answer no to all of the above, write 'I declare that I have no competing interests' below. If your reply is yes to any, please give details below.

I declare that I have no competing interests

I agree to the open peer review policy of the journal. I understand that my name will be included on my report to the authors and, if the manuscript is accepted for publication, my named report including any attachments I upload will be posted on the website along with the authors' responses. I agree for my report to be made available under an Open Access Creative Commons CC-BY license

(http://creativecommons.org/licenses/by/4.0/). I understand that any comments which I do not wish to be included in my named report can be included as confidential comments to the editors, which will not be published.

I agree to the open peer review policy of the journal

To further support our reviewers, we have joined with Publons, where you can gain additional credit to further highlight your hard work (see: https://publons.com/journal/530/gigascience). On publication of this paper, your review will be automatically added to Publons, you can then choose whether or not to claim your Publons credit. I understand this statement.

Yes
