# Supplementary material for: Draft genome of the honey bee ectoparasitic mite, Tropilaelaps mercedesae, is shaped by the parasitic life history
Source: Gigascience. 2017 Feb 22;6(3):1–17. doi: 10.1093/gigascience/gix008 (PMC5467014; doi:10.1093/gigascience/gix008)
Supplement: Supplemental material — Supplementary data are available at GIGSCI online. [file gix008_Supp.zip › Dong et al_Supplementary Tables_revised.doc]

Supplementary Table 1. Genome and transcriptome sequencing data by Illumina.

|  | **Library** | **DNA/RNA source** | **Depth** | **Sequencing type** | **Insert size (bp)** | **Read length (bp)** | **Sequence reads** |
| --- | --- | --- | --- | --- | --- | --- | --- |
| **DNA-seq** | Male | Single mite | 60X | Paired end | 1,239 | 100 | 274,538,548 |
|  |  |  |  | Single end | - | 100 | 120,682,056 |
|  | Female | Single mite | 62X | Paired end | 1,006 | 100 | 311,718,384 |
|  |  |  |  | Single end | - | 100 | 139,104,931 |
| **RNA-seq** | Adult males #1 | 20 to 30 mites | - | Paired end | Overlap | 90 | 48,725,296 |
|  | Adult males #2 | 20 to 30 mites | - | Paired end | Overlap | 150 | 27,830,722 |
|  | Adult females #1 | 20 to 30 mites | - | Paired end | Overlap | 90 | 48,503,476 |
|  | Adult females #2 | 20 to 30 mites | - | Paired end | Overlap | 90 | 48,100,134 |
|  | Nymphs #1 | 20 to 30 mites | - | Paired end | Overlap | 90 | 54,915,998 |
|  | Nymphs #2 | 20 to 30 mites | - | Paired end | Overlap | 150 | 27,830,722 |

Supplementary Table 2. General statistics of gene set and the integrated prediction (Homolog based prediction is not included).

| **Method** | **Gene set** | **Number** | **Average number of exons per gene** | **Average length (bp)** | | | |
| --- | --- | --- | --- | --- | --- | --- | --- |
|  |  |  |  | **Gene** | **CDS** | **Exon** | **Intron** |
| ***De novo*** | **Augustus** | 32,561 | 3 | 2,743 | 837 | 268 | 918 |
|  | **Genemark** | 79,928 | 4 | 2,218 | 525 | 184 | 470 |
|  | **SNAP** | 67,358 | 2 | 2,964 | 548 | 224 | 1,441 |
| **RNA-seq** | | 37,857 | 5 | 6,603 | 996 | 473 | 934 |
| **Maker** | | 14,423 | 5 | 5,323 | 1,138 | 381 | 792 |
| **Final gene set** | | 15,190 | 5 | 5,298 | 1,117 | 407 | 788 |

Supplementary Table 3. *T. mercedesae* genome assembly and gene set statistics compared with nine other arachnids.

|  | Acari: Parasitiformes | | | | Acari: Acariformes | | | Araneae | | Scorpiones |
| --- | --- | --- | --- | --- | --- | --- | --- | --- | --- | --- |
| Species | *Tropilaelaps mercedesae* | *Metaseiulus occidentalis* | *Varroa destructor* | *Ixodes scapularis* | *Dermatophagoides farinae* | *Sarcoptes scabiei* | *Tetranychus urticae* | *Stegodyphus mimosarum* | *Acanthoscurria geniculata* | *Mesobuthus martensii* |
| Common name | Small honey bee mite | Western orchard predatory mite | Big honey bee mite | Black-legged deer tick | American house dust mite | Itch mite (scabies) | Two-spotted spider mite | African social velvet spider | Brazilian white- knee tarantula | Chinese scorpion |
| Genome  assembly | na | GCA_000255335.1 | GCA_000181155.1 | GCA_000208615.1 | GCA_000767015.1 | GCA_000828355.1 | GCA_000239435.1 | GCA_000611955.2 | GCA_000661875.1 | GCA_000484575.1 |
| Assembly  method | Velvet v.1.2.07 VelvetOptimiser v.2.2.5 | Celera v.6.1 | Celera v.5.2 | Celera v.4.0 | AllPaths v.39075 Velvet v.1.1.03 SOAPdenovo v.1.05 | Minia v.1.6088 SSPACE Standard v.3.0 | Arachne 20071016 | SOAPdenovo v.2 | SOAPdenovo v.2 | Velvet v.1.1.04 |
| Coverage | 60x | 17.7x | 5x | 6x | 436x | 174x | 8xg | 70x | 70x | 200x |
| Assembled genome size (Mbp) | 353.0 | 151.7 | 294.1 | 1,765.40 | 53.5 | 56.3 | 90.8 | 2,738.70 | 7,178.40 | 925.5  1,128.5 |
| No. contigs | 104,715 | 3,993 | 184,190 | 570,637 | 11,600 | 19,811  18,600 | 2,035 | 174,164 | 12,478,692 | 92,408 |
| Contig N50 (bp) | 8,673 | 200,706 | 2,262 | 2,942 | 8,538 | 11,197  11,600 | 212,780 | 40,146  17,272 | 541  277 | 45,228  43,135 |
| No. scaffolds | 34,155 | 2,211 | na | 369,492 | 515 | na | 640 | 68,653 | na | na |
| Scaffold N50 (bp) | 28,807 | 896,831 | na | 76,228 | 186,342 | na | 2,993,488 | 480,636 | 47,837 | 223,560 |
| No. protein coding genes | 15,190 | 18,338  11,430 | 11,432 | 20,486 | 16,376 | 10,473  10,644 | 18,414  18,224 | 27,135 | 27,235 | 73,821 |
| % CEGs^a^ | 98.39／91.9 | 98.0 / 96.8 | 67.7 / 32.3 | 79.8 / 41.9 | 97.6 / 96.4 | 98.0 / 93.5 | 98.0 / 95.2 | 61.7 / 24.2 | 32.7 / 14.9 | 57.3 / 24.2 |
| % BUSCOs^b^ (assembly) | 63 [4.0],  14,22 | 82.3 [6.4],  8.3, 9.9 | 15.2 [0.5],  15.4, 69.5 | 68.9 [2.4],  21.0, 10.1 | 63.8 [3.5],  7.6, 28.6 | 62.0 [3.0],  8.4, 29.6 | 68.8 [5.8],  9.9, 21.3 | 53.7 [4.0],  27.4, 18.9 | 13.7 [1.9],  10.6, 75.7 | 34.4 [4.0],  23.0, 42.7 |
| % BUSCOs ^b^ (gene set) | 68 [6.8],  13,17 | 82.8 [12.0],  10.6, 6.5 | na | 69.4 [6.7],  23.4, 7.2 | na | 54.0 [4.7],  11.4, 34.6 | 69.3 [11.4],  9.6, 21.0 | 64.6 [10.5],  17.3, 18.1 | na | 23.1 [6.7],  16.4, 60.5 |

a% of 248 CEGMA (Core Eukaryotic Genes Mapping Approach) genes Found / Complete in the assembly.

b% of 2,675 arthropod BUSCOs (Benchmarking Universal Single-Copy Orthologs) Complete [Duplicated], Fragmented, Missing, in the assembly or in the gene set.

Data are referred to reference [1] and this study.

Supplementary Table 4. Identification of the repetitive sequences in *T. mercedesae* genome.

| **Repeat type** | **Length (bp)** | **% of genome** |
| --- | --- | --- |
| **DNA transposon** | 1,160,558 | 0.329 |
| **LINE** | 3,659,707 | 1.037 |
| **SINE** | 8,458 | 0.002 |
| **LTR** | 762,578 | 0.216 |
| **Satellite** | 90 | 2.550 |
| **Simple repeat** | 7,483,749 | 2.120 |
| **Unknown** | 12,122,518 | 3.434 |
| **Small RNA** | 57,046 | 0.016 |
| **Low complexity** | 340,275 | 0.096 |
| **Total** | 25,594,979 | 7.250 |

The results integrate both homology-based and *de novo* predictions. The total lengths of the repetitive sequences and their corresponding percentages in the genome for different categories were calculated based on the assembled genome size, i.e. 353 Mb. The number of mapped reads was counted using bedtools (v2.26.0) with ‘-split’ function depending on bam files generated by Bowtie2.

Supplementary Table 5. Identification of repetitive sequences in the 3975 contigs with normalized converge larger than 20 in the Supplementary Fig. 20 A.

| **Repeat type** | **Length (bp)** | **% of high coverage contigs** |
| --- | --- | --- |
| **DNA transposon** | 7,206 | 0.43 |
| **LINE** | 201,485 | 12.09 |
| **SINE** | 14,165 | 0.85 |
| **LTR** | 2,524 | 0.15 |
| **Satellite** | 0 | 0 |
| **Simple repeat** | 395,443 | 23.74 |
| **Unknown** | 870,215 | 52.21 |
| **Small RNA** | 4,870 | 0.29 |
| **Low complexity** | 10,284 | 0.62 |
| **Total** | 15,06,192 | 90.37 |

The results integrate both homology-based and *de novo* predictions. The total lengths of the repetitive sequences and their corresponding percentages in the genome for different categories were calculated based on the high coverage contig size, i.e. 1.8 Mb.

Supplementary Table 6. Characterization of genes for non-coding RNA; micro RNA (miRNA), transfer RNA (tRNA), ribosomal RNA (rRNA), and small nuclear RNA (snRNA) in *T. mercedesae* genome.

| **Type** | | **Copy #** | **Average length (bp)** | **Total length (bp)** | **% of genome** |
| --- | --- | --- | --- | --- | --- |
| **miRNA** | | 19 | 79.1 | 1503 | 0.000425775 |
| **tRNA** | | 310 | 73.7 | 22850 | 0.006473027 |
| **rRNA** | **18S** | 10 | 204.1 | 2041 | 0.000578181 |
|  | **28S** | 23 | 280.57 | 5453 | 0.001544745 |
|  | **5.8S** | 1 | 154 | 154 | 4.36256E-05 |
|  | **5S** | 95 | 116 | 11024 | 0.003122917 |
|  | **Total** | 129 | 754.67 | 18672 | 0.005289468 |
| **snRNA** | **CD-box** | 8 | 165.5 | 1324 | 0.000375067 |
|  | **HACA-box** | 1 | 135 | 135 | 3.82433E-05 |
|  | **Splicing** | 114 | 128.73 | 15816 | 0.004480411 |
|  | **Total** | 123 | 429.23 | 17275 | 0.004893721 |

Ribosomal RNAs are divided into four sub-classes based on their molecular weight (28S, 18S, 5.8S, and 5S), whereas the snRNAs contain the sub-classes of CD-box, HACA-box, and spliceosomal RNAs. The total length of the non-coding RNA genes and their corresponding percentages in the genome for different categories are calculated based on the assembled genome size, 353 Mb.

Supplementary Table 7. GO enrichment of orthology clusters specifically shared between *T. mercedesae* and *M. occidentalis.*

| **GO-ID** | **Term** | **Category** | ***P*-Value** |
| --- | --- | --- | --- |
| GO:0042302 | structural constituent of cuticle | F | 1.97E-05 |
| GO:0014059 | regulation of dopamine secretion | P | 7.25E-05 |
| GO:0014046 | dopamine secretion | P | 7.25E-05 |
| GO:0050433 | regulation of catecholamine secretion | P | 2.07E-04 |
| GO:0044030 | regulation of DNA methylation | P | 2.45E-04 |
| GO:0048520 | positive regulation of behavior | P | 2.74E-04 |
| GO:0022834 | ligand-gated channel activity | F | 4.55E-04 |
| GO:0015276 | ligand-gated ion channel activity | F | 4.55E-04 |
| GO:0060004 | reflex | P | 4.58E-04 |
| GO:0051952 | regulation of amine transport | P | 4.58E-04 |
| GO:0051780 | behavioral response to nutrient | P | 4.58E-04 |
| GO:0042753 | positive regulation of circadian rhythm | P | 4.58E-04 |
| GO:0045938 | positive regulation of circadian sleep/wake cycle, sleep | P | 4.58E-04 |
| GO:0007637 | proboscis extension reflex | P | 4.58E-04 |
| GO:0005230 | extracellular ligand-gated ion channel activity | F | 6.27E-04 |
| GO:0042197 | halogenated hydrocarbon metabolic process | P | 6.51E-04 |
| GO:0042196 | chlorinated hydrocarbon metabolic process | P | 6.51E-04 |
| GO:0050432 | catecholamine secretion | P | 8.72E-04 |
| GO:0008812 | choline dehydrogenase activity | F | 8.72E-04 |
| GO:0015872 | dopamine transport | P | 8.72E-04 |
| GO:0010884 | positive regulation of lipid storage | P | 9.33E-04 |
| GO:0050802 | circadian sleep/wake cycle, sleep | P | 1.18E-03 |
| GO:0022410 | circadian sleep/wake cycle process | P | 1.18E-03 |
| GO:0019497 | hexachlorocyclohexane metabolic process | P | 1.42E-03 |
| GO:0003993 | acid phosphatase activity | F | 1.42E-03 |
| GO:0042745 | circadian sleep/wake cycle | P | 1.68E-03 |
| GO:0006771 | riboflavin metabolic process | P | 1.85E-03 |
| GO:0042726 | flavin-containing compound metabolic process | P | 1.85E-03 |
| GO:0019433 | triglyceride catabolic process | P | 2.22E-03 |
| GO:0022836 | gated channel activity | F | 2.38E-03 |
| GO:0022803 | passive transmembrane transporter activity | F | 3.25E-03 |
| GO:0015267 | channel activity | F | 3.25E-03 |
| GO:0051937 | catecholamine transport | P | 3.53E-03 |
| GO:1902110 | positive regulation of mitochondrial membrane permeability involved in apoptotic process | P | 3.53E-03 |
| GO:1902108 | regulation of mitochondrial membrane permeability involved in apoptotic process | P | 3.53E-03 |
| GO:0035794 | positive regulation of mitochondrial membrane permeability | P | 3.53E-03 |
| GO:2000706 | negative regulation of dense core granule biogenesis | P | 3.92E-03 |
| GO:0071514 | genetic imprinting | P | 3.92E-03 |
| GO:0006349 | regulation of gene expression by genetic imprinting | P | 3.92E-03 |
| GO:0048842 | positive regulation of axon extension involved in axon guidance | P | 3.92E-03 |
| GO:2000065 | negative regulation of cortisol biosynthetic process | P | 3.92E-03 |
| GO:0032348 | negative regulation of aldosterone biosynthetic process | P | 3.92E-03 |
| GO:0032345 | negative regulation of aldosterone metabolic process | P | 3.92E-03 |
| GO:0044027 | hypermethylation of CpG island | P | 3.92E-03 |
| GO:0044026 | DNA hypermethylation | P | 3.92E-03 |
| GO:1901030 | positive regulation of mitochondrial outer membrane permeabilization | P | 3.92E-03 |
| GO:0036016 | cellular response to interleukin-3 | P | 3.92E-03 |
| GO:0036015 | response to interleukin-3 | P | 3.92E-03 |
| GO:0010614 | negative regulation of cardiac muscle hypertrophy | P | 3.92E-03 |
| GO:0061320 | pericardial nephrocyte differentiation | P | 3.92E-03 |
| GO:0014741 | negative regulation of muscle hypertrophy | P | 3.92E-03 |
| GO:0005216 | ion channel activity | F | 4.10E-03 |
| GO:0022838 | substrate-specific channel activity | F | 4.10E-03 |
| GO:0046503 | glycerolipid catabolic process | P | 4.24E-03 |
| GO:0046464 | acylglycerol catabolic process | P | 4.24E-03 |
| GO:0046461 | neutral lipid catabolic process | P | 4.24E-03 |
| GO:0022839 | ion gated channel activity | F | 4.51E-03 |
| GO:0045475 | locomotor rhythm | P | 5.04E-03 |
| GO:0045187 | regulation of circadian sleep/wake cycle, sleep | P | 5.04E-03 |
| GO:0042749 | regulation of circadian sleep/wake cycle | P | 5.04E-03 |
| GO:0046902 | regulation of mitochondrial membrane permeability | P | 5.04E-03 |
| GO:0006805 | xenobiotic metabolic process | P | 5.28E-03 |
| GO:0010002 | cardioblast differentiation | P | 6.92E-03 |
| GO:0016021 | integral to membrane | C | 6.98E-03 |
| GO:0003073 | regulation of systemic arterial blood pressure | P | 7.08E-03 |
| GO:0006306 | DNA methylation | P | 7.08E-03 |
| GO:0006305 | DNA alkylation | P | 7.08E-03 |
| GO:0005865 | striated muscle thin filament | C | 7.08E-03 |
| GO:0005861 | troponin complex | C | 7.08E-03 |
| GO:0036379 | myofilament | C | 7.08E-03 |
| GO:0010883 | regulation of lipid storage | P | 7.08E-03 |
| GO:0016012 | sarcoglycan complex | C | 7.08E-03 |
| GO:0071466 | cellular response to xenobiotic stimulus | P | 7.25E-03 |
| GO:0031224 | intrinsic to membrane | C | 7.33E-03 |
| GO:0005921 | gap junction | C | 9.21E-03 |
| GO:0006032 | chitin catabolic process | P | 9.21E-03 |
| GO:0004568 | chitinase activity | F | 9.21E-03 |
| GO:0010721 | negative regulation of cell development | P | 9.62E-03 |
| GO:0009410 | response to xenobiotic stimulus | P | 9.72E-03 |

The corrected *P*-value (< 0.01) was calculated by Fisher’s extract test embedded in the Blast2go software desktop version.

Supplementary Table 8. GO enrichment of *T. mercedesae* specific genes identified by orthoMCL.

| **GO Term** | **Description** | **Type** | ***P*-value** |
| --- | --- | --- | --- |
| GO:0042302 | Structural constituent of cuticle | F | 1.30E-04 |
| GO:0000786 | Nucleosome | C | 1.50E-04 |
| GO:1990104 | DNA bending complex | C | 1.50E-04 |
| GO:0031497 | Chromatin assembly | P | 7.20E-04 |
| GO:0020037 | Heme binding | F | 7.20E-04 |
| GO:0044815 | DNA packaging complex | C | 7.20E-04 |
| GO:0046906 | Tetrapyrrole binding | F | 8.30E-04 |
| GO:0006334 | Nucleosome assembly | P | 9.60E-04 |
| GO:0005215 | Transporter activity | F | 9.60E-04 |
| GO:0006820 | Anion transport | P | 1.30E-03 |
| GO:0071944 | Cell periphery | C | 1.50E-03 |
| GO:0006333 | Chromatin assembly or disassembly | P | 1.50E-03 |
| GO:0005886 | Plasma membrane | C | 2.00E-03 |
| GO:0032993 | Protein-DNA complex | C | 2.20E-03 |
| GO:0006811 | Ion transport | P | 2.80E-03 |
| GO:0005230 | Extracellular ligand-gated ion channel activity | F | 2.80E-03 |
| GO:0034728 | Nucleosome organization | P | 3.00E-03 |
| GO:0015698 | Inorganic anion transport | P | 4.20E-03 |
| GO:0065004 | Protein-DNA complex assembly | P | 4.30E-03 |
| GO:0007215 | Glutamate receptor signaling pathway | P | 4.40E-03 |
| GO:0045202 | Synapse | C | 6.10E-03 |
| GO:0055085 | Transmembrane transport | P | 7.20E-03 |
| GO:0022857 | Transmembrane transporter activity | F | 7.20E-03 |
| GO:0006323 | DNA packaging | P | 7.30E-03 |
| GO:0006804 | Peroxidase reaction | P | 7.60E-03 |
| GO:0016684 | Oxidoreductase activity, acting on peroxide as acceptor | F | 7.60E-03 |
| GO:0004601 | Peroxidase activity | F | 7.60E-03 |
| GO:0016209 | Antioxidant activity | F | 9.40E-03 |
| GO:0022834 | Ligand-gated channel activity | F | 1.30E-02 |
| GO:0015276 | Ligand-gated ion channel activity | F | 1.30E-02 |
| GO:0071824 | Protein-DNA complex subunit organization | P | 1.60E-02 |
| GO:0005576 | Extracellular region | C | 1.60E-02 |
| GO:0030054 | Cell junction | C | 3.20E-02 |
| GO:0071103 | DNA conformation change | P | 3.40E-02 |
| GO:0034220 | Ion transmembrane transport | P | 4.70E-02 |

The corrected FDR *P*-value (< 0.05) was calculated by Fisher’s extract test embedded in the Blast2go software desktop version.

Supplementary Table 9. GO enrichment of orthology clusters specifically shared between *T. mercedesae* and *I. scapularis.*

| **GO Term** | **Description** | **Type** | ***P*-Value** |
| --- | --- | --- | --- |
| [GO:0061326](../../../../C:%5CUsers%5CFisherInfo%5CGO%5C0061326) | Renal tubule development | P | 4.00E-06 |
| [GO:0021587](../../../../C:%5CUsers%5CFisherInfo%5CGO%5C0021587) | Cerebellum morphogenesis | P | 8.60E-05 |
| [GO:0035295](../../../../C:%5CUsers%5CFisherInfo%5CGO%5C0035295) | Tube development | P | 1.30E-04 |
| [GO:0060562](../../../../C:%5CUsers%5CFisherInfo%5CGO%5C0060562) | Epithelial tube morphogenesis | P | 1.40E-04 |
| [GO:0072080](../../../../C:%5CUsers%5CFisherInfo%5CGO%5C0072080) | Nephron tubule development | P | 2.00E-04 |
| [GO:0048793](../../../../C:%5CUsers%5CFisherInfo%5CGO%5C0048793) | Pronephros development | P | 2.00E-04 |
| [GO:0035239](../../../../C:%5CUsers%5CFisherInfo%5CGO%5C0035239) | Tube morphogenesis | P | 2.00E-04 |
| [GO:0039020](../../../../C:%5CUsers%5CFisherInfo%5CGO%5C0039020) | Pronephric nephron tubule development | P | 2.50E-04 |
| [GO:0039019](../../../../C:%5CUsers%5CFisherInfo%5CGO%5C0039019) | Pronephric nephron development | P | 2.50E-04 |
| [GO:0021549](../../../../C:%5CUsers%5CFisherInfo%5CGO%5C0021549) | Cerebellum development | P | 2.50E-04 |
| [GO:0072009](../../../../C:%5CUsers%5CFisherInfo%5CGO%5C0072009) | Nephron epithelium development | P | 2.50E-04 |
| [GO:0022037](../../../../C:%5CUsers%5CFisherInfo%5CGO%5C0022037) | Metencephalon development | P | 3.10E-04 |
| [GO:0072073](../../../../C:%5CUsers%5CFisherInfo%5CGO%5C0072073) | Kidney epithelium development | P | 3.10E-04 |
| [GO:0072001](../../../../C:%5CUsers%5CFisherInfo%5CGO%5C0072001) | Renal system development | P | 3.30E-04 |
| [GO:0001655](../../../../C:%5CUsers%5CFisherInfo%5CGO%5C0001655) | Urogenital system development | P | 3.80E-04 |
| [GO:2000768](../../../../C:%5CUsers%5CFisherInfo%5CGO%5C2000768) | Positive regulation of nephron tubule epithelial cell differentiation | P | 4.90E-04 |
| [GO:2000744](../../../../C:%5CUsers%5CFisherInfo%5CGO%5C2000744) | Positive regulation of anterior head development | P | 4.90E-04 |
| [GO:2000742](../../../../C:%5CUsers%5CFisherInfo%5CGO%5C2000742) | Regulation of anterior head development | P | 4.90E-04 |
| [GO:2000698](../../../../C:%5CUsers%5CFisherInfo%5CGO%5C2000698) | Positive regulation of epithelial cell differentiation involved in kidney development | P | 4.90E-04 |
| [GO:0072050](../../../../C:%5CUsers%5CFisherInfo%5CGO%5C0072050) | S-shaped body morphogenesis | P | 4.90E-04 |
| [GO:0072049](../../../../C:%5CUsers%5CFisherInfo%5CGO%5C0072049) | Comma-shaped body morphogenesis | P | 4.90E-04 |
| [GO:0060059](../../../../C:%5CUsers%5CFisherInfo%5CGO%5C0060059) | Embryonic retina morphogenesis in camera-type eye | P | 4.90E-04 |
| [GO:0090009](../../../../C:%5CUsers%5CFisherInfo%5CGO%5C0090009) | Primitive streak formation | P | 4.90E-04 |
| [GO:0097477](../../../../C:%5CUsers%5CFisherInfo%5CGO%5C0097477) | Lateral motor column neuron migration | P | 4.90E-04 |
| [GO:0097476](../../../../C:%5CUsers%5CFisherInfo%5CGO%5C0097476) | Spinal cord motor neuron migration | P | 4.90E-04 |
| [GO:0097475](../../../../C:%5CUsers%5CFisherInfo%5CGO%5C0097475) | Motor neuron migration | P | 4.90E-04 |
| [GO:0021937](../../../../C:%5CUsers%5CFisherInfo%5CGO%5C0021937) | Cerebellar Purkinje cell-granule cell precursor cell signaling involved in regulation of granule cell precursor cell proliferation | P | 4.90E-04 |
| [GO:0021575](../../../../C:%5CUsers%5CFisherInfo%5CGO%5C0021575) | Hindbrain morphogenesis | P | 5.50E-04 |
| [GO:0072006](../../../../C:%5CUsers%5CFisherInfo%5CGO%5C0072006) | Nephron development | P | 5.50E-04 |
| [GO:2000177](../../../../C:%5CUsers%5CFisherInfo%5CGO%5C2000177) | Regulation of neural precursor cell proliferation | P | 5.50E-04 |
| [GO:0060429](../../../../C:%5CUsers%5CFisherInfo%5CGO%5C0060429) | Epithelium development | P | 5.70E-04 |
| [GO:0008283](../../../../C:%5CUsers%5CFisherInfo%5CGO%5C0008283) | Cell proliferation | P | 7.30E-04 |
| [GO:0001705](../../../../C:%5CUsers%5CFisherInfo%5CGO%5C0001705) | Ectoderm formation | P | 8.20E-04 |
| [GO:0021940](../../../../C:%5CUsers%5CFisherInfo%5CGO%5C0021940) | Positive regulation of cerebellar granule cell precursor proliferation | P | 8.20E-04 |
| [GO:0021936](../../../../C:%5CUsers%5CFisherInfo%5CGO%5C0021936) | Regulation of cerebellar granule cell precursor proliferation | P | 8.20E-04 |
| [GO:0002009](../../../../C:%5CUsers%5CFisherInfo%5CGO%5C0002009) | Morphogenesis of an epithelium | P | 8.90E-04 |

The *P*-value (< 0.001) was calculated by Fisher’s extract test embedded in the Blast2go software desktop version.

Supplementary Table 10. Changes of gene family size in *T. mercedesae* (Tm) in comparison with six reference arthropod species, *M. occidentalis* (Mo), *I. scapularis* (Is), *S. mimosarum* (Sm), *T. urticae* (Tu), *A. mellifera* (Am), and *D. melanogaster* (Dm).

| **Gene family** | **Mo** | **Tm** | **Is** | **Sm** | **Tu** | **Am** | **Dm** | ***P*-value** | **Description** |
| --- | --- | --- | --- | --- | --- | --- | --- | --- | --- |
| ORTHOMCL158 | 0 | 21 | 0 | 0 | 0 | 0 | 0 | 0 | Metal ion and DNA binding |
| ORTHOMCL261 | 0 | 16 | 0 | 0 | 0 | 0 | 0 | 0 | Peptide cross-linking in cytoplasm |
| ORTHOMCL391 | 0 | 12 | 0 | 0 | 1 | 0 | 0 | 0 | Metal ion, zinc ion and nucleic acid binding |
| ORTHOMCL467 | 0 | 12 | 0 | 0 | 0 | 0 | 0 | 0 | Peptide cross-linking in cytoplasm; DNA binding in nucleus |
| ORTHOMCL571 | 0 | 10 | 1 | 0 | 0 | 0 | 0 | 0 | Polyphosphate kinase: kinase activity for phosphorylation |
| ORTHOMCL711 | 0 | 10 | 0 | 0 | 0 | 0 | 0 | 0 | Zinc finger protein: nucleic acid binding |
| ORTHOMCL177 | 2 | 10 | 6 | 2 | 0 | 0 | 0 | 8E-04 | Reverse transcriptase：RNA-dependent DNA replication |
| ORTHOMCL980 | 1 | 8 | 0 | 0 | 0 | 0 | 0 | 3E-06 | Zinc ion binding |
| ORTHOMCL2495 | 0 | 7 | 0 | 0 | 0 | 0 | 0 | 2.9E-05 | Peptide cross-linking in cytoplasm |
| ORTHOMCL4818 | 0 | 6 | 0 | 0 | 0 | 0 | 0 | 6E-06 | Unknown |
| ORTHOMCL8 | 120 | 0 | 1 | 1 | 0 | 0 | 0 | 0 | Integrase and reverse transcriptase domains |
| ORTHOMCL24 | 39 | 1 | 1 | 27 | 0 | 0 | 0 | 5E-06 | Integrase and reverse transcriptase domains |
| ORTHOMCL67 | 8 | 0 | 5 | 6 | 2 | 3 | 11 | 1E-04 | Heat shock 70 kda protein cognate 4: response to heat stress |
| ORTHOMCL58 | 8 | 0 | 4 | 26 | 0 | 0 | 0 | 1E-04 | DNA binding; DDE superfamily endonuclease |
| ORTHOMCL32 | 54 | 0 | 0 | 0 | 0 | 0 | 0 | 1E-04 | Nucleic acid binding; protein dimerization activity; Ribonuclease H-like domain |
| ORTHOMCL18 | 70 | 2 | 2 | 0 | 0 | 0 | 0 | 1E-04 | Integrase and reverse transcriptase domains |

Gene families in white and grey background represent the ones expanded and contracted in *T. mercedesae*, respectively. Estimating changes in gene family size was performed by CAFE calculation with overall *P*-value < 0.01 and branch specific *P*-values < 0.001. Gene description was obtained by integration of top Blast hit and the GO and Pfam annotations.

Supplementary Table 11. Detailed information of four positively selected genes in *T. mercedesae*.

| **Orthomcl cluster** | **Gene ID** | **d*N*/d*S* Ratio** | **Putative function** |
| --- | --- | --- | --- |
| ORTHOMCL2609 | Tm_01377 | 1.0194 | Thioredoxin-related transmembrane protein 1-like; It has one thioredoxin-like domain with oxidoreductase activity, playing an important role in proper disulfide bond formation. |
| ORTHOMCL2604 | Tm_10257 | 1.0212 | Tyrosine-protein kinase src64b-like; it may play a role in the development of *Drosophila* mushroom body and oogenesis. |
| ORTHOMCL4405 | Tm_07523 | 3.9205 | Cytosolic endo-beta-n-acetylglucosaminidase-like, involved in the processing of free oligosaccharides in the cytosol. |
| ORTHOMCL2644 | Tm_07693 | 9.7219 | Isocitrate dehydrogenase that catalyzes the oxidative decarboxylation of isocitrate, producing alpha-ketoglutarate (α-ketoglutarate) and CO_2_. |

Supplementary Table 12. Characterization of *T. mercedesae* cytochrome P450 genes.

| **Scaffold ID** | **Tandem cluster** | **Gene ID** | **CYP clan** | **Expression** | **Size (aa)** | **Note** |
| --- | --- | --- | --- | --- | --- | --- |
| Scaffold0000000258 | - | Tm_00041 | CYP2 | Yes | 612 | Complete |
| Scaffold0000001158 | - | Tm_15196 | CYP2 | No | 62 | Fragment |
| Scaffold0000003393 | - | Tm_15193 | CYP2 | No | 73 | Internal stop codon |
| Scaffold0000005240 | - | Tm_00664 | CYP2 | Yes | 464 | Complete |
| Scaffold0000005442 | - | Tm_08470 | CYP2 | Yes | 607 | Complete |
| Scaffold0000006696 | - | Tm_15194 | CYP2 | No | 117 | Internal stop codon |
| Scaffold0000015432 | - | Tm_11277 | CYP2 | Yes | 489 | Complete |
| Scaffold0000008201 | Cluster 2 | Tm_03434 | CYP3 | Yes | 547 | Complete |
| Scaffold0000008201 | Cluster 2 | Tm_09150 | CYP3 | Yes | 550 | Complete |
| Scaffold0000008201 | Cluster 2 | Tm_15190 | CYP3 | Yes | 324 | Complete |
| Scaffold0000010685 | Cluster 4 | Tm_15004 | CYP3 | No | 118 | Incompleted N-terminal and C-terminal; fragment |
| Scaffold0000010685 | Cluster 4 | Tm_10021 | CYP3 | Yes | 532 | Complete |
| Scaffold0000010685 | Cluster 4 | Tm_10022 | CYP3 | Yes | 284 | Incompleted N-terminal |
| Scaffold0000010339 | Cluster 5 | Tm_09891 | CYP3 | Yes | 513 | Complete |
| Scaffold0000010339 | Cluster 5 | Tm_03629 | CYP3 | No | 157 | Internal deletion |
| Scaffold0000013932 | Cluster 6 | Tm_03924 | CYP3 | No | 106 | Incompleted N-terminal; fragment |
| Scaffold0000013932 | Cluster 6 | Tm_15195 | CYP3 | No | 145 | Internal stop codon |
| Scaffold0000002746 | - | Tm_07004 | CYP3 | Yes | 494 | Complete |
| Scaffold0000004790 | - | Tm_07853 | CYP3 | Yes | 239 | Incompleted C-terminal |
| Scaffold0000011341 | - | Tm_01372 | CYP3 | Yes | 501 | Complete |
| Scaffold0000015709 | - | Tm_12854 | CYP3 | Yes | 273 | Incompleted N-terminal |
| Scaffold0000018584 | - | Tm_14705 | CYP3 | Yes | 139 | Incompleted N-terminal and C-terminal; fragment |
| Scaffold0000020747 | - | Tm_14091 | CYP3 | Yes | 542 | Complete |
| Scaffold0000025786 | - | Tm_04218 | CYP3 | No | 206 | Incompleted N-terminal |
| Scaffold0000027228 | - | Tm_08589 | CYP3 | No | 101 | Incompleted N-terminal; fragment |
| Scaffold0000027778 | - | Tm_15044 | CYP3 | No | 103 | Incompleted N-terminal and C-terminal; fragment |
| Scaffold0000000842 | Cluster 1 | Tm_02528 | CYP4 | Yes | 356 | Incompleted N-terminal |
| Scaffold0000000842 | Cluster 1 | Tm_02530 | CYP4 | Yes | 405 | Incompleted C-terminal |
| Scaffold0000010068 | Cluster 3 | Tm_01157 | CYP4 | Yes | 276 | Incompleted N-terminal |
| Scaffold0000010068 | Cluster 3 | Tm_09780 | CYP4 | No | 64 | Incompleted C-terminal; fragment |
| Scaffold0000000108 | - | Tm_05131 | CYP4 | Yes | 523 | Complete |
| Scaffold0000001225 | - | Tm_05802 | CYP4 | Yes | 157 | Incompleted C-terminal; fragment |
| Scaffold0000002195 | - | Tm_00300 | CYP4 | Yes | 522 | Complete |
| Scaffold0000002651 | - | Tm_06642 | CYP4 | Yes | 145 | Incompleted N-terminal; fragment |
| Scaffold0000006476 | - | Tm_03283 | CYP4 | Yes | 217 | Incompleted N-terminal and C-terminal |
| Scaffold0000006609 | - | Tm_08626 | CYP4 | No | 58 | Incompleted C-terminal; fragment |
| Scaffold0000006794 | - | Tm_01048 | CYP4 | Yes | 550 | Complete |
| Scaffold0000009676 | - | Tm_15198 | CYP4 | No | 65 | Incompleted N-terminal and C-terminal; fragment |
| Scaffold0000012018 | - | Tm_01415 | CYP4 | Yes | 381 | Complete |
| Scaffold0000012454 | - | Tm_15199 | CYP4 | No | 69 | Incompleted N-terminal and C-terminal; fragment |
| Scaffold0000013246 | - | Tm_01436 | CYP4 | Yes | 524 | Complete |
| Scaffold0000013315 | - | Tm_11223 | CYP4 | Yes | 540 | Complete |
| Scaffold0000013604 | - | Tm_01583 | CYP4 | Yes | 490 | Complete |
| Scaffold0000024534 | - | Tm_15200 | CYP4 | No | 58 | Incompleted N-terminal; fragment |
| Scaffold0000026310 | - | Tm_06697 | CYP4 | Yes | 491 | Complete |
| Scaffold0000032351 | - | Tm_15191 | CYP4 | Yes | 535 | Complete |
| Scaffold0000000576 | - | Tm_15192 | Mito. | No | 68 | Internal stop codon |
| Scaffold0000002181 | - | Tm_15197 | Mito. | No | 60 | Incompleted N-terminal and C-terminal; fragment |
| Scaffold0000004081 | - | Tm_07765 | Mito. | Yes | 312 | Incompleted C-terminal |
| Scaffold0000004970 | - | Tm_07959 | Mito. | No | 224 | Incompleted N-terminal |
| Scaffold0000006918 | - | Tm_01118 | Mito. | No | 462 | Complete |
| Scaffold0000008953 | - | Tm_10252 | Mito. | Yes | 266 | Incompleted C-terminal |
| Scaffold0000015613 | - | Tm_11295 | Mito. | Yes | 289 | Incompleted N-terminal |
| Scaffold0000015651 | - | Tm_11316 | Mito. | Yes | 232 | Incompleted N-terminal |
| Scaffold0000015784 | - | Tm_13084 | Mito. | Yes | 515 | Complete |
| Scaffold0000018584 | - | Tm_13131 | Mito. | No | 194 | Incompleted N-terminal; fragment |

If a cytochrome P450 gene contains more than one read per million in the replicates, the gene is considered to be expressed and shown with ‘Yes’. Tandemly duplicated cytochrome P450 genes are not separated by other genes. In the column of Note, ‘fragment’ indicates a cytochrome P450 with less than 200 amino acids, and ‘complete’ means a cytochrome P450 with the intact P450 domain. The unexpressed fragmented cytochrome P450 genes, and those with internal deletions or stop codons are considered as pseudogenes which are highlighted with gray background.

Supplementary Table 13. Changes of gene family size in *T. mercedesae* (Tm) in comparison with two other Parasitiformes, *M. occidentalis* (Mo) and *I. scapularis* (Is).

| **Gene family** | **Mo** | **Tm** | **Is** | ***P*-value** | **Description** |
| --- | --- | --- | --- | --- | --- |
| ORTHOMCL158 | 0 | 21 | 0 | 0 | Metal ion and DNA binding |
| ORTHOMCL261 | 0 | 16 | 0 | 0 | Peptide cross-linking in cytoplasm |
| ORTHOMCL391 | 0 | 12 | 0 | 0 | Metal ion, zinc ion and nucleic acid binding |
| ORTHOMCL467 | 0 | 12 | 0 | 0 | Peptide cross-linking in cytoplasm; DNA binding in nucleus |
| ORTHOMCL571 | 0 | 10 | 1 | 1E-05 | Polyphosphate kinase: kinase activity for phosphorylation |
| ORTHOMCL711 | 0 | 10 | 0 | 1E-05 | Zinc finger protein: nucleic acid binding |
| ORTHOMCL980 | 1 | 8 | 0 | 7E-04 | Zinc ion binding |
| ORTHOMCL2495 | 0 | 7 | 0 | 1E-05 | Multiple banded antigen |
| ORTHOMCL4818 | 0 | 6 | 0 | 1E-04 | Unknown |
| ORTHOMCL7149 | 0 | 4 | 0 | 3E-4 | Unknown |
| ORTHOMCL7160 | 0 | 4 | 0 | 3E-4 | Ribonuclease |
| ORTHOMCL7161 | 0 | 4 | 0 | 3E-4 | Zinc finger protein: nucleic acid binding |
| ORTHOMCL7163 | 0 | 4 | 0 | 3E-4 | Serine-type endopeptidase activity for proteolysis |
| ORTHOMCL8 | 120 | 0 | 1 | 0 | Integrase and reverse transcriptase domains |
| ORTHOMCL24 | 39 | 1 | 1 | 3E-05 | Integrase and reverse transcriptase domains |
| ORTHOMCL67 | 8 | 0 | 5 | 5E-05 | Heat shock 70 kda protein cognate 4: response to heat stress |
| ORTHOMCL58 | 8 | 0 | 4 | 9E-04 | DNA binding; DDE superfamily endonuclease |
| ORTHOMCL32 | 54 | 0 | 0 | 0 | Nucleic acid binding; protein dimerization activity; Ribonuclease H-like domain |
| ORTHOMCL18 | 70 | 2 | 2 | 0 | Integrase and reverse transcriptase domains |
| ORTHOMCL40 | 44 | 0 | 0 | 3E-06 | Telomere maintenance; DNA helicase activity; DNA repair |
| ORTHOMCL209 | 9 | 0 | 9 | 3E-06 | DNA binding; DDE superfamily endonuclease |
| ORTHOMCL210 | 11 | 0 | 7 | 3E-06 | Cytochrome P450 |
| ORTHOMCL175 | 16 | 0 | 3 | 5E-05 | ATP binding; telomere maintenance; DNA helicase activity; DNA repair |
| ORTHOMCL114 | 23 | 0 | 3 | 5E-05 | Protein dimerization activity; nucleic acid binding |
| ORTHOMCL108 | 3 | 0 | 9 | 9E-04 | Nuclease activity |
| ORTHOMCL476 | 5 | 0 | 6 | 9E-04 | Retinol dehydrogenase 12-like; dehydrogenase |
| ORTHOMCL135 | 21 | 0 | 1 | 9E-04 | Zinc finger domain |
| ORTHOMCL109 | 27 | 0 | 0 | 9E-04 | Single-stranded DNA-binding heterotrimeric complex |

The expanded and contracted gene families in *T. mercedesae* are shown by white and grey backgrounds, respectively. Estimating the changes in gene family size was performed by CAFE calculation with overall *P*-value < 0.01 and branch specific *P*-values < 0.001. Gene description was obtained by integration of top Blast hit and the GO and Pfam annotations.

Supplementary Table 14. Pair-wise comparisons of mRNA levels of *T. mercedesae* P450 between adult females, adult males, and nymphs.

| **Gene ID** | **CYP clans** | **Pairwise comparisons of mRNA level** | | |
| --- | --- | --- | --- | --- |
|  |  | **Females vs. Males** | **Females vs. Nymphs** | **Males vs. Nymphs** |
| Tm_00041 | CYP2 clan | 5.50E-04 (Males) | 8.99E-10 (Nymphs) | 2.20E-04 (Nymphs) |
| Tm_00664 | CYP2 clan | 9.18E-01 | 3.17E-02 (Females) | 3.95E-02 (Males) |
| Tm_08470 | CYP2 clan | 2.34E-01 | 3.95E-01 | 6.67E-02 |
| Tm_11277 | CYP2 clan | 4.54E-01 | 5.00E-01 | 9.81E-01 |
| Tm_10021 | CYP3 clan | - | 5.22E-05 (Females) | 2.22E-08 (Males) |
| Tm_10022 | CYP3 clan | 2.95E-01 | 1.22E-03 (Females) | 2.34E-05 (Males) |
| Tm_14091 | CYP3 clan | 6.63E-01 | 1.68E-01 | 6.50E-01 |
| Tm_12854 | CYP3 clan | 7.26E-01 | 8.25E-02 | 3.93E-01 |
| Tm_01372 | CYP3 clan | 5.42E-01 | 4.00E-01 | 8.25E-01 |
| Tm_07853 | CYP3 clan | 2.69E-01 | 1.31E-02 (Females) | 2.03E-05 (Males) |
| Tm_09891 | CYP3 clan | 5.62E-01 | 3.74E-02 (Females) | 6.81E-03 (Males) |
| Tm_07004 | CYP3 clan | 1.25E-04 (Females) | 3.13E-02 (Females) | 3.64E-01 |
| Tm_03434 | CYP3 clan | 7.97E-01 | 4.65E-01 | 4.86E-01 |
| Tm_09150 | CYP3 clan | 7.53E-01 | 3.13E-02 (Females) | 8.49E-02 |
| Tm_14705 | CYP3 clan | 9.77E-01 | 6.49E-01 | 6.30E-01 |
| Tm_15190 | CYP3 clan | 5.97E-01 | 6.33E-01 | 4.06E-01 |
| Tm_01157 | CYP4 clan | 7.85E-04 (Females) | 4.37E-02 (Females) | 6.67E-01 |
| Tm_06697 | CYP4 clan | 8.19E-01 | 1.23E-04 (Females) | 2.24E-05 (Males) |
| Tm_11223 | CYP4 clan | 9.38E-01 | 1.10E-01 | 1.08E-01 |
| Tm_05131 | CYP4 clan | 3.99E-02 (Females) | 4.27E-01 | 5.66E-01 |
| Tm_01415 | CYP4 clan | 8.19E-01 | 1.37E-04 (Females) | 1.27E-05 (Males) |
| Tm_01436 | CYP4 clan | 3.90E-01 | 9.81E-05 (Females) | 1.72E-06 (Males) |
| Tm_06642 | CYP4 clan | 6.34E-01 | 7.46E-05 (Females) | 8.93E-04 (Males) |
| Tm_03283 | CYP4 clan | 7.98E-01 | 6.18E-02 | 1.30E-01 |
| Tm_02528 | CYP4 clan | 4.25E-01 | 9.12E-02 (Females) | 1.36E-02 (Males) |
| Tm_02530 | CYP4 clan | 5.06E-01 | 1.30E-01 | 7.52E-03 (Males) |
| Tm_01583 | CYP4 clan | 1.59E-01 | 8.16E-04 (Nymphs) | 1.12E-01 |
| Tm_00300 | CYP4 clan | 4.55E-01 | 2.98E-01 | 4.88E-02 (Males) |
| Tm_01048 | CYP4 clan | - | - | 1.00E+00 |
| Tm_05802 | CYP4 clan | 2.98E-01 | 1.00E+00 | 5.99E-01 |
| Tm_15191 | CYP4 clan | 4.35E-01 | 1.30E-02 (Females) | 1.33E-01 |
| Tm_10252 | Mito. Clan | 2.10E-01 | 3.68E-06 (Nymphs) | 1.12E-05 (Nymphs) |
| Tm_11316 | Mito. Clan | - | 1.05E-06 (Nymphs) | 2.82E-06 (Nymphs) |
| Tm_13084 | Mito. Clan | 9.20E-01 | 9.62E-01 | 8.78E-01 |
| Tm_11295 | Mito. Clan | 4.38E-01 | 5.00E-01 | 9.51E-01 |
| Tm_07765 | Mito. Clan | 9.60E-08 (Females) | 2.06E-01 | 1.12E-03 (Females) |

The results are listed as the FDR value. Gray background highlights the FDR values to show the corresponding genes are statistically overrepresented in pair-wise comparisons (FDR *P*-value < 0.05).

Supplementary Table 15. Characterization of *T. mercedesae* GST genes.

| **Gene ID** | **GST subfamily** | **Size (aa)** | **Expression** | **Notes** |
| --- | --- | --- | --- | --- |
| Tm_15207 | Delta | 50 | no | Internal stop codon |
| Tm_15206 | Delta | 80 | no | Short fragment |
| Tm_15204 | Delta | 214 | yes | Complete |
| Tm_09167 | Delta | 180 | no | Incompleted C-terminal |
| Tm_07535 | Delta | 218 | yes | Complete |
| Tm_12349 | kappa | 224 | yes | Complete |
| Tm_15205 | mu | 223 | yes | Complete |
| Tm_15203 | mu | 116 | no | Internal stop codon |
| Tm_15202 | mu | 199 | no | Internal stop codon |
| Tm_15201 | mu | 139 | no | Internal stop codon |
| Tm_07777 | mu | 72 | no | Short fragment |
| Tm_03887 | mu | 238 | yes | Complete |
| Tm_06475 | Omega | 238 | yes | Complete |
| Tm_06358 | Omega | 241 | yes | Complete |
| Tm_05455 | Unknown | 84 | no | Short fragment |

If a GST gene contains more than one read per million in the replicates, it is considered to be expressed and labeled ‘yes’, and if not, labeled ‘no’. The ‘fragment’ and ‘complete’ indicates the GST proteins with less than 100 amino acids and with both intact N- and C-terminal domains, respectively. The unexpressed and fragmented GST genes and those with stop codons are considered as pseudogenes highlighted with gray background.

Supplementary Table 16. The number of GST subfamily members in different animal species.

| **GST subfamily** | **alpha** | **delta** | **epsilon** | **mu** | **pi** | **omega** | **sigma** | **theta** | **zeta** | **kappa** | **unknown** | **Total** |
| --- | --- | --- | --- | --- | --- | --- | --- | --- | --- | --- | --- | --- |
| ***T. mercedesae*** | 0 | 5 | 0 | 6 | 0 | 2 | 0 | 0 | 0 | 1 | 1 | 15 |
| ***M. occidentalis*** | 0 | 3 | 0 | 6 (5) | 0 | 3 | 0 | 0 | 0 (1) | 2 | 1 | 15 |
| ***I. scapularis*** | 0 | 7 | 5 | 14 | 0 | 3 | 0 | 0 | 3 | 2 | 0 | 34 |
| ***T. urticae*** | 0 | 16 | 0 | 12 | 0 | 2 | 0 | 1 | 0 | 1 | 0 | 32 |
| ***D. melanogaster*** | 0 | 11 | 14 | 0 | 0 | 5 | 1 | 4 | 2 | 0 | 0 | 37 |
| ***A. gambiae*** | 0 | 12 | 8 | 0 | 0 | 1 | 1 | 2 | 1 | 0 | 3 | 28 |
| ***A. mellifera*** | 0 | 1 | 0 | 0 | 0 | 1 | 4 | 1 | 1 | 0 | 0 | 8 |
| ***N. vitripennis*** | 0 | 5 | 0 | 0 | 0 | 2 | 8 | 3 | 1 | 0 | 0 | 19 |
| ***T. castaneum*** | 0 | 3 | 19 | 0 | 0 | 4 | 7 | 1 | 1 | 0 | 0 | 35 |
| ***B. mori*** | 0 | 4 | 8 | 0 | 0 | 4 | 2 | 1 | 2 | 0 | 2 | 23 |
| ***H. sapiens*** | 5 | 0 | 0 | 5 | 1 | 2 | 1 | 2 | 1 | 1 | 0 | 18 |

*Anopheles gambiae, Nasonia vitripennis, Tribolium castaneum,* and *Homo sapiens* are included for the comparison. The numbers for each species are derived from previous reports [2-5] and this study. Parentheses indicate the numbers of subfamily members in *M. occidentalis* identified by [4].

Supplementary Table 17. Characterization of *T. mercedesae* CCE genes.

| **Gene ID** | **CCE subfamily** | **Size (aa)** | **Expression** | **Notes** |
| --- | --- | --- | --- | --- |
| Tm_00126 | Clade G | 594 | yes | Complete |
| Tm_00699 | Clade J | 346 | yes | Incompleted N-terminal |
| Tm_00745 | Clade J | 327 | yes | Incompleted C-terminal |
| Tm_01590 | Clade J | 475 | no | Complete |
| Tm_02203 | Clade J | 234 | yes | Incompleted C-terminal |
| Tm_02327 | Clade J | 591 | yes | Complete |
| Tm_02390 | Clade J | 516 | yes | Complete |
| Tm_04344 | Clade J | 184 | yes | Incompleted C-terminal |
| Tm_05109 | Clade J | 295 | yes | Incompleted N-terminal |
| Tm_06412 | Clade J | 1503 | yes | Complete |
| Tm_08305 | Clade J | 689 | yes | Complete |
| Tm_08415 | Clade J | 166 | no | Short fragment |
| Tm_09468 | Clade J | 677 | yes | Complete |
| Tm_09507 | Clade J | 438 | yes | Incompleted C-terminal |
| Tm_10069 | Clade J | 563 | yes | Complete |
| Tm_10626 | Clade J | 627 | yes | Complete |
| Tm_10790 | Clade J | 321 | yes | Incompleted N-terminal |
| Tm_11238 | Clade J | 577 | yes | Complete |
| Tm_11273 | Clade J | 599 | yes | Complete |
| Tm_11965 | Clade J | 468 | yes | Complete |
| Tm_12088 | Clade J | 286 | yes | Incompleted C-terminal |
| Tm_12621 | Clade J | 286 | yes | Incompleted C-terminal |
| Tm_12914 | Clade J | 561 | yes | Complete |
| Tm_13989 | Clade J | 548 | yes | Complete |
| Tm_13990 | Clade J | 548 | yes | Complete |
| Tm_13991 | Clade J | 551 | yes | Complete |
| Tm_15253 | Clade J | 50 | no | Short fragment |
| Tm_15254 | Clade J | 69 | no | Short fragment |
| Tm_15250 | Clade J | 124 | no | Internal stop codon |
| Tm_15251 | Clade J | 135 | no | Internal stop codon |
| Tm_15252 | Clade J | 210 | no | Internal stop codon |
| Tm_05721 | Clade L | 773 | yes | Complete |
| Tm_01355 | Clade M | 650 | yes | Complete |
| Tm_01458 | Clade M | 1053 | yes | Complete |
| Tm_01522 | Clade M | 619 | yes | Complete |
| Tm_01778 | Clade M | 176 | yes | Incompleted C-terminal |
| Tm_01875 | Clade M | 199 | yes | Incompleted C-terminal |
| Tm_01913 | Clade M | 168 | no | Incompleted C-terminal |
| Tm_01918 | Clade M | 145 | no | Short fragment |
| Tm_04413 | Clade M | 104 | no | Short fragment |
| Tm_05618 | Clade M | 695 | yes | Complete |
| Tm_06192 | Clade M | 597 | yes | Complete |
| Tm_11821 | Clade M | 238 | yes | Incompleted N-terminal |
| Tm_11953 | Clade M | 51 | no | Short fragment |
| Tm_12394 | Clade M | 221 | no | Incompleted N-terminal |
| Tm_12528 | Clade M | 1036 | yes | Complete |
| Tm_12928 | Clade M | 772 | yes | Incompleted N-terminal |
| Tm_13352 | Clade M | 890 | yes | Complete |
| Tm_14715 | Clade M | 158 | no | Incompleted N-terminal |
| Tm_14639 | Clade M | 108 | no | Short fragment |
| Tm_04770 | Undetermined | 72 | no | Short fragment |

If a CCE gene contains more than one read per million in the replicates, it is considered to be expressed and labeled ‘yes’, and if not, labeled ‘no’. The ‘fragment’ and ‘complete’ indicate the CCE protein with less than 150 amino acids and the complete CO esterase domain (PF00135), respectively. The unexpressed and fragmented CCE genes and those with stop codons are considered as pseudogenes highlighted with gray background.

Supplementary Table 18. The number of CCE subfamily members in different arthropod species.

| **CCE subfamily** | ***T. mercedesae*** | ***M. occidentalis*** | ***D. melanogaster*** | ***A. gambiae*** | ***A. mellifera*** | ***B. mori*** |
| --- | --- | --- | --- | --- | --- | --- |
| **Dietary class** |  |  |  |  |  |  |
| Clade A | 0 | 0 | 1 | 0 | 0 | 42 |
| Clade B | 0 | 0 | 12 | 0 | 8 | 13 |
| Clade C | 0 | 0 | 0 | 16 | 0 | 0 |
| **Hormone/semiochemical processing** |  |  |  |  |  |  |
| Clade D (Integument esterase) | 0 | 0 | 3 | 4 | 1 | 4 |
| Clade E (Secreted β-esterase) | 0 | 0 | 2 | 0 | 1 | 2 |
| Clade F (Dipteran JhE) | 0 | 0 | 3 | 5 | 0 | 0 |
| Clade G (Lepidopteran JhE) | 1 | 1 (0) | 0 | 5 | 3 | 2 |
| **Neuro/development** |  |  |  |  |  |  |
| Clade H (Glutactin) | 0 | 0 | 1 | 1 | 1 | 1 |
| Clade I (Uncharacterized clade) | 0 | 0 | 4 | 9 | 0 | 0 |
| Clade J (Acetylcholinesterases) | 29 | 36 (35) | 1 | 2 | 2 | 2 |
| Clade K (Gliotactin) | 0 | 0 (1) | 1 | 1 | 1 | 1 |
| Clade L (Neuroligins) | 1 | 1 (5) | 1 | 1 | 1 | 1 |
| Clade M (Neurotactins) | 18 | 6 (0) | 4 | 5 | 5 | 6 |
| Clade N (Neurotactin) | 0 | 0 | 2 | 2 | 1 | 2 |
| **Other** | 2 | 3 | 0 | 0 | 0 | 0 |
| **Total** | 50 | 47 (44) | 35 | 51 | 24 | 76 |

The numbers for each species are derived from the previous report [4, 6] and this study. Parentheses indicate the numbers of subfamily members in *M. occidentalis* identified by [4].

Supplememtary Table 19. Pairwise comparisons of mRNA levels of *T. mercedesae* CCE between females, males, and nymphs.

| **Gene ID** | **GST subfamily** | **Pairwise comparisons of mRNA expression** | | |
| --- | --- | --- | --- | --- |
|  |  | **Females vs. Males** | **Females vs. Nymphs** | **Males vs. Nymphs** |
| Tm_00126 | Clade G | 8.91E-01 | 1.33E-01 | 1.26E-01 |
| Tm_00699 | Clade J | 6.75E-01 | 8.96E-01 | 9.99E-01 |
| Tm_00745 | Clade J | 3.50E-03 (Males) | 8.54E-01 | 2.38E-02 (Males) |
| Tm_02203 | Clade J | 3.62E-10 (Males) | 5.02E-05 (Nymphs) | 1.36E-01 |
| Tm_02327 | Clade J | 1.25E-01 | 3.83E-01 | 9.80E-01 |
| Tm_02390 | Clade J | - | 2.83E-17 (Nymphs) | 2.64E-16 (Nymphs) |
| Tm_05109 | Clade J | 5.31E-01 | 1.62E-01 | 3.53E-02 (Males) |
| Tm_06412 | Clade J | 4.19E-02 (Males) | 4.08E-01 | 7.75E-01 |
| Tm_08305 | Clade J | 1.00E+00 | 4.88E-01 | 6.52E-01 |
| Tm_09468 | Clade J | 1.14E-01 | 5.02E-01 | 9.03E-01 |
| Tm_09507 | Clade J | - | 2.62E-13 (Nymphs) | 4.06E-10 (Nymphs) |
| Tm_10069 | Clade J | 2.02E-01 | 3.53E-03 (Nymphs) | 4.03E-02 (Nymphs) |
| Tm_10626 | Clade J | 3.05E-03 (Males) | 1.59E-01 | 6.22E-05 (Males) |
| Tm_10790 | Clade J | 1.21E-13 (Males) | 1.95E-08 (Nymphs) | 4.34E-01 |
| Tm_11238 | Clade J | 6.14E-01 | 6.57E-02 | 4.87E-03 (Males) |
| Tm_11273 | Clade J | 2.29E-01 | 2.64E-02 (Nymphs) | 3.76E-01 |
| Tm_11965 | Clade J | - | 3.87E-03 (Nymphs) | 1.74E-02 (Nymphs) |
| Tm_12088 | Clade J | 7.26E-01 | 8.24E-01 | 8.06E-01 |
| Tm_12621 | Clade J | 8.24E-01 | 1.00E+00 | 9.76E-01 |
| Tm_12914 | Clade J | 3.60E-01 | 1.55E-01 | 2.14E-03 (Males) |
| Tm_13989 | Clade J | 4.66E-01 | 7.38E-02 | 5.90E-01 |
| Tm_13990 | Clade J | 6.14E-01 | - | - |
| Tm_13991 | Clade J | 1.59E-01 | 4.36E-01 | 4.72E-02 (Males) |
| Tm_05721 | Clade L | 1.89E-01 | 7.88E-01 | 2.30E-01 |
| Tm_01355 | Clade M | 6.65E-01 | 9.14E-01 | 9.79E-01 |
| Tm_01458 | Clade M | 2.67E-01 | 9.25E-02 | 5.84E-01 |
| Tm_01522 | Clade M | 1.68E-03 (Females) | 9.25E-07 (Nymphs) | 1.40E-13 (Nymphs) |
| Tm_01778 | Clade M | - | 1.24E-01 | 5.88E-01 |
| Tm_01875 | Clade M | - | 1.81E-01 | 7.24E-01 |
| Tm_05618 | Clade M | 9.92E-02 | 1.56E-01 | 9.74E-01 |
| Tm_06192 | Clade M | 2.99E-04 (Males) | 6.88E-02 | 5.87E-01 |
| Tm_11821 | Clade M | 2.42E-01 | 2.48E-01 | 9.55E-01 |
| Tm_12528 | Clade M | 4.77E-02 (Males) | 3.96E-02 (Nymphs) | 9.54E-01 |
| Tm_12928 | Clade M | 5.37E-01 | 2.36E-01 | 6.94E-01 |
| Tm_13352 | Clade M | 1.87E-04 (Males) | 3.08E-02 (Females) | 1.27E-09 (Males) |

The results are listed as the FDR value. Gray background highlights the FDR values to show the corresponding genes are statistically overrepresented in pair-wise comparisons (FDR *P*-value < 0.05).

Supplememtary Table 20 The number of ABC transporter subfamily members in different animal species.

| **ABC transporter subfamily** | **A** | **B** | **C** | **D** | **E** | **F** | **G** | **H** | **Total** |
| --- | --- | --- | --- | --- | --- | --- | --- | --- | --- |
| ***T. mercedesae*** | 12 | 4 | 22 | 3 | 2 | 4 | 2 | 3 | 52 |
| ***M. occidentalis*** | 10 | 7 | 22 | 5 | 2 | 3 | 2 | 6 | 57 |
| ***T. urticae*** | 9 | 4 | 39 | 2 | 1 | 3 | 23 | 22 | 103 |
| ***D. melanogaster*** | 10 | 8 | 14 | 2 | 1 | 3 | 15 | 3 | 56 |
| ***A. gambiae*** | 9 | 5 | 13 | 2 | 1 | 3 | 16 | 3 | 52 |
| ***T. castaneum*** | 10 | 6 | 35 | 2 | 1 | 3 | 13 | 3 | 73 |
| ***A. mellifera*** | 3 | 5 | 9 | 2 | 1 | 3 | 15 | 3 | 41 |
| ***B. mori*** | 9 | 9 | 15 | 2 | 1 | 3 | 13 | 3 | 55 |
| ***D. pulex*** | 4 | 7 | 7 | 3 | 1 | 4 | 24 | 15 | 65 |
| ***H. sapiens*** | 12 | 11 | 12 | 4 | 1 | 3 | 5 | 0 | 48 |

The numbers for each species are derived from the previous report [7] and this study.

Supplememtary Table 21. Pairwise comparisons of mRNA levels of *T. mercedesae* ABC transporter between females, males, and nymphs.

| **Gene ID** | **ABC transporter subfamily** | **Pairwise comparisons of mRNA expression** | | |
| --- | --- | --- | --- | --- |
|  |  | **Females vs Males** | **Females vs Nymphs** | **Males vs Nymphs** |
| Tm_00961 | A | 8.25E-01 | 1.54E-01 | 2.29E-01 |
| Tm_01003 | A | 5.46E-08 (Males) | 5.52E-02 | 5.32E-03 (Males) |
| Tm_03938 | A | 1.25E-09 (Males) | 9.47E-04 (Nymphs) | 4.24E-02 (Males) |
| Tm_04193 | A | 9.59E-01 | 6.60E-01 | 5.86E-01 |
| Tm_04648 | A | 3.68E-01 | 6.46E-01 | 6.67E-02 |
| Tm_10895 | A | 7.29E-01 | 1.00E+00 | 9.60E-01 |
| Tm_13010 | A | 9.26E-01 | 8.90E-01 | 9.30E-01 |
| Tm_14342 | A | 5.67E-01 | 2.38E-01 | 2.79E-03 (Males) |
| Tm_14841 | A | 9.65E-06 (Males) | - | 1.29E-01 |
| Tm_00814 | B | 3.54E-01 | 9.78E-01 | 4.43E-01 |
| Tm_05598 | B | 6.77E-01 | 5.62E-01 | 8.80E-01 |
| Tm_06198 | B | 3.05E-01 | 8.63E-01 | 8.33E-01 |
| Tm_07428 | B | 9.92E-01 | 8.64E-01 | 9.90E-01 |
| Tm_00971 | C | 1.56E-22 (Males) | 2.59E-04 (Nymphs) | 3.75E-07 (Males) |
| Tm_00978 | C | 1.00E+00 | 5.45E-01 | 8.04E-01 |
| Tm_02164 | C | 8.66E-03 (Males) | 1.45E-03 (Nymphs) | 6.63E-01 |
| Tm_02321 | C | 2.54E-16 (Males) | 2.65E-04 (Nymphs) | 4.41E-04 (Males) |
| Tm_04002 | C | 2.37E-01 | 6.34E-01 | 9.42E-02 |
| Tm_04067 | C | 1.12E-01 | 5.74E-03 (Nymphs) | 2.87E-01 |
| Tm_04154 | C | 5.30E-01 | 2.84E-01 | 6.12E-02 |
| Tm_04250 | C | 1.17E-02 (Males) | 1.46E-01 | 8.75E-01 |
| Tm_04697 | C | 6.66E-01 | 5.25E-01 | 1.00E+00 |
| Tm_04724 | C | 1.08E-01 | 1.12E-01 | 9.69E-01 |
| Tm_07089 | C | 7.30E-01 | 4.21E-01 | 6.99E-01 |
| Tm_07412 | C | 9.70E-01 | 6.71E-01 | 6.32E-01 |
| Tm_07445 | C | 9.30E-01 | 4.63E-01 | 4.06E-01 |
| Tm_07924 | C | 5.26E-01 | 1.92E-07 (Females) | 7.20E-06 (Males) |
| Tm_08179 | C | 3.96E-01 | 5.73E-01 | 1.00E+00 |
| Tm_08441 | C | 5.58E-01 | 5.23E-01 | 1.00E+00 |
| Tm_08755 | C | 3.38E-01 | 1.68E-01 | 6.90E-01 |
| Tm_09065 | C | 9.47E-01 | 8.20E-01 | 8.62E-01 |
| Tm_10611 | C | 2.34E-02 (Females) | 2.16E-01 | 6.95E-01 |
| Tm_13528 | C | 8.73E-01 | 9.92E-01 | 9.14E-01 |
| Tm_01291 | D | 4.66E-01 | 5.10E-01 | 9.82E-01 |
| Tm_01871 | D | 6.86E-01 | 9.35E-01 | 8.66E-01 |
| Tm_10839 | D | 9.19E-01 | 4.38E-01 | 5.24E-01 |
| Tm_12283 | E | 8.53E-01 | 4.92E-01 | 4.36E-01 |
| Tm_08514 | E | - | 6.11E-01 | 1.00E+00 |
| Tm_08954 | F | 6.69E-01 | - | - |
| Tm_01491 | F | 6.58E-01 | 4.71E-01 | 9.71E-01 |
| Tm_02302 | F | 7.88E-01 | 6.36E-01 | 9.93E-01 |
| Tm_03083 | F | 6.92E-01 | 3.43E-01 | 8.51E-01 |
| Tm_04514 | G | 4.33E-01 | 7.20E-07 (Nymphs) | 7.34E-04 (Nymphs) |
| Tm_05535 | G | 8.44E-02 | 3.89E-03 (Nymphs) | 2.84E-01 |
| Tm_03284 | H | 1.79E-05 (Males) | - | 3.13E-03 (Males) |
| Tm_07117 | H | 5.03E-02 | 2.45E-03 (Nymphs) | 2.53E-01 |
| Tm_07059 | unclassfied | 2.89E-10 (Males) | - | 2.36E-05 (Males) |

The results are listed as the FDR. Gray background highlights the FDR to show the corresponding genes are statistically overrepresented in pair-wise comparisons (FDR *P*-value < 0.05).

Supplememtary Table 22. The numbers of *transformer-2*, *dmrt,* and *intersex* genes in various arthropods.

| **Species** | ***transformer-2*** | ***dmrt*** | ***intersex*** |
| --- | --- | --- | --- |
| ***T. mercedesae*** | 1 | 7 | 1 |
| ***M. occidentalis*** | 1 | 4 | 1 |
| ***I. scapularis*** | 1 | 5 | 1 |
| ***D. melanogaster*** | 1 | 4 | 1 |
| ***B. mori*** | 1 | 4 | 1 |
| ***A. mellifera*** | 1 | 4 | 1 |
| ***T. castaneum*** | 1 | 3 | 1 |
| ***D. magna*** | 1 | 5 | 1 |

*Daphnia magna* is included for the comparison. The numbers for each species are derived from the previous report [8] and this study.

Supplementary Table 23. Gene families differentially expressed (DE) in the adult males and females

| **Gene family** | **Gene family size** | **Number of DE proteins** | **Individuals with DE** | | ***P*-value** | **Description** |
| --- | --- | --- | --- | --- | --- | --- |
|  |  |  | **Males** | **Females** |  |  |
| ORTHOMCL1 | 251 | 69 | 50 | 19 | 0.00E+00 | Serine threonine protein |
| ORTHOMCL5 | 72 | 28 | 22 | 6 | 4.73E-10 | Serine protease |
| ORTHOMCL44 | 23 | 13 | 10 | 3 | 1.63E-06 | Serine threonine-protein phosphatase |
| ORTHOMCL67 | 17 | 7 | 3 | 4 | 8.25E-04 | Uncharacterized protein |
| ORTHOMCL72 | 16 | 6 | 6 | 0 | 5.68E-04 | Chitinase |
| ORTHOMCL97 | 13 | 6 | 6 | 0 | 1.47E-04 | Insulin-degrading enzyme |
| ORTHOMCL111 | 12 | 6 | 4 | 2 | 9.62E-04 | Cathepsin |
| ORTHOMCL112 | 12 | 9 | 1 | 8 | 8.61E-09 | Importin |
| ORTHOMCL128 | 11 | 9 | 9 | 0 | 2.30E-09 | Pi-plc x domain-containing protein |
| ORTHOMCL143 | 10 | 5 | 5 | 0 | 3.46E-04 | Uncharacterized protein |
| ORTHOMCL146 | 10 | 6 | 6 | 0 | 2.17E-05 | Histone-lysine n-methyltransferase |
| ORTHOMCL162 | 9 | 6 | 6 | 0 | 1.84E-04 | Sodium-dependent phosphate transporter |
| ORTHOMCL181 | 8 | 5 | 0 | 5 | 8.67E-05 | Cyclin |
| ORTHOMCL221 | 7 | 4 | 4 | 0 | 7.66E-04 | Protein lifeguard |
| ORTHOMCL257 | 6 | 5 | 0 | 5 | 1.05E-05 | Uncharacterized protein |
| ORTHOMCL259 | 6 | 4 | 0 | 4 | 3.48E-04 | Vitellogenin |
| ORTHOMCL264 | 6 | 5 | 5 | 0 | 3.48E-04 | N-acetyltransferase gcn5 |
| ORTHOMCL275 | 5 | 4 | 0 | 4 | 1.23E-04 | Dna replication licensing factor |
| ORTHOMCL336 | 5 | 5 | 5 | 0 | 1.86E-06 | Protein fam188b |
| ORTHOMCL338 | 5 | 5 | 4 | 1 | 1.86E-06 | Uncharacterized protein |
| ORTHOMCL442 | 4 | 4 | 0 | 4 | 2.61E-05 | Uncharacterized protein |
| ORTHOMCL443 | 4 | 4 | 4 | 0 | 2.61E-05 | Uncharacterized protein |
| ORTHOMCL456 | 4 | 4 | 4 | 0 | 2.61E-05 | Uncharacterized protein |
| ORTHOMCL684 | 3 | 3 | 3 | 0 | 3.66E-04 | Uncharacterized protein |
| ORTHOMCL686 | 3 | 3 | 3 | 0 | 3.66E-04 | Uncharacterized protein |
| ORTHOMCL687 | 3 | 3 | 1 | 2 | 3.66E-04 | Uncharacterized protein |
| ORTHOMCL691 | 3 | 3 | 3 | 0 | 3.66E-04 | Uncharacterized protein |
| ORTHOMCL692 | 3 | 3 | 0 | 3 | 3.66E-04 | Uncharacterized protein |
| ORTHOMCL694 | 3 | 3 | 3 | 0 | 3.66E-04 | Type 11 methyltransferase |
| ORTHOMCL701 | 3 | 3 | 0 | 3 | 3.66E-04 | Nanos-like protein |
| ORTHOMCL702 | 3 | 1 | 0 | 1 | 3.66E-04 | Uncharacterized protein |
| ORTHOMCL706 | 3 | 3 | 3 | 0 | 3.66E-04 | Uncharacterized protein |
| ORTHOMCL711 | 3 | 3 | 3 | 0 | 3.66E-04 | Uncharacterized protein |

The statistical analysis was conducted by Fisher’s extract test (*P*-value < 0.001).

Supplementary Table 24. Gene families differentially expressed (DE) in the adult females and nymphs.

| **Gene family** | **Gene family size** | **Number of DE proteins** | **Individuals with DE** | | ***P*-value** | **Descirption** |
| --- | --- | --- | --- | --- | --- | --- |
|  |  |  | Females | Nymphs |  |  |
| ORTHOMCL1 | 251 | 45 | 17 | 28 | 2.88E-08 | Serine threonine protein |
| ORTHOMCL2 | 125 | 61 | 15 | 46 | 2.50E-13 | Cuticle protein |
| ORTHOMCL5 | 72 | 22 | 5 | 17 | 2.49E-09 | Serine protease |
| ORTHOMCL15 | 44 | 23 | 5 | 18 | 7.83E-05 | Epidermal growth factor-related receptor |
| ORTHOMCL20 | 35 | 12 | 2 | 10 | 4.80E-05 | Elongation of very long chain fatty acids protein |
| ORTHOMCL22 | 34 | 14 | 3 | 11 | 2.19E-04 | Nose resistant to fluoxetine protein |
| ORTHOMCL26 | 29 | 18 | 0 | 18 | 5.83E-10 | Protocadherin fat |
| ORTHOMCL37 | 24 | 12 | 0 | 12 | 5.82E-04 | Voltage-dependent calcium channel |
| ORTHOMCL38 | 24 | 14 | 1 | 13 | 6.09E-09 | Chitin binding domain containing protein |
| ORTHOMCL52 | 20 | 9 | 3 | 6 | 1.58E-06 | Tubulin alpha |
| ORTHOMCL57 | 19 | 14 | 6 | 8 | 5.56E-12 | Uncharacterized protein |
| ORTHOMCL67 | 17 | 10 | 4 | 6 | 2.74E-07 | Uncharacterized protein |
| ORTHOMCL72 | 16 | 9 | 0 | 9 | 2.28E-06 | Chitinase |
| ORTHOMCL85 | 14 | 12 | 0 | 12 | 2.22E-11 | Uncharacterized protein |
| ORTHOMCL105 | 12 | 7 | 0 | 7 | 2.63E-06 | Uncharacterized protein |
| ORTHOMCL106 | 12 | 10 | 0 | 10 | 6.73E-11 | Uncharacterized protein |
| ORTHOMCL112 | 12 | 7 | 7 | 0 | 2.63E-06 | Importin subunit |
| ORTHOMCL164 | 8 | 5 | 3 | 2 | 9.62E-04 | Uncharacterized protein |
| ORTHOMCL177 | 8 | 5 | 0 | 5 | 9.62E-04 | Netrin receptor |
| ORTHOMCL181 | 8 | 6 | 6 | 0 | 1.74E-06 | Cyclin |
| ORTHOMCL193 | 8 | 5 | 2 | 3 | 5.16E-05 | Glucose dehydrogenase |
| ORTHOMCL214 | 7 | 5 | 0 | 5 | 5.07E-04 | Uncharacterized protein |
| ORTHOMCL219 | 7 | 4 | 4 | 0 | 5.07E-04 | Eukaryotic translation initiation factor |
| ORTHOMCL257 | 6 | 5 | 5 | 0 | 2.29E-04 | Uncharacterized protein |
| ORTHOMCL259 | 6 | 5 | 5 | 0 | 6.16E-06 | Vitellogenin |
| ORTHOMCL266 | 6 | 4 | 4 | 0 | 2.29E-04 | Zinc finger protein |
| ORTHOMCL320 | 5 | 5 | 0 | 5 | 1.08E-06 | Uncharacterized protein |
| ORTHOMCL338 | 5 | 4 | 1 | 3 | 8.04E-05 | Uncharacterized protein |
| ORTHOMCL442 | 4 | 4 | 4 | 0 | 1.69E-05 | Uncharacterized protein |
| ORTHOMCL660 | 3 | 3 | 0 | 3 | 2.65E-04 | Uncharacterized protein |
| ORTHOMCL687 | 3 | 3 | 3 | 0 | 2.65E-04 | Uncharacterized protein |
| ORTHOMCL692 | 3 | 3 | 3 | 0 | 2.65E-04 | Uncharacterized protein |
| ORTHOMCL698 | 3 | 3 | 3 | 0 | 2.65E-04 | Uncharacterized protein |
| ORTHOMCL701 | 3 | 3 | 3 | 0 | 2.65E-04 | Uncharacterized protein |
| ORTHOMCL702 | 3 | 1 | 1 | 0 | 2.65E-04 | Uncharacterized protein |

The statistical analysis was conducted by Fisher’s extract test (*P*-value < 0.001).

Supplementary Table 25. The enriched GO terms associated with genes highly expressed in the nymphs compared to adult females.

| **GO Term** | **Description** | **Type** | ***P*-value** |
| --- | --- | --- | --- |
| GO:0042302 | Structural constituent of cuticle | F | 9.30E-18 |
| GO:0008061 | Chitin binding | F | 6.63E-09 |
| GO:0006030 | Chitin metabolic process | P | 1.18E-08 |
| GO:1901071 | Glucosamine-containing compound metabolic process | P | 1.68E-08 |
| GO:0006040 | Amino sugar metabolic process | P | 1.98E-08 |
| GO:0007156 | Homophilic cell adhesion via plasma membrane adhesion molecules | P | 1.09E-07 |
| GO:0005198 | Structural molecule activity | F | 1.60E-07 |
| GO:0098742 | Cell-cell adhesion via plasma-membrane adhesion molecules | P | 2.31E-07 |
| GO:0006022 | Aminoglycan metabolic process | P | 3.03E-06 |
| GO:0005576 | Extracellular region | C | 2.20E-04 |
| GO:0098609 | Cell-cell adhesion | P | 6.32E-04 |
| GO:0022610 | Biological adhesion | P | 8.13E-04 |
| GO:0007155 | Cell adhesion | P | 8.13E-04 |
| GO:0007552 | Metamorphosis | P | 8.13E-04 |
| GO:0004252 | Serine-type endopeptidase activity | F | 1.04E-03 |
| GO:0008233 | Peptidase activity | F | 1.04E-03 |
| GO:0005509 | Calcium ion binding | F | 1.13E-03 |
| GO:0048569 | Post-embryonic organ development | P | 1.13E-03 |
| GO:0007476 | Imaginal disc-derived wing morphogenesis | P | 1.13E-03 |
| GO:0035114 | Imaginal disc-derived appendage morphogenesis | P | 1.13E-03 |
| GO:0007469 | Antennal development | P | 1.21E-03 |
| GO:0007472 | Wing disc morphogenesis | P | 1.31E-03 |
| GO:0007560 | Imaginal disc morphogenesis | P | 1.31E-03 |
| GO:0048737 | Imaginal disc-derived appendage development | P | 1.31E-03 |
| GO:0048563 | Post-embryonic organ morphogenesis | P | 1.68E-03 |
| GO:0070011 | Peptidase activity, acting on L-amino acid peptides | F | 2.57E-03 |
| GO:0002165 | Instar larval or pupal development | P | 2.60E-03 |
| GO:0050954 | Sensory perception of mechanical stimulus | P | 2.79E-03 |
| GO:0035120 | Post-embryonic appendage morphogenesis | P | 2.88E-03 |
| GO:0009791 | Post-embryonic development | P | 3.24E-03 |
| GO:0048707 | Instar larval or pupal morphogenesis | P | 3.63E-03 |
| GO:0004175 | Endopeptidase activity | F | 3.63E-03 |
| GO:0048800 | Antennal morphogenesis | P | 3.63E-03 |
| GO:0017171 | Serine hydrolase activity | F | 4.26E-03 |
| GO:0008236 | Serine-type peptidase activity | F | 4.26E-03 |
| GO:0035316 | Non-sensory hair organization | P | 6.20E-03 |
| GO:0035317 | Imaginal disc-derived wing hair organization | P | 6.20E-03 |
| GO:0035220 | Wing disc development | P | 7.21E-03 |
| GO:0009886 | Post-embryonic morphogenesis | P | 8.22E-03 |
| GO:0030048 | Actin filament-based movement | P | 8.93E-03 |

The corrected FDR *P*-value (< 0.01) was calculated by Fisher’s extract test embedded in the Blast2go software desktop version.

Supplementary Table 26. The expected counts and expression levels (FPKM) of RNA-seq reads that map to *de novo* assembled DWV RNA. The E-value and sequence description are from the best hit by blastn.

| **Sample** | **Transcript_ID** | **Size (bp)** | **Expected count** | **FPKM** | **E-value** | **Sequence description** |
| --- | --- | --- | --- | --- | --- | --- |
| **Adult males #1** | comp17080_c0_seq1 | 10,164 | 18,981,836.85 | 84920.97 | 0.00E+00 | Kakugo virus genomic RNA, complete genome |
| **Adult males #2** | comp22899_c0_seq1 | 268 | 266.32 | 219.34 | 4.00E-114 | Deformed wing virus isolate POGTM1 lp gene, partial cds |
|  | comp22899_c0_seq2 | 268 | 340.68 | 280.58 | 8.00E-110 | Deformed wing virus isolate Tc-P12alt polyprotein gene, partial cds |
|  | comp22899_c0_seq3 | 268 | 470 | 387.09 | 8.00E-110 | Deformed wing virus isolate JP6 polyprotein gene, partial cds |
|  | comp25235_c0_seq1 | 10,134 | 6,545,519.08 | 54,999.49 | 0.00E+00 | Kakugo virus genomic RNA, complete genome |
| **Adult female #1** | comp11954_c1_seq1 | 261 | 2504.03 | 549.08 | 1.00E-117 | Deformed wing virus isolate Tc-K21 polyprotein gene, partial cds |
|  | comp13227_c0_seq1 | 10,143 | 16,537,062.23 | 73,810 | 0.00E+00 | Kakugo virus, complete genome |
| **Adult female #2** | comp40733_c0_seq1 | 5,518 | 1,766,663.02 | 15,082.63 | 0.00E+00 | Deformed wing virus isolate PA, complete genome |
|  | comp40733_c1_seq1 | 4,483 | 3,830,279.57 | 40,379.47 | 0.00E+00 | Deformed wing virus isolate Warwick-2009 polyprotein gene, complete cds |
| **Nymph #1** | comp13638_c0_seq1 | 237 | 17,000.17 | 3,878.68 | 1.00E-97 | Synthetic construct clone DWVinfDVD5 polyprotein gene, complete cds |
|  | comp15622_c0_seq1 | 3,434 | 13,456,670.58 | 161,609.76 | 0.00E+00 | Deformed wing virus isolate Warwick-2009 polyprotein gene, complete cds |
|  | comp15622_c1_seq1 | 6,745 | 6,910,437.87 | 41,877.37 | 0.00E+00 | Deformed wing virus isolate PA, complete genome |
| **Nymph #2** | comp20623_c0_seq1 | 313 | 893.17 | 517.96 | 2.00E-130 | Deformed wing virus isolate JP6 polyprotein gene, partial cds |
|  | comp20623_c0_seq2 | 202 | 40 | 66.83 | 1.00E-80 | Kakugo virus genomic RNA, complete genome |
|  | comp20623_c0_seq3 | 313 | 1,259.57 | 730.44 | 5.00E-133 | Deformed wing virus isolate YU6 polyprotein gene, partial cds |
|  | comp20996_c0_seq1 | 10,147 | 8,060,690.87 | 66,725.67 | 0.00E+00 | Kakugo virus genomic RNA, complete genome |

Supplementary Table 27. Ratios of DWV RNA to *Ef-1α* mRNA in the adult females, adult males, and nymphs

| **Sample** | **Ratio of the transcript numbers of DWV RNA to *Ef-1α* mRNA** | **Ratio of the FPKMs of DWV RNA to *Ef-1α* mRNA** |
| --- | --- | --- |
| **Males** | 446 | 692 |
| **Females** | 92 | 131 |
| **Nymphs** | 392 | 226 |

The ratios of the transcript numbers of DWV RNA to *Ef-1α* mRNA determined by qRT-PCR and the average ratios of the FPKMs of DWV RNA to *Ef-1α* mRNA mRNA in the adult males, adult females, and nymphs replicates are shown.

Supplementary Table 28. Primer sets used for qRT-PCR

| **Gene ID** | **Function protein** | **Forward primer (5' -> 3')** | **Reverse primer (5' -> 3')** |
| --- | --- | --- | --- |
| **Internal reference** |  |  |  |
| Tm_14054 | Elongation factor-1 alpha | ATTCCGGTAAGTCAACCACCAC | GCTCGGCCTTCAGTTTGTCCAA |
| **Tested genes** |  |  |  |
| DWV | - | GGAATAAAACCTCACA | GCTCGGCCTTCAGTTTGTCCAA |
| Tm_08036 | Peropsin | CCTGGCTGGACGGTGGCTCTACGGT | GATAACGTTGTGAGGGCGCAGCAT |
| Tm_15229 | Ionotropic glutamate receptor | TTCACTTGGGATACTTGGCTAGC | GCACCGAAGCAGTACCACTCGCA |
| Tm_15231 | Ionotropic glutamate receptor | GGAGACCTCGTCAAAAAGAAAGCGG | TGTCTCGAGCACTGTCAGGAACTTG |
| Tm_15244 | Unclassified glutamate receptor | AGTTACGGCTGTATGCCAGTTGATG | GCCCGATATTGAAGCTGAGAATGAA |

**References**

1.	Hoy MA, Waterhouse RM, Wu K, Estep AS, Ioannidis P, Palmer WJ, Pomerantz AF, Simão FA, Thomas J, Jiggins FM, et al: Genome Sequencing of the Phytoseiid Predatory Mite Metaseiulus occidentalis Reveals Completely Atomized Hox Genes and Superdynamic Intron Evolution. *Genome Biol Evol* 2016, 8:1762-1775.

2.	Hayes JD, Flanagan JU, Jowsey IR: Glutathione transferases. *Annu Rev Pharmacol Toxicol* 2005, 45:51-88.

3.	Oakeshott JG, Johnson RM, Berenbaum MR, Ranson H, Cristino AS, Claudianos C: Metabolic enzymes associated with xenobiotic and chemosensory responses in Nasonia vitripennis. *Insect Mol Biol* 2010, 19 Suppl 1:147-163.

4.	Wu K, Hoy MA: The Glutathione-S-Transferase, Cytochrome P450 and Carboxyl/Cholinesterase Gene Superfamilies in Predatory Mite Metaseiulus occidentalis. *PloS one* 2016, 11:e0160009.

5.	Grbić M, Van Leeuwen T, Clark RM, Rombauts S, Rouzé P, Grbić V, Osborne EJ, Dermauw W, Ngoc PCT, Ortego F: The genome of Tetranychus urticae reveals herbivorous pest adaptations. *Nature* 2011, 479:487-492.

6.	Yu QY, Lu C, Li WL, Xiang ZH, Zhang Z: Annotation and expression of carboxylesterases in the silkworm, Bombyx mori. *BMC Genomics* 2009, 10:553.

7.	Dermauw W, Van Leeuwen T: The ABC gene family in arthropods: comparative genomics and role in insecticide transport and resistance. *Insect biochemistry and molecular biology* 2014, 45:89-110.

8.	Pomerantz AF, Hoy MA, Kawahara AY: Molecular characterization and evolutionary insights into potential sex-determination genes in the western orchard predatory mite Metaseiulus occidentalis (Chelicerata: Arachnida: Acari: Phytoseiidae). *Journal of Biomolecular Structure and Dynamics* 2015, 33:1239-1253.
