# Supplementary material for: Draft genome of the honey bee ectoparasitic mite, Tropilaelaps mercedesae, is shaped by the parasitic life history
Source: Gigascience. 2017 Feb 22;6(3):1–17. doi: 10.1093/gigascience/gix008 (PMC5467014; doi:10.1093/gigascience/gix008)
Supplement: Supplemental material — Supplementary data are available at GIGSCI online. [file gix008_Supp.zip › Dong-et-al_Supplementary-Figures.doc]

**A**

**B**

**Supplementary Figure 1. K-mer (k=31) analysis for estimating the genome sizes of *T. mercedesae* male and female.**

The depth of k-mers is plotted against the frequency at which they occur (both X-axis and Y-axis are logarithmic scale). The upper left peaks represent the unique and rare k-mers produced by sequencing errors and are therefore not relevant to assess the genome size and architecture. The principal peaks at middle, representing in single copy of k-mers in the underlying genome, is proportional to sequencing coverage. The long tails following the principal peak primarily reflect the various classes of repetitive sequences that are present at different copy numbers in the mite genome [1]. The total k-mer numbers are 26,403,666,231 and 28,906,528,626, and the maximal frequencies are 40 and 46 for (A) male and (B) female, respectively. The genome sizes were estimated to be 660 Mb for male and 628 Mb for female. A high proportion (49.8 % of both male and female) of 31-mer sequences with the depth higher than 200X suggests that the genomes contain many repetitive sequences with high sequence similarity.

**A**

**B**

**C**

**Supplementary Figure 2. Nuclear DNA contents of *T. mercedesae* male and female.**

Histograms of the number of nuclei with red fluorescent (FL2-A) recorded by flow cytometry of propidium iodide stained nuclei from (A) *D. melanogaster* and *A. mellifera,* (B) *D. melanogaster*, *A. mellifera,* and *T. mercedesae* male, and (C) *D. melanogaster*, *A. mellifera*, and *T. mercedesae* female.

**Supplementary Figure 3. Genome sizes plot against gene densities of *T. mercedesae* and six other sequenced arthropod genomes.**

Genome size and gene density of each arthropod species are plotted on x and y axes, respectively.

**Supplementary Figure 4. Comparison of the sizes of exons and introns among seven sequenced arthropod genomes including *T. mercedesae*.**

(A) The x-axis indicates size (bp) of exon and the y-axis indicates the percentage of genes that have the corresponding size. (B) Intron length distribution of each arthropod species is shown by a box plot.

**Supplementary Figure 5. Gene family contraction and expansion in seven arthropod species.**

No outgroup was applied for the gene family analysis with CAFE. The purple branches represent the Arachnids. The numbers of expanded, contacted, and stable gene families in each species and node are indicated by red, green, and blue, respectively.

**Supplementary Figure 6. Phototransduction pathway components in *T. mercedesae*.**

Phototransduction pathway components of fruit fly are shown. Green boxes indicate the genes present in *T. mercedesae* genome, whereas those in white boxes are absent.

**Supplementary Figure 7. Phylogeneny of opsins in various animal species.**

The phylogenetic tree of opsin family [2] was inferred by the neighbor-joining method. Red, green, and blues branches represent opsins present in *T. mercedesae*, *M. occidentalis*, and *I. scapularis*, respectively. The number at each branch node represents the bootstrap probability (bootstrap=1000). The phylogenetically distinct clusters (subfamilies) and their names are shown on the right side of the tree. Known chromophore configuration in the dark state of members of each subfamily is indicated in parentheses.

**Supplementary Figure 8. *Peropsin* mRNA expression in *T. mercedesae*.**

(A) *Peropsin* (Tm_08036) mRNA levels in the adult females and males were analyzed by RNA-seq and shown by average RPKM (reads per kilobase of exon per million mapped sequence reads) values of the replicates. (B) The relative expression values were also determined by qRT-PCR. (C) The relative mRNA levels of Tm_08036 in the first legs, 2nd-4th legs, and whole body without legs are shown. Error bars indicate the standard deviations.

**Supplementary Figure 9. Expression of two *T. mercedesae* GR mRNAs in the male, female, and nymph.**

The levels of two *T. mercedesae* GR (Tm_09509 and Tm_03548) mRNAs in the adult males, adult females, and nymphs were measured by RNA-seq, and are shown by average RPKM values of the replicates.

**Supplementary Figure 10. Phylogenetic tree of *T. mercedesae*, *M. occidentalis*, and *D. melanogaster* ionotropic receptors and ionotropic glutamate receptors.**

Phylogenetic tree of *T. mercedesae* (red), *I. scapularis* (blue), and *D. melanogaster* (green) ionotropic receptors (IRs) and ionotropic glutamate receptors (iGluRs) was constructed by a maximum likelihood method. IRs and iGluRs are classified to two and six groups, respectively as shown.

**B**

**A**

**Supplementary Figure 11. Relative mRNA levels of two IRs in the different body parts of mite.**

Relative mRNA levels of two IRs, (A) Tm_15229 and (B) Tm_15231, in the 1st legs, 2nd-4th legs, and main body of *T. mercedesae* measured by qRT-PCR are shown.

**Supplementary Figure 12. Expression profile of cytochrome P450 genes in adult male, adult female, and nymph of *T. mercedesae* by a heat map.**

The mRNA levels were measured by RNA-seq and are shown by RPKM values using normalized library sizes. The genes are clustered according to their expression profiles. Here, the ‘expressed gene’ was defined as the gene with at least one count per million in the replicates. Asterisks (_*_), (_**_) and (_***_) refer to genes with the significant up-regulation of mRNA expression (FDR *P*-value < 0.05) detected by one and two particular pair wise comparisons, respectively.

**Supplementary Figure 13. Phylogeny of *T. mercedesae*, *M. occidentalis*, *D. melanogaster*, and *T. urticae* GST proteins.**

Phylogenetic tree of *T. mercedesae* (red), *M. occidentalis* (blue), *D. melanogaster* (green), and *T. urticae* (yellow) GST proteins was constructed by a maximum likelihood method. The number at each branch node represents the bootstrap probability. The phylogenetically distinct clusters (subfamilies) and their names are shown at the right side of tree.

**Supplementary Figure 14. Phylogeny of *T. mercedesae*, *M. occidentalis*, *D. melanogaster*, and *B. mori* CCE proteins.**

Phylogenetic tree of *T. mercedesae* (red), *M. occidentalis* (yellow), *D. melanogaster* (green), and *Bombyx mori* (blue) CCE proteins was constructed by a maximum likelihood method. The number at each branch node represents the bootstrap probability. The phylogenetically distinct clusters (subfamilies) and their names are shown at the right side of tree.

**Supplementary Figure 15. Phylogeny of *T. mercedesae*, *M. occidentalis,* and *D. melanogaster* ABC transporters.**

Phylogenetic tree of *T. mercedesae* (red), *M. occidentalis* (blue)*,* and *D. melanogaster* (green) ABC transporters was constructed by a maximum likelihood method. The ABC transporters were divided into nine groups, A-H and an unknown group (black branches) as shown. The tree was rooted at the middle point.

**Supplementary Figure 16. Phylogeny of Dmrt proteins of *T. mercedesae* and other arthropods.**

Phylogenetic tree of *M. occidentalis* (Mocc), *I. scapularis* (Iscap), *D. melanogaster* (Dmel), *D. pseudoobscura* (Dpseu), *C. capitata* (Ccap), *M. domestica* (Mdom), *T. castaneum* (Tcas), *B. mori* (Bmor), *A. mellifera* (Amel), *D. magna* (Dmag), and *D. pulex* (Dpul) Dmrt proteins [3] was constructed by a neighbor-joining method. *T. mercedesae* proteins are highlighted with purple. Classification of each cluster is designated on the right. The number at the node of each branch represents the bootstrap value (bootstrap=1000).

**Supplementary Figure 17. Expression profiles of *T. mercedesae* *transformer-2* and *dmrt* in adult females, adult males, and nymphs.**

The mRNA levels of *T. mercedesae* *transformer-2* (Tm_09923) and *dmrt* (Tm_02277, Tm_04084, Tm_05831, Tm_07872, and Tm_08581) in adult females (red), adult males (blue), and nymphs (green) were measured by RNA-seq and are shown with the average RPKM value of replicates.

**Supplementary Figure 18. Confirmation of the integration of partial *Wolbachia* DNA into the mite genome.**

(A) The presence of partial *Wolbachia* DNA in the male mite genome was confirmed by PCR using contig_182068 and contig_188982 as the examples. The sizes of amplicons are the same as those expected from the contig sequences. (B) The positions of exons encoding the mite genes (green arrows) and partial *Wolbachia* genes (red arrows) identified by Blastx (1E-10) as well as forward and reverse primers are shown in the genomic contig_182068 and contig_188982. The arrows point to 3' direction. The primer sets used are 5'-GCGAACACACATTATCCCCTTCCGCGCA-3' (forward, contig_182068) and 5'-TCAGATCAGACGCCATACTGAAGCTGAG-3' (reverse, contig_182068) as well as 5'-AACACGTATACTCGCACGTGAAGTACGG-3' (forward, contig_188982) and 5'- ATGCAAGCAAACAATATGGGGAGTCAGC-3' (reverse, contig_188982).

```
>tr|Q8B3M2|Q8B3M2_9VIRU Genome polyprotein OS=Deformed wing virus
   10         20         30         40         50
MAFSCGTLSY SAVAQAPSVA YAPRTWEVDE ARRRRVIKRL ALEQERIRNV
        60         70         80         90        100
LDVAVYDQAT WEQEDARDNE FLTEQLNNLY TIYSIAERCT RRPIKEXSPI
       110        120        130        140        150
SVSNRFAPLE SLKVEVGQEA XECXFKKPKY TRXCKKVKRV ATRFVREKVV
       160        170        180        190        200
RPMCSRSPML LFKLKKIIYD LHLYRLRKQI RMLRRQKQRD YELECVTNLL
       210        220        230        240        250
QLSNPVQAKP EMDNPNPGPD GEGEVELEKD SNVVLTTQRD PSTSIPAPVS
       260        270        280        290        300
VKWSRWTSND VVDDYATITS RWYQIAEFVW SKDDPFDKEL ARLILPRALL
       310        320        330        340        350
SSIEANSDAI CDVPNTIPFK VHAYWRGDME VRVQINSNKF QVGQLQATWY
       360        370        380        390        400
YSDHENLNIS SKRSVYGFSQ MDHALISASA SNEAKLVIPY KHVYPFLPTR
       410        420        430        440        450
IVPDWTTGIL DMGALNIRVI APLRMSATGP TTCNVVVFIK LNNSEFTGTS
       460        470        480        490        500
SGKFYASQIR AKPEMDRILN LAEGLLNNTI GGNNMDNPSY QQSPRHFVPT
       510        520        530        540        550
GMHSLALGTN LVEPLHALRL DAAGTTQHPV GCAPDEDMTV SSIASRYGLI
       560        570        580        590        600
RRVQWKKDHA KGSLLLQLDA DPFVEQRIEG TNPISLYWFA PVGVVSSMFM
       610        620        630        640        650
QWRGSLEYRF DIIASQFHTG RLIVGYVPGL TASLQLQMDY MKLKSSSYVV
       660        670        680        690        700
FDLQESNSFT FEVPYVSYRP WWVRKYGGNY LPSSTDAPST LFMYVQVPLI
       710        720        730        740        750
PMEAVSDTID INVYVRGGSS FEVCVPVQPS LGLNWNTDFI LRNDEEYRAK
       760        770        780        790        800
TGYAPYYAGV WHSFNNSNSL VFRWGSXSDQ IAQWPTISVP RGELAFLRIK
       810        820        830        840        850
DGKQAAVGTQ PWRTMVVWPS GHGYNIGIPT YNAERARQLA QHLYGGGSLT
       860        870        880        890        900
DEKAKQLFVP ANQQGPGKVS NGNPVWEVMR APLATQRAHI QDFEFIEAIP
       910        920        930        940        950
EGEESRNTTV LDTTTTLQSS GFGRAFFGEA FNDLKTLMRR YQLYGQLLLS
       960        970        980        990       1000
VTTDKDIDHC MFTFPCLPQG LALDIGSAGS PHEIFNRCRD GIIPLIASGY
      1010       1020       1030       1040       1050
RFYRGDLRYK IVFPSNVNSN IWVQHRPDRR LEGWSAAKIV NCDAVSTGQG
      1060       1070       1080       1090       1100
VYNHGYASHI QITRVNNVIE LEVPFYNATC YNYLQAFNAS SAASSYAVSL
      1110       1120       1130       1140       1150
GEISVGFQAT SDDIASIVNK PVTIYYSIGD GMQFSQWVGY QPMMILDQLP
      1160       1170       1180       1190       1200
APVVRAVPEG PIAKIKNFFH QTADEVREAQ AAKMREDMGM VVQDVIGELS
      1210       1220       1230       1240       1250
QAIPDLQQPE VQANVFSLVS QLVHAIIGTS LKTVAWAIVS IFVTLGLIGR
      1260       1270       1280       1290       1300
EMMHSVITVV KRLLEKYHLA TQPQESASSS TVISAVPEAP NAEAEEASAW
      1310       1320       1330       1340       1350
VSIIYNGVCN MLNVAAQKPK QFKDWVKLAT VDFSNNCRGS NQVFVFFKNT
      1360       1370       1380       1390       1400
FEVLKKMWGY VFCQSNPAAR LLKAVNDEPE ILKAWVKECL YLDDPKFRMR
      1410       1420       1430       1440       1450
RAHDQEYIER VFAAHSYGQI LLHDLTAEMN QSRNLSVFTR VYDQISKLKT
      1460       1470       1480       1490       1500
DLMEMGSNPY IRRECFTICM CGASGIGKSY LTDSLCSELL RASRTPVTTG
      1510       1520       1530       1540       1550
IKCVVNPLSD YWDQCDFQPV LCVDDMWSVE TSTTLDKQLN MLFQVHSPIV
      1560       1570       1580       1590       1600
LSPPKADLEG KKMRYNPEIF IYNTNKPFPR FDRIAMEAIY RRRNVLIECK
      1610       1620       1630       1640       1650
ASEEKKRGCK HCENDIPIAE CSPKMLKDFH HIKFRYAHDV CNSETTWSEW
      1660       1670       1680       1690       1700
MTYNEFLEWI TPVYMANRRK ANESFKMRVD EMQMLRMDEP LEGDNILNKY
      1710       1720       1730       1740       1750
VEVNQRLVEE MKAFKERTLW SDLHRVGAEI SASVKKALPT ISITEKLPHW
      1760       1770       1780       1790       1800
TVQCGIAKPE MDHAYEVMSS YAAGMNAEIE AHEQVRRSSV ECQFAEPQAX
      1810       1820       1830       1840       1850
RNPDDEGPTI DEELMGDTEF TSQALERLVD EGYITGKQKK YIAMWCSKRR
      1860       1870       1880       1890       1900
EHTADFDLVW TDNLRVLSAY VHERSSSTRL STDDVKLYKT ISMLHQKYDT
      1910       1920       1930       1940       1950
TECAKCQHWY APLTDIYVDD KKLFWCQKEK KTLIDVRKLS KEDVTVQSKL
      1960       1970       1980       1990       2000
XNLSVPCGEV CMLHSKYFNY LFHKAWLFEN PTWRLIYNGT KKGMPEYFMN
      2010       2020       2030       2040       2050
CVDEISLDSK FGKVKVWLQA IIDKYLTRPV KMIRDFLFKW WPQVAYVLSL
      2060       2070       2080       2090       2100
LGIIGITAYE MRNPKPTSEE LADHYVNRHC SSDFWSPGLA SPQGLKYSEA
      2110       2120       2130       2140       2150
VTVKAPRIHR LPVTTKPQGS TQQVDAAVNK ILQNMVYIGV VFPKVPGSKW
      2160       2170       2180       2190       2200
RDINFRCLML HNRQCLMLRH YIESTAAFPE GTKYYFKYIH NQETRMSGDI
      2210       2220       2230       2240       2250
SGIEIDLLNL PRLYYGGLAG EESFDSNIVL VTMPNRIPEC KSIIKFIASH
      2260       2270       2280       2290       2300
NEHIRAQNDG VLVTGDHTQL LAFENNNKTP ISINADGLYE VILQGVYTYP
      2310       2320       2330       2340       2350
YHGDGVCGSI LLSRNLQRPI IGIHVAGTEG LHGFGVAEPL VHEMFTGKAI
      2360       2370       2380       2390       2400
ESEREPYDRV YELPLRELDE SDIGLDTDLY PIGRVDAKLA HAQSPSTGIK
      2410       2420       2430       2440       2450
KTLIHGTFDV RTEPNPMSSR DPRIAPHDPL KLGCEKHGMP CSPFNRKHLE
      2460       2470       2480       2490       2500
LATNHLKEKL VSVVKPINGC KIRSLQDAXC GVPGLDGFDS ISWNTSAGFP
      2510       2520       2530       2540       2550
LSSLKPPGTS GKRWLFDIEL QDSGCYLLRG MRPELEIQLS TTQLMRKKGI
      2560       2570       2580       2590       2600
KPHTIFTDCL KDTCLPVEKC RIPGKTRIFS ISPVQFTIPF RQYYLDFMAS
      2610       2620       2630       2640       2650
YRAARLNAEH GIGIDVNSLE WTNLATRLSK XGTHIVTGDY KNFGPGLDSD
      2660       2670       2680       2690       2700
VAASAFEIII DWVLHYTEED NKDEMKRVMW TMAQEILAPS HLYRDLVYRV
      2710       2720       2730       2740       2750
PCGIPSGSPI TDILNTISNC LLIRLAWLGI TDLPLSEFSQ NVVLVCYGDD
      2760       2770       2780       2790       2800
LIMNVSDNMI DKFNAVTIGK FFSQYKMEFT DQDKSGNTVK WRTLQTATFL
      2810       2820       2830       2840       2850
KHGFLKHPTR PVFLANLDKV SVEGTTNWTH ARGLGRRTAT IENAKQALEL
      2860       2870       2880       2890
AFGWGPEYFN YVRNTIKMAF DKLGIYEDLI TWEEMDVRCY ASA
```

**Supplementary Figure 19. Proteomic detection of DWV peptides in the male and female mites.**

Peptides mapped to DWV polyprotein in the mites are shown by red. The capsid (structural) proteins (VP1-VP4) are located between 218-1159, and the non-structural viral proteins (helicase, genome-linked viral protein, 3C-protease, and RNA-dependent RNA polymerase) are located between 1288-2893.

**References**

1.	Moeller AH, Li Y, Mpoudi Ngole E, Ahuka-Mundeke S, Lonsdorf EV, Pusey AE, Peeters M, Hahn BH, Ochman H: Rapid changes in the gut microbiome during human evolution. *Proc Natl Acad Sci U S A* 2014, 111:16431-16435.

2.	Nagata T, Koyanagi M, Tsukamoto H, Terakita A: Identification and characterization of a protostome homologue of peropsin from a jumping spider. *J Comp Physiol A Neuroethol Sens Neural Behav Physiol* 2010, 196:51-59.

3.	Pomerantz AF, Hoy MA, Kawahara AY: Molecular characterization and evolutionary insights into potential sex-determination genes in the western orchard predatory mite Metaseiulus occidentalis (Chelicerata: Arachnida: Acari: Phytoseiidae). *J Biomol Struct Dyn* 2015, 33:1239-1253.
